# Transcriptome and metabolite analyses indicated the underlying molecular responses of Asian ginseng (*Panax ginseng*) toward *Colletotrichum panacicola* infection

**DOI:** 10.3389/fpls.2023.1182685

**Published:** 2023-07-10

**Authors:** Jinglin Xia, Ning Liu, Junyou Han, Jingyuan Sun, Tianyi Xu, Shouan Liu

**Affiliations:** ^1^ Laboratory of Tea and Medicinal Plant Biology, Jilin University, Changchun, China; ^2^ Institute of Special Animal and Plant Science, Chinese Academy of Agricultural Sciences, Changchun, China

**Keywords:** *Panax ginseng*, *Colletotrichum panacicola*, transcriptome, anthracnose disease, flavonoids

## Abstract

*Panax ginseng* Meyer is one of the most valuable plants and is widely used in China, while ginseng anthracnose is one of the most destructive diseases. *Colletotrichum panacicola* could infect ginseng leaves and stems and causes serious anthracnose disease, but its mechanism is still unknown. Here, transcriptome and metabolism analyses of the host leaves were conducted to investigate the ginseng defense response affected by *C. panacicola*. Upon *C. panacicola* infection, ginseng transcripts altered from 14 to 24 h, and the expression of many defense-related genes switched from induction to repression. Consequently, ginseng metabolites in the flavonoid pathway were changed. Particularly, *C. panacicola* repressed plant biosynthesis of the epicatechin and naringin while inducing plant biosynthesis of glycitin, vitexin/isovitexin, and luteolin-7-*O*-glucoside. This work indicates *C. panacicola* successful infection of *P. ginseng* by intervening in the transcripts of defense-related genes and manipulating the biosynthesis of secondary metabolites, which might have antifungal activities.

## Introduction

Ginseng, *Panax ginseng*, is one of the most important medicinal herbs in Northeast China with high values in nutrition and medicine (Kim et al., 2010; Liu et al., 2020). Ginseng is a perennial plant with a long cultivation period and is widely planted in China, Korea, and Japan (Sun et al., 2018; Wang et al., 2018). Because of its long life, *P. ginseng* now suffers from various biotic stresses with fungal diseases as one of the main sources (Kim et al., 2019). There are many fungal diseases in ginseng fields including anthracnose, gray mold, *Alternaria* blight, damping-off, and root rot (Kim et al., 2010; Choi et al., 2011; Gao et al., 2014; Guan et al., 2014; Liu et al., 2020; Chen et al., 2022; Lu et al., 2019). Among these, anthracnose is one of the most destructive diseases, causing ginseng seedling blight in nurseries and promoting ginseng leaf spots in permanent beds (Choi et al., 2011; Chung and Bae, 1979).

*Colletotrichum panacicola* was reported as a major fungal pathogen causing ginseng anthracnose disease in Korea (Choi et al., 2011). Recently, *C. panacicola* was confirmed in all provinces in Northeast China such as Jilin, Liaoning, and Heilongjiang (Liu et al., 2020). The *C. panacicola* was able to infect both ginseng leaves and stems more aggressively than other species (Liu et al., 2020). Usually, chemical pesticides were used to control ginseng disease. However, deleterious pesticide residues accumulate in both ginseng roots and the surrounding soil, which has become a serious environmental problem (Ryu et al., 2014). The outcome of the interaction between ginseng and *C. panacicola* has not been well characterized so far. Detailed molecular and comparative transcriptome analyses during ginseng–*C. panacicola* interactions are becoming feasible, as the genome of *P. ginseng* has been reported (Jayakodi et al., 2018).

During plant–microbe interaction, plants have developed an innate immune system to recognize potentially infectious pathogens (Jones and Dangl, 2006; Jones et al., 2016; Ngou et al., 2021; Yuan et al., 2021). Downstream of innate recognition, the complex defense signaling/responses are activated including, calcium (Ca^2+^) burst, reactive oxygen species (ROS) burst, mitogen‐activated protein kinase (MAPK) activation, plant hormone signaling, transcription factors (TFs), and secondary metabolism (Tsuda et al., 2009; Birkenbihl et al., 2017; Wang et al., 2020). Generally, plant hormones such as salicylic acid (SA), jasmonate (JA), ethylene (ET), and abscisic acid (ABA) are repeatedly observed upon pathogen infection (Bari and Jones, 2009; Chen and Yu, 2014; Verma et al., 2016; Chen et al., 2020). Depending on the pathogen and plant styles, the role of hormones is different from time to time (Glazebrook, 2005; Liu et al., 2015; Liu et al., 2017). SA signaling has been traditionally associated with defense against hemibiotrophic or biotrophic pathogens, whereas JA/ET signaling appears to be more important to necrotrophic pathogens and insects (Glazebrook, 2005). The JA signaling was also involved in plant–biotrophic pathogen interaction, while SA/JA signaling collaborated during effectors that triggered immunity response (Guerreiro et al., 2016; Liu et al., 2016). ABA signaling has emerged as an important modulator of plant stress networks (Chen and Yu, 2014; Chen et al., 2020). In plants, ABA positively or negatively regulates plant defense depending on the lifestyle of the pathogen (Denancé et al., 2013).

Global expression analyses proposed that plant cells’ transcriptional re-programming is a key step to mounting an efficient defense response (Birkenbihl et al., 2017; Chen et al., 2022; Liu et al., 2015). Several transcription factor families are repeatedly reported to be involved in the regulation of gene expression during plant–pathogen interaction (Birkenbihl et al., 2017). Next to the plant hormones and TFs, plant secondary metabolites play crucial roles in defense. Low-molecular-mass secondary metabolites with antimicrobial activity that are induced by various microbes are called phytoalexins (Pedras et al., 2011), which are usually considered molecular markers of disease resistance (Ahuja et al., 2012). Compared with many plant–microbe interaction systems, however, so far, there has been no such information reported during ginseng–*C. panacicola* interaction. Here, the *C. panacicola* was assumed to derive a strategy to overcome ginseng defense response and promote disease development.

This work performed transcriptome analysis in ginseng leaves during *C. panacicola* infection. A further study compared the data derived from the same treatments and revealed the mechanism of disease development in *C. panacicola* by suppressing defense-related gene expression and modulating certain antifungal flavonoid biosynthesis. These results will not only increase understanding of the complexity of *P. ginseng*–*C. panacicola* interaction but also enhance efforts to identify functional genes in plant defense response and disease development.

## Results

### Phenotypes of anthracnose disease development in ginseng

**Figure 1:**
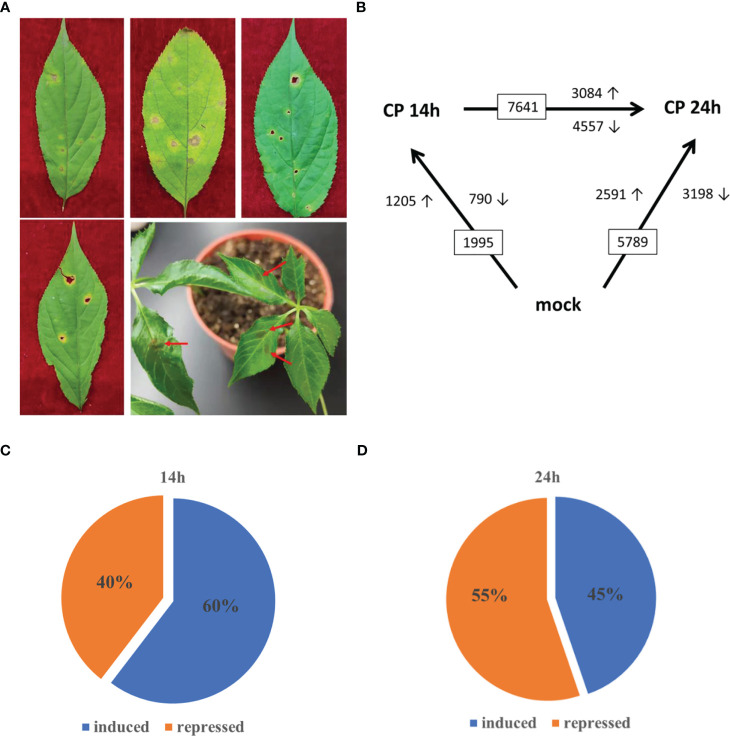
Transcription analysis of *Colletotrichum panacicola*-infected *Panax ginseng* plants. **(A)** Ginseng anthracnose disease phenotype in the forest and *C. panacicola* FSL infection phenotype on 2-year-old ginseng leaves (7 days post-infection). **(B)** Numbers of differentially expressed genes (≥2-fold; *p* ≤ 0.05) in ginseng at 14 and 24 h after spray inoculation with spores of *C. panacicola* FSL (CP) or mock treatment (CK) identified by RNA-seq. **(C)** Percentage of *C. panacicola* FSL induced and repressed significantly differentially expressed genes at 14 h compared with control (total number of SSTF gene is 1,995). **(D)** Percentage of *C. panacicola* FSL induced and repressed significantly differentially expressed genes at 24 h compared with control (total number of SSTF gene is 5,789).

In nature, *P. ginseng* grows slowly and faces multiple biotic and abiotic stresses including anthracnose. In the field, the anthracnose disease in ginseng leaves usually contains several tiny spots with a small yellow halo at the beginning, the lesion develops, the yellow halo extends from time to time, and finally, necrotic spots with an empty hole in the middle area are present in ginseng leaves (**Figure 1A**). The spots with empty holes seriously influence the growth of ginseng leaves. *C. panacicola* strain FSL was isolated from ginseng leaves in Fusong, Jilin Province. The spores of *C. panacicola* FSL were then incubated on garden ginseng leaves in the lab. At the early stage, there was no obvious symptom observed on ginseng leaves (**Supplementary Figure 1**). The typical necrotic spots were observed after a 1-week infection (**Figure 1A**, arrows). It is indicated that ginseng is susceptible to *C. panacicola* FSL.

### Transcriptome analysis of differentially expressed genes in *P. ginseng* during interaction with *C. panacicola*

To understand the molecular mechanism of *P. ginseng*–*C. panacicola* interaction, RNA sequencing was performed. Ginseng leaves incubated with *C. panacicola* were harvested at 14 h (Cp14h) and 24 h (Cp24h). For the control (CK), ginseng leaves without any treatment were harvested. RNA-seq data were analyzed as previously indicated (Chen et al., 2022). A total of 365 million validated high-quality reads were obtained from all nine libraries (**Supplementary File 1**) and then aligned to the *P. ginseng* genome (Jayakodi et al., 2018).

To identify genes involved in ginseng response to *C. panacicola* at the genome-wide level under different times, the differentially changed genes (statistically significantly, *p* ≤ 0.05, altered at least twofold (SSTF)) were compared between *C. panacicola*-treated (CP) and un-treated (CK) ginseng. At 14 h, a total of 1,995 SSTF genes were identified in CP-treated plants compared with CK: 1,205 (approximately 60%) were increased, while 790 (40%) were decreased (**Figures 1B, C**). Not surprisingly, a total of 5,789 SSTF genes were identified at 24 h compared with CK, which were about threefold more than at 14 h (**Figure 1B**). This indicates that more genes were affected in response to *C. panacicola* treatment. To our surprise, approximately 55% of genes (3198) were repressed while 45% of genes (2591) were induced (**Figure 1C**), suggesting the balance between induction and repression of host genes changed from 14 to 24 h (**Figures 1C, D**). The comparison of transcripts between 24 and 14 h was also performed. As indicated in **Figure 1B**, 7,641 SSTF genes were observed, with 3,084 (40.4%) increased and 4,557 (59.6%) decreased, further confirming the repression of host genes by *C. panacicola* at 24 h.

### Transcriptome diversity in *P. ginseng* upon *C. panacicola* infection at different times

Since pathogen incubation often up- or downregulates host gene expressions during the interaction, the transcription diversity was further analyzed in ginseng upon *C. panacicola* infection at 14 and 24 h. The differentially expressed genes (DEGs) were analyzed by Gene Ontology (GO) and Kyoto Encyclopedia of Genes and Genomes (KEGG) methods.

For the SSTF genes at 14 h, GO terms about defense response, protein serine/threonine kinase activity, cell wall, unsaturated fatty acid biosynthetic process, killing of cells of other organisms, and plasma membrane were enriched (**Supplementary Figures 2A**). KEGG analysis indicated the genes associated with the plant–pathogen interaction, metabolism, and biosynthesis were enriched (**Supplementary Figure 2B**). For the SSTF genes at 24 h, GO and KEGG terms about similar pathways were enriched as those at 14 h (**Supplementary Figures 3A, B**).

**Figure 2:**
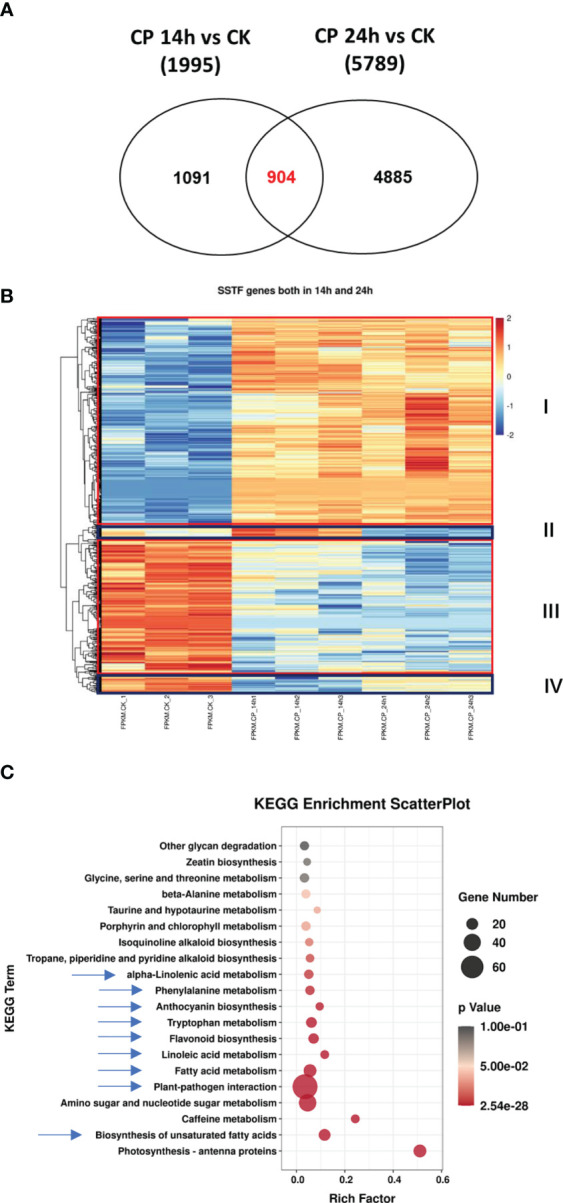
Characterization of the overlap genes upon *Colletotrichum panacicola* FSL infection at different times. **(A)** Venn diagram illustrating the total number and the number of common genes affected in ginseng 14 and 24 h post-*C. panacicola* FSL inoculation. **(B)** A heatmap analysis of common genes affected in ginseng 14 and 24 h post-*C. panacicola* FSL inoculation. **(C)** Kyoto Encyclopedia of Genes and Genomes (KEGG) analysis of common genes observed in ginseng at both 14 and 24 h post-*C. panacicola* FSL inoculation.

Next, when comparing the SSTF genes at both 14 and 24 h, 904 genes were observed. It was indicated that 904 genes were consistently significantly affected upon *C. panacicola* treatment, not only at 14 h but also at 24 h (**Figure 2A**). A heatmap analysis was performed, and different expression patterns among these 904 genes are shown in **Figure 2B**. The red-framed genes (I and III) indicated the differentially expressed genes in CK and CP treatment. SSTF genes in group I was induced upon CP infection compared with CK, while genes in group III were decreased. Interestingly, the highest expression level of genes in group II was observed in Cp14h, while the lowest expression level of genes in group IV was also observed in Cp14h. KEGG analysis of SSTF genes observed at both 14 and 24 h indicated that defense- and metabolism biosynthesis-related genes are enriched after *C. panacicola* infection. These pathways included plant–pathogen interaction, biosynthesis of unsaturated fatty acids, and metabolisms such as fatty acid, linoleic acid, alpha-linolenic acid, phenylalanine, anthocyanin, tryptophan, and flavonoid (**Figure 2C**). The enrichment of these pathways at both 14 and 24 h suggests their role in ginseng–*C. panacicola* interaction.

Similarly, when comparing healthy ginseng and red skin ginseng, GO enrichment analysis showed that DEGs were significantly more clustered into “response to chitin”, “transcription, DNA-templated”, and “defense response” terms, while the KEGG analysis indicated DEGs were greatly enriched in pathways such as “biosynthesis of unsaturated fatty acids” and “plant–pathogen interaction” (Luo et al., 2022). It was also reported that there was a significant difference in the soil microorganisms between healthy *P. ginseng* and heavily ginseng red skin root syndrome-diseased groups; the GO and KEGG term enrichment was very likely due to the different soil microorganisms (Dong et al., 2022). These studies together with our previous studies on ginseng–*Botrytis cinerea* interaction (Chen et al., 2022) revealed that the transcriptome associated with plant defense changed with diversity in *P. ginseng* upon *C. panacicola* infection.

### Ginseng defense pathway genes were repressed by *C. panacicola* from 14 to 24 h

Since *C. panacicola* infection downregulated approximately 59.6% of host genes 24 h after infection, we next wanted to know which pathways are involved. **Supplementary Figure 4** reveals the GO terms in biological process, cellular component, and molecular function at 14 and 24 h. The upregulated and downregulated genes in each GO term were also included. As mentioned previously, *C. panacicola* infection altered the balance between induction and repression of many genes in ginseng. **Supplementary Figure 4B** clearly indicates the repression of genes in many GO terms upon pathogen infection at 24 h. These included the nucleus, DNA-templated regulation of transcription, DNA-templated transcription, DNA-binding transcription factor activity, and DNA binding.

**Figure 3:**
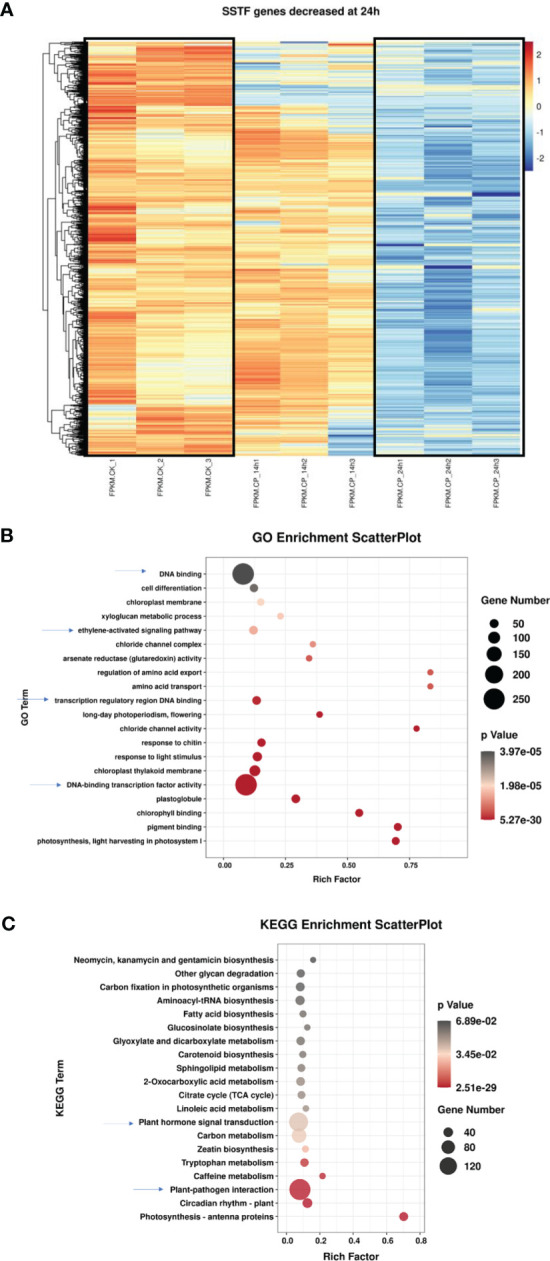
Characterization of the differentially expressed genes in ginseng upon *Colletotrichum panacicola* FSL infection at 24 h. **(A)** A heatmap analysis of differentially expressed genes in ginseng upon *C. panacicola* FSL infection. The indicated genes in ginseng were decreased at 24 h upon *C. panacicola* FSL infection. **(B)** Gene Ontology (GO) analysis of *C. panacicola* FSL repressed genes in ginseng at 24 h after infection. **(C)** Kyoto Encyclopedia of Genes and Genomes (KEGG) analysis of *C. panacicola* FSL repressed genes at 24 h after infection.

We next analyzed the downregulated SSTF genes at 24 h. Approximately 3,200 genes were used in the heatmap analysis. As shown in **Figure 3A**, most genes were significantly decreased at 24 h when compared with CK. However, the expression level of many genes at 14 h was not seriously decreased and even remained at a higher level. It suggests that the downregulation of these genes happened from 14 to 24 h.

To obtain detailed pathway information of the downregulated SSTF genes at 24 h, GO and KEGG analyses were further performed based on 3,200 genes (**Figures 3B, C**). Plant–pathogen interaction, plant hormone signal transduction, transcription factors, and ethylene-activated signaling pathways were observed, suggesting their potential role in disease development. Similarly, the downregulated genes associated with defense responses, defense response to fungus, certain TFs, secondary metabolite biosynthetic process, and plant–pathogen interaction are enriched during the compatible interaction between ginseng and *B. cinerea* (Chen et al., 2022). Since TFs and hormones play an important role in plant defense against pathogens, here, the downregulation of TFs and hormones pathway genes in ginseng by *C. panacicola* might contribute to disease development.

### Flavonoid biosynthesis pathway genes were differentially expressed in ginseng upon *C. panacicola* infection

**Figure 4:**
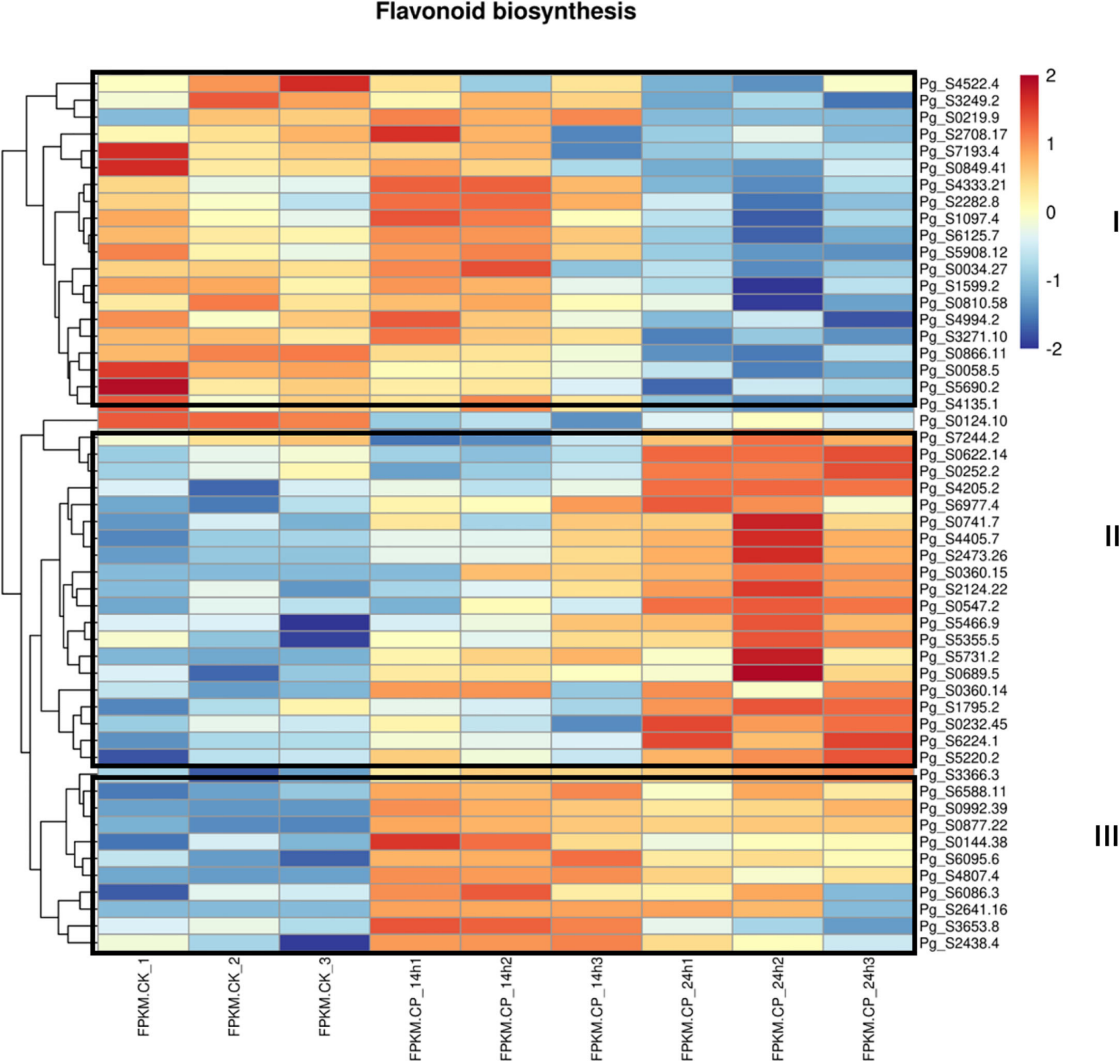
Heatmap analysis of differentially expressed genes in flavonoid pathway. Differentially expressed genes (DEGs) were observed in CK and *Colletotrichum panacicola* FSL-treated ginseng plants at 14 and 24 h.

Since secondary metabolites are involved in plant–pathogen interaction, the differentially expressed genes in the flavonoid biosynthesis pathway were checked. Genes involved in the flavonoid pathway were enriched at both 14 and 24 h. Approximately 52 genes were used for the heatmap analysis (**Figure 4**). At least three groups were observed. In group I, genes were decreased at 24 h when compared with CK and 14 h, suggesting their expression was repressed at a late stage. In group II, genes at 24 h were the highest compared with CK and 14 h, suggesting the induction of these genes at a late stage. In group III, the highest gene expression level was observed at 14 h, indicating an earlier response toward *C. panacicola*. The differential expression of genes in the flavonoid pathway suggests a differential role in ginseng–*C. panacicola* interaction. Similarly, when ginseng was attacked by *B. cinerea*, the GO term “regulation of flavonoid biosynthetic process” was enriched in downregulated genes (Chen et al., 2022). With this, many metabolites associated with flavonoids were decreased including kaempferol, quercetin, and luteolin. The decrease in flavonoids suggested that these compounds played a role in plant defense. Indeed, *B. cinerea* BcQdo is involved in the degradation of flavonoids such as kaempferol and quercetin, causing diseases (Chen et al., 2022).

### Quantification of differentially changed flavonoids in ginseng upon *C. panacicola* infection

**Figure 5:**
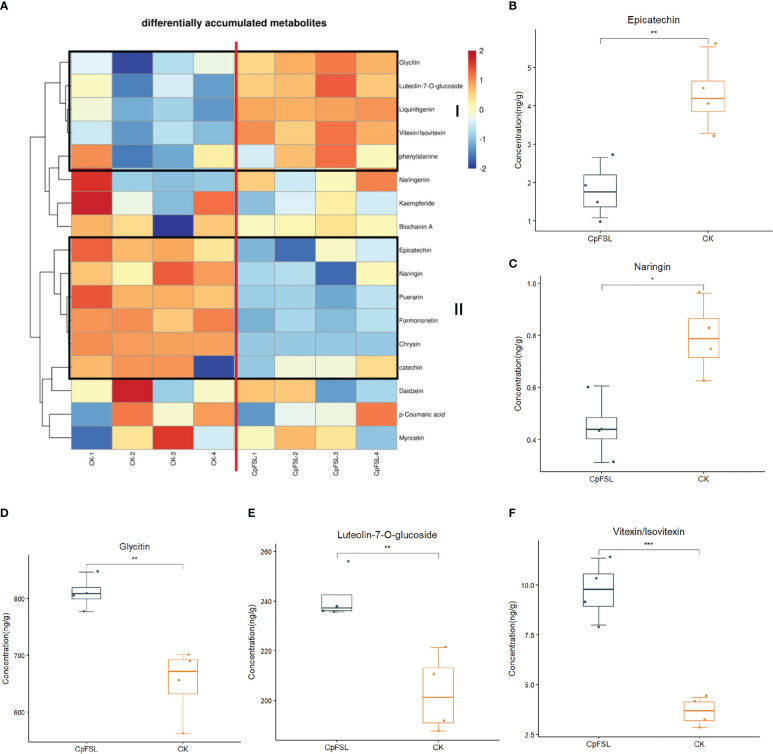
Differentially accumulated chemicals in ginseng plant associated with flavonoid pathway upon *Colletotrichum panacicola* FSL infection. **(A)** Heatmap analysis of differentially accumulated chemicals in flavonoid pathway upon *C. panacicola* FSL infection. **(B–F)** Differentially accumulated compounds in CK and *C. panacicola* FSL-treated ginseng including epicatechin **(B)**, naringin **(C)**, glycitin **(D)**, luteolin-7-*O*-glucoside **(E)**, and vitexin/isovitexin **(F)**. Four replicates were performed, and the significance between different treatments was indicated as * *p* < 0.05, ** *p* < 0.01 and *** *p* < 0.001.

Since genes in the flavonoid pathway are differentially expressed in ginseng upon *C. panacicola* infection, the study next analyzed if ginseng-related metabolites are affected. Two-year-old ginseng was sprayed with *C. panacicola* spores for 14 h (CpFSL) and harvested for ultrahigh-performance liquid chromatography–electrospray ionization–tandem mass spectrometry (UPLC-ESI-MS/MS) analysis (**Supplementary Table 1**; **Supplementary Figure 5**; **Supplementary File 2**). The plants without fungi treatment were used as control (CK). Four replicates were performed for each treatment. Seventeen metabolites in the flavonoid pathway were detected in ginseng leaves under this condition (**Supplementary Table 2**; **Supplementary File 2**). The heatmap of differentially accumulated compounds in the flavonoid pathway is shown in **Figure 5A**. At least two groups of differentially accumulated compounds were observed (framed). Group I indicated the increased chemicals in ginseng after *C. panacicola* infection, while group II indicated the decreased chemicals in ginseng. In particular, epicatechin, naringin, etc., were significantly decreased, suggesting that these compounds were suppressed by fungi (**Figures 5B, C**, **Supplementary Figures 6**, **7**, **Supplementary Table 2**). In contrast, glycitin, vitexin/isovitexin, luteolin-7-*O*-glucoside, etc., were significantly induced by fungi (**Figures 5D–F**, **Supplementary Figures 6**, **7**, **Supplementary Table 2**). Interestingly, ginseng compounds associated with flavonoids were also observed upon *B. cinerea* B05.10 infection (Chen et al., 2022). The accumulation of kaempferol, quercetin, and luteolin was reduced, while the concentration of hesperetin increased in ginseng by *B. cinerea* (Chen et al., 2022). Further studies indicated that kaempferol, quercetin, and luteolin had antifungal activity toward *B. cinerea*, suggesting that the reduction of these compounds could be a strategy for fungal virulence (Chen et al., 2022). Here, the changes of ginseng compounds in the flavonoid pathway upon *C. panacicola* treatment may play a similar role.

### Inhibition analysis of ginseng compounds toward *C. panacicola*

**Figure 6:**
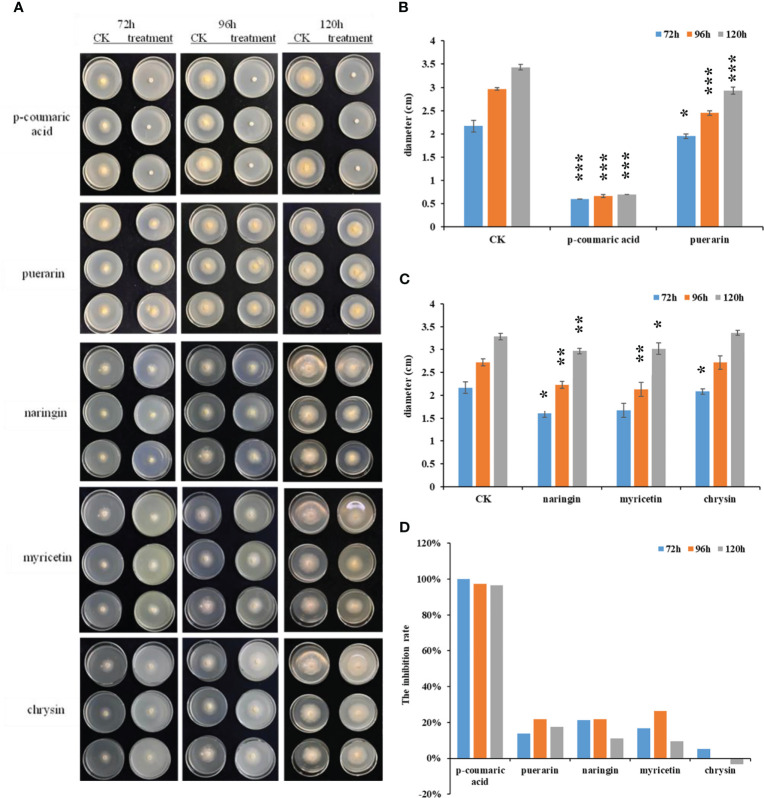
Antifungal activity of indicated chemicals toward *Colletotrichum panacicola* FSL. **(A)** Mycelial plugs of the *C. panacicola* FSL were inoculated on a potato dextrose agar (PDA) medium with different compounds including *p*-coumaric acid, puerarin, naringin, myricetin, and chrysin under the concentration of 1.0 mg/ml. The growth of the fungi was observed and photographed at 72, 96, and 120 h. **(B)** The colony diameters of *p*-coumaric acid and puerarin incubated fungus were determined at 72, 96, and 120 h. Asterisks indicate significant differences between untreated (CK) and chemicals treated (* *p* < 0.05, *** *p* < 0.001). **(C)** The colony diameters of naringin, myricetin, and chrysin incubated fungus were determined at 72 96, and 120 h. Asterisks indicate significant differences between untreated (CK) and chemicals treated (* *p* < 0.05; ** *p* < 0.01). **(D)** The relative inhibition rates of different compounds toward *C. panacicola* FSL were determined at 72, 96, and 120 h. All data represent means ± SD from at least three replicates.

To know if the altered compounds have antifungal activity toward *C. panacicola*, the fungi were incubated on potato dextrose agar (PDA) plates with several chemicals under the concentration of 1.0 mg/ml or 10.0 μmol/ml. These compounds include *p*-coumaric acid, puerarin, naringin, myricetin, and chrysin. The fungus without any chemicals was used as a control. The diameters and the inhibition rates were calculated at 48, 72, 96, or 120 h. As indicated in **Figure 6A**, under PDA concentration of 1.0 mg/ml, many compounds could suppress *C. panacicola* growth in the early stage. *p*-Coumaric acid could significantly highly suppress *C. panacicola* growth since the colony sizes were the smallest at 72, 96, and 120 h post-incubation (**Figures 6A, B**). The inhibition rates for *p*-coumaric acid were approximately 100% at 72 h, 97.2% at 96 h, and 96.5% at 120 h after incubation (**Figure 6D**). Puerarin, naringin, and myricetin could also significantly suppress the fungal growth at different times (72, 96, and 120 h) (**Figure 6**), but the inhibition rates were lower than those of *p*-coumaric acid. These results showed that *p*-coumaric acid, puerarin, naringin, and myricetin could inhibit *C. panacicola* growth, which further indicated that certain components in flavonoids have antifungal activity. However, chrysin could only inhibit fungal growth at the early stage (**Figures 6A, C, D**). Similarly, under PDA concentration of 10.0 μmol/ml, *p*-coumaric acid could completely inhibit the fungal growth at different times (**Supplementary Figures 9A, F**), while puerarin exhibited the second higher antifungal activity toward *C. panacicola* (**Supplementary Figures 9B, F**). The other three compounds could also inhibit fungal growth to a certain degree at the early stage (**Supplementary Figures 9C–F**). Considering the reduction of naringin (**Figure 5C**, **Supplementary Figure 6**, **Supplementary Table 2**), puerarin (**Supplementary Table 2**), and *p*-coumaric acid (**Supplementary Figure 8**) during ginseng–*C. panacicola* interaction at the early stage, *C. panacicola* very likely developed a strategy to reduce the biosynthesis of these antifungal metabolites.

Indeed, the expression of many genes in the metabolite biosynthesis pathway was differentially regulated upon *C. panacicola* interaction (**Supplementary Figures 6**, **7**). For example, the expressions of *Pg_S3366.3*, *Pg_S6977.4*, *Pg_S0360.15*, *Pg_S5731.2*, and *Pg_S6588.11* were increased upon *C. panacicola* infection, while the expression of *Pg_S4522.4* was decreased (**Supplementary Figures 6A, B**). Here, *Pg_S3366.3* encoded type III polyketide synthase B, *Pg_S6977.4* and *Pg_S6588.11* encoded enzymes belonging to EC:1.14.11.9 (flavanone 3-dioxygenase), *Pg_S0360.15* encoded isoflavone reductase, *Pg_S5731.2* encoded caffeoyl-CoA *O*-methyltransferase, while *Pg_S4522.4* encoded chalcone isomerase. These enzymes are involved in flavonoid biosynthesis. The induction and suppression of these genes suggested their role in the changes of flavonoids during ginseng–*C. panacicola* interaction. However, two genes in the isoflavonoid biosynthesis pathway, *Pg_S2855.12* and *Pg_S0785.34*, which encoded 2-hydroxyisoflavone dehydratase (EC:4.2.1.105), were differentially regulated upon *C. panacicola* infection (**Supplementary Figures 7A, B**). While the expression of *Pg_S2855.12* was decreased at 14 h, *Pg_S0785.34* was induced by *C. panacicola* at the same time. The increase and the decrease of the genes suggested their different roles in regulating isoflavonoid biosynthesis. Indeed, we observed that the accumulated metabolites in the isoflavonoid pathway were different upon *C. panacicola* infection (**Supplementary Figure 7C**).

## Discussion

The comparative transcriptome and metabolite analyses in this work provide molecular and chemical clues to understanding the complex interaction between garden ginseng and *C. panacicola* FSL. Here, *C. panacicola* strain FSL was originally isolated from ginseng leaves under forest conditions, which had higher virulence toward garden ginseng. Similarly, *C. panacicola* had also been reported to cause anthracnose disease in *P. ginseng* in Korea based on morphological, pathogenicity, cultural, and molecular studies (Choi et al., 2011). Recently, Liu et al. obtained 232 *Colletotrichum* isolates from 12 main ginseng-producing areas in Northeast China (Liu et al., 2020). Based on morphological characteristics and sequence analyses of specific domains in the fungal genome, 135 out of 232 isolates belonged to *C. panacicola*, while the other isolates were grouped with *Colletotrichum lineola* (Liu et al., 2020). Further work revealed the isolates of *C. panacicola* presented in all three provinces (Jilin, Liaoning, and Heilongjiang) in Northeast China, suggesting that *C. panacicola* was the widely distributed *Colletotrichum* species in China (Liu et al., 2020). Inoculations of ginseng showed that the isolates of *C. panacicola* could infect both leaves and stems with higher aggressiveness (Liu et al., 2020). In addition, Cui also isolated a virulent strain of *C. panacicola* from the wild ginseng in Jilin Province (Cui et al., 2020). All reports indicated that *C. panacicola* caused garden ginseng anthracnose disease.

Illumina-based RNA-seq of ginseng leaves revealed that plant transcripts changed upon *C. panacicola* FSL infection at different times. However, more than half of the transcripts in ginseng showed decreased expression at 24 h, indicating that ginseng defense responses were suppressed by *C. panacicola* from 14 to 24 h. This is consistent with our previous report that *B. cinerea* B05.10 infection downregulated approximately 60% of SSTFs in ginseng at 14 h, suggesting that these genes play a special role in plant–fungus interaction (Chen et al., 2022). Therefore, *B. cinerea* B05.10 promotes disease development in *P. ginseng* by suppressing many defense-related genes’ expression at the early stage. Here, *C. panacicola* FSL may use a similar strategy. Other reports also indicated the transcript changes in red skin root-affected ginseng fibrous roots (Gong et al., 2020). The fibrous roots of red skin root-affected ginseng showed 1,856 upregulated genes and 1,581 downregulated genes when compared with the healthy ginseng roots (Gong et al., 2020). Thus, *P. ginseng* changed its transcripts upon different stresses. Here, the differentially expressed genes in garden ginseng upon *C. panacicola* infection suggest their potential roles in defense. Indeed, we observed that the transcripts and the accumulation of antifungal compounds in the flavonoid pathway changed upon *C. panacicola* treatment.

Previous works indicate that plants had various mixtures and high amounts of antifungal compounds (Whitehead and Bowers, 2013), some of which provide the plants with a certain degree of basic resistance against pests (Schijlen et al., 2004; Treutter, 2005). For example, camalexin is a major phytoalexin in *Arabidopsis thaliana*, which is induced by a great number of biotrophic and necrotrophic plant pathogens (Glazebrook, 2005). Camalexin biosynthetic capacity is observed in both leaf and root upon infection with the *Pythium sylvaticum* (Bednarek et al., 2005). High camalexin concentrations were observed at the infection site by *Alternaria alternata* or *B. cinerea* (Kliebenstein et al., 2005; Ahuja et al., 2012). Camalexin can also be induced by PAMPs. For example, in oomycete-derived necrosis and ethylene-induced peptide 1 (Nep1)-like proteins, bacteria-derived peptidoglycans were involved in camalexin accumulation (Qutob et al., 2006; Gust et al., 2007). In addition, the changes in flavonoids have also been identified from various plant species to date (Li et al., 2018; Sudheeran et al., 2019; Yu et al., 2018). These compounds are not only involved in the innate defense response but also induced in the host’s response to pathogen attack (Heale, 1992; Jeandet et al., 1995). Metabolite analysis of *P. ginseng* indicates that in many of the secondary metabolites in the flavone and flavonol biosynthesis pathway, isoflavonoid biosynthesis is influenced by *B. cinerea* B05.10 (Chen et al., 2022). It also indicated the red mango fruit had more anthocyanin and flavonols than the green mango fruit, which had antifungal activity against the fungus *Colletotrichum gloeosporioides* (Sudheeran et al., 2020). Previous reports have also shown that quercetin and cyanidin aglycones could inhibit *C. gloeosporioides* hyphal growth and conidial germination (Puupponen-Pimiä et al., 2001; Ramos et al., 2006; Mikulic-Petkovsek et al., 2013). Since certain flavonoids could inhibit *C. panacicola* growth; here, the alteration of ginseng flavonoids at the early stage would be a candidate strategy for *C. panacicola* to cause disease development.

## Conclusion

The comparative transcriptome analysis in this work provides molecular clues to understanding the mechanism of ginseng susceptibility toward *C. panacicola*. Illumina-based RNA-seq of ginseng leaves revealed that plant transcripts changed upon *C. panacicola* infection at different times. More than half of the transcripts in ginseng showed decreased expression at 24 h, indicating that ginseng defense responses were suppressed by *C. panacicola* from 14 to 24 h. In particular, the transcripts and the accumulation of certain antifungal compounds in the flavonoid pathway changed upon *C. panacicola* treatment. We hope this finding can help in understanding the pathogenicity of *C. panacicola* in promoting *P. ginseng* anthracnose disease development.

## Materials and methods

### Ginseng, fungal materials, and treatments

Two-year-old garden ginseng was grown in a pathogen-free chamber. *C. panacicola* strain FSL was isolated from ginseng leaves in the Fusong forest, Jilin Province, and was grown on a PDA plate. Infection of *C. panacicola* on ginseng leaves was performed (Chen et al., 2022). For ginseng mRNA sequencing and gene qPCR analysis, the spores were spray incubated on healthy, undetached ginseng at different times. For mRNA sequencing, ginseng leaves were harvested at 14 h (Cp14h) and 24 h (Cp24h). For control, ginseng leaves were only incubated with ddH_2_O. For qPCR analysis, the leaves were harvested at 14, 24, and 48 h. Three independent biological replicates were performed. All samples were frozen at −80°C until use. The primers for qPCR are shown in **Supplementary Table 3**.

### Library construction, RNA sequencing, and mapping fragments to the genome and quantification of gene level

Total RNA extraction, purification, monitoring, cDNA library construction and sequencing, raw data cleaning, and analyzing were performed as previously indicated (Chen et al., 2022). Reference genome information was observed from the ginseng genome database website (http://ginsengdb.snu.ac.kr/). An index of the reference genome was built, and paired-end clean reads were aligned by using the HISAT package, while the FPKM of each gene was finally calculated (Chen et al., 2022).

### Identification and Gene Ontology and Kyoto Encyclopedia of Genes and Genomes enrichment analyses of differentially expressed genes

Differential expression analysis of genes in all samples (CK1-3, Cp14h1-3, and Cp24h1-3) was performed as previously described (Chen et al., 2022). The differentially expressed genes were identified with log2 (fold change) >1 or log2 (fold change) <−1 and with statistical significance (*p*-value < 0.05) using the R package.

GO enrichment and KEGG analyses of differentially expressed genes were then performed (Chen et al., 2022).

### Determination of individual flavonoids by UPLC-ESI-MS/MS

The flavonoids were extracted and measured according to the methods described previously, with minor modifications (Mi et al., 2022). Approximately 100 mg of ginseng leaves was extracted two times with 300 µl of methanol:acetonitrile:water (2:2:1, v/v) in an ultrasonic sonicator for 0.5 h at 4°C. The sample was incubated at −20°C for 1 h for protein deposition. After centrifugation (14,000 rpm, 20 min, 4°C), the supernatants were collected, freeze-dried, and stored at −80°C. Then, the supernatants were filtered, collected, and separated on a Waters C18 column (2.1 mm × 150 mm, 1.7 µm) with a flow rate of 400 µl/min. For the separation, solvent A (water with 0.1% formic acid) and solvent B (acetonitrile with 0.1% formic acid) were used under the gradient, as follows: 0–3.0 min, 5%–20% B; 3.0–4.3 min, 20% B; 4.3–9.0 min, 20%–45% B; 9.0–11.0 min, 45%–98% B; 11.0–13.0 min, 98% B; 13.0–13.1 min, 98%–5% B; and 13.1–15.0 min, 5% B. The auto-sampler temperature was set to 4°C, and 2 µl of the extraction was injected for analysis.

Mass spectrometric detection was carried out using an ABSCIEX QTRAP 6500 mass spectrometer coupled with an electrospray ionization (ESI) source under both negative (−) and positive (+) ion modes. The multiple reaction monitoring (MRM) scan mode was used to quantify the individual flavonoid compounds. All samples were equally mixed for quality control (QC) to indicate stability and repeatability during the experiments. Relative standard deviation (RSD) in QC samples was calculated, and the RSD% below 15% was considered and further analyzed. Metabolites ferulic acid, *p*-coumaric acid, glycetein, glycitin, daidzein, puerarin, formononetin, daidzin, biochanin A, genistin, genistein, apigenin, myricetin, quercetin, isorhamnetin, luteolin, kaempferol, kaempferide, sakuranetin, quercetin 3-glucoside, liquiritigenin, catechin, dihydrokaempferol, quercitrin, eriodictyol, gallocatechin, epicatechin, naringenin, rutin, epigallocatechin, isoliquiritigenin, chrysin, luteolin-7-*O*-glucoside, naringin, taxifolin, isovitexin/vitexin, and butin were used as standards for sample quantification (Sigma Chemical Company, St. Louis, MO, USA; **Supplementary Table 1**).

### Antifungal function analysis of flavonoids

The antifungal activity of flavonoids toward *C. panacicola* was assessed by measuring the mycelial plug growth (Chen et al., 2022). The indicated flavonoids (98%) were first dissolved in methanol or ethanol and then pre-mixed with a sterile melting PDA medium to obtain the final concentration (1.0 mg/ml or 10.0 μmol/ml). The incubation of fungi and the measurement of mycelial growth were performed by Chen et al. (2022). Each experiment was performed in three replicates.

## Data availability statement

The original contributions presented in the study are publicly available. This data can be found here: https://www.ncbi.nlm.nih.gov/geo/query/acc.cgi?acc=GSE226837.

## Author contributions

SL designed the research plan and wrote the article, JX, NL, JH, JS, and SL performed the research. All authors contributed to the article and approved the submitted version.

## Supplementary material

Supplementary Figure 1
*C. panacicola* FSL infection phenotype on 2-years old ginseng leaves at 14 hours, 24 hours and 48 hours, respectively.

Supplementary Figure 2 GO/KEGG analysis of significantly differentially expressed genes in ginseng upon *C. panacicola* FSL treatment at 14 hours.

Supplementary Figure 3 GO/KEGG analysis of significantly differentially expressed genes in ginseng upon *C. panacicola* FSL treatment at 24 hours.

Supplementary Figure 4 GO enrichment analysis of up- and down- regulated SSTF genes in ginseng upon *C. panacicola* FSL treatment at 14 hours and 24 hours, respectively.

Supplementary Figure 5 XIC map of standard mixtures and QC RSD percentage of ginseng samples in this work. QC RSD% < 15% was considered.

Supplementary Figure 6 Differentially accumulated chemicals and genes in flavonoid biosynthesis pathway upon *C. panacicola* FSL treatment.

Supplementary Figure 7 Differentially accumulated chemicals and genes in isoflavonoid biosynthesis pathway upon *C. panacicola* FSL treatment.

Supplementary Figure 8 Differentially accumulated chemicals in phenylpropanoid biosynthesis pathway upon *C. panacicola* FSL treatment.

Supplementary Figure 9 Anti-fungi activity of indicated chemicals towards *C. panacicola* FSL under the concentration of 10.0 umol/mL.

Supplementary File 1 Total validated high-quality reads were obtained from all libraries.

Supplementary File 2 XIC maps of selected compounds observed in untreated control (CK) or *C. panacicola* FSL treated ginseng leaves (CpFSL).
