# Supplementary material for: Transcriptome and metabolite analyses indicated the underlying molecular responses of Asian ginseng (Panax ginseng) toward Colletotrichum panacicola infection
Source: Front Plant Sci. 2023 Jul 10;14:1182685. doi: 10.3389/fpls.2023.1182685 (PMC10365858; doi:10.3389/fpls.2023.1182685)
Supplement: Supplementary Figure 1 — C. panacicola FSL infection phenotype on 2-years old ginseng leaves at 14 hours, 24 hours and 48 hours, respectively. [file DataSheet_1.pdf]

A

14h

B

24h

C

48h

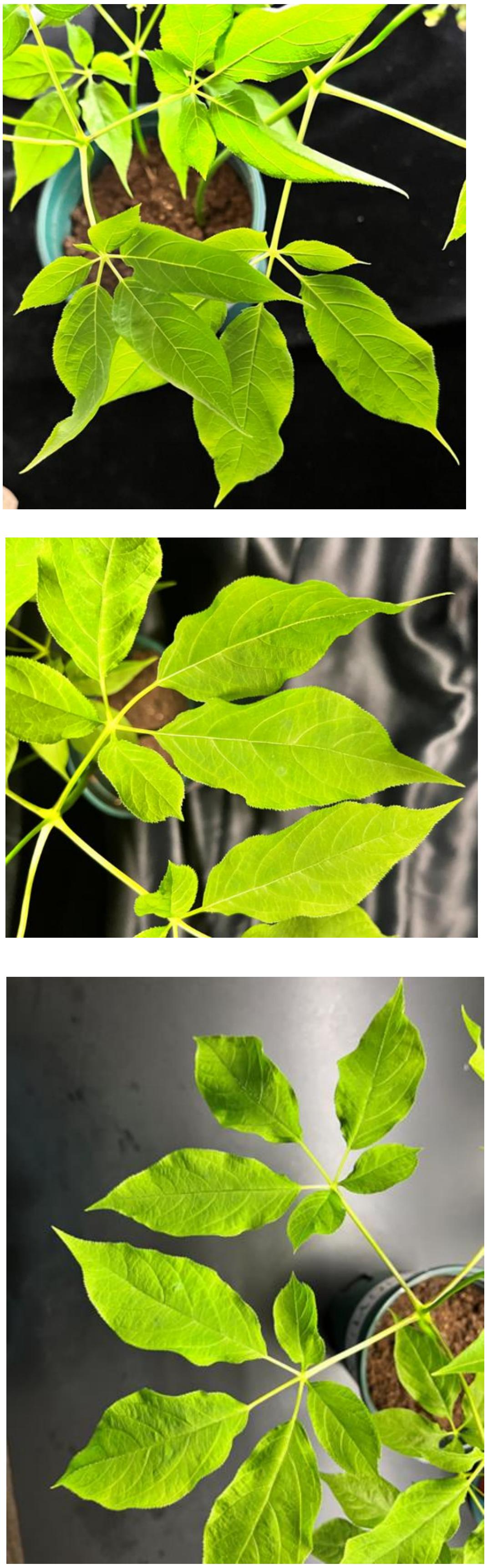

Figure S1.

A

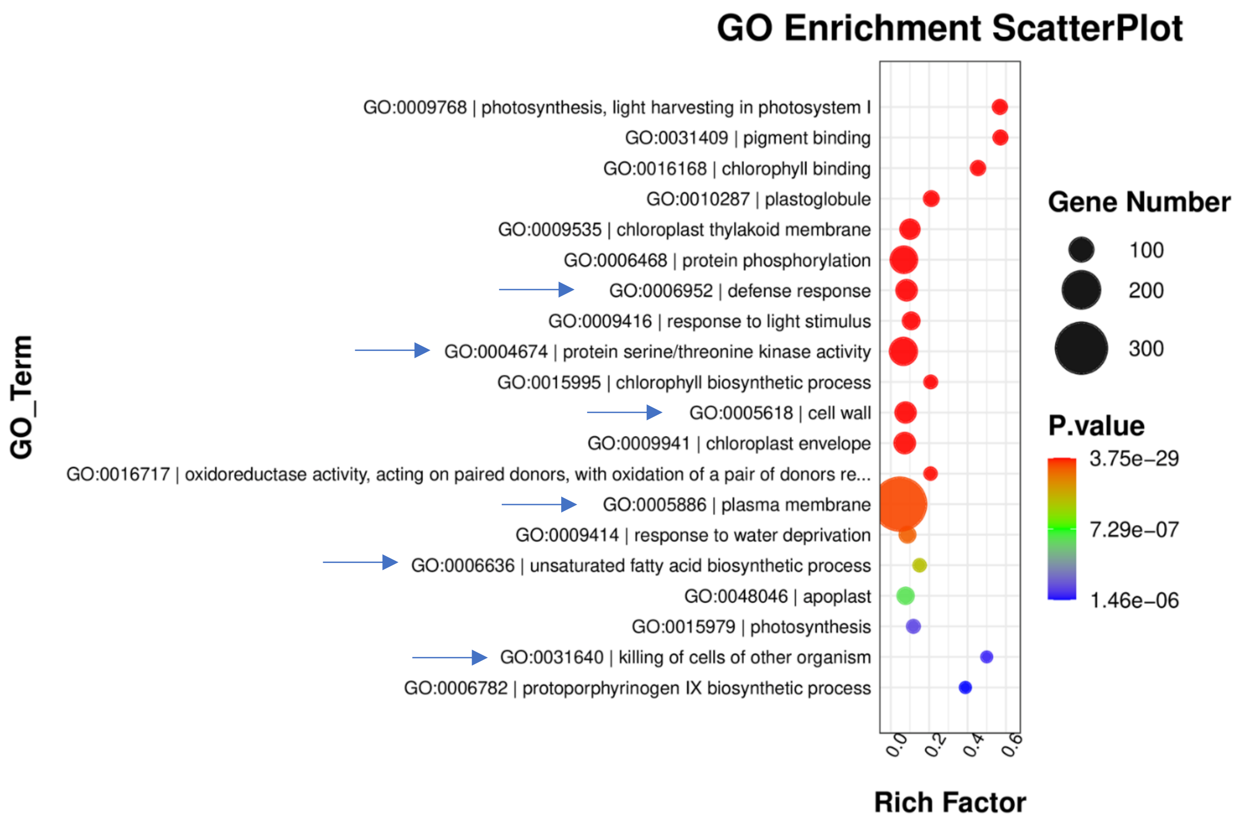

GO Enrichment ScatterPlot
GO_Term
GO:0009768 | photosynthesis, light harvesting in photosystem I
GO:0031409 | pigment binding
GO:0016168 | chlorophyll binding
GO:0010287 | plastoglobule
GO:0009535 | chloroplast thylakoid membrane
GO:0006468 | protein phosphorylation
GO:0006952 | defense response
GO:0009416 | response to light stimulus
GO:0004674 | protein serine/threonine kinase activity
GO:0015995 | chlorophyll biosynthetic process
GO:0005618 | cell wall
GO:0009941 | chloroplast envelope
GO:0016717 | oxidoreductase activity, acting on paired donors, with oxidation of a pair of donors re...
GO:0005886 | plasma membrane
GO:0009414 | response to water deprivation
GO:0006636 | unsaturated fatty acid biosynthetic process
GO:0048046 | apoplast
GO:0015979 | photosynthesis
GO:0031640 | killing of cells of other organism
GO:0006782 | protoporphyrinogen IX biosynthetic process
0.0
0.2
0.4
0.6
Rich Factor
Gene Number
100
200
300
P.value
3.75e−29
7.29e−07
1.46e−06

B

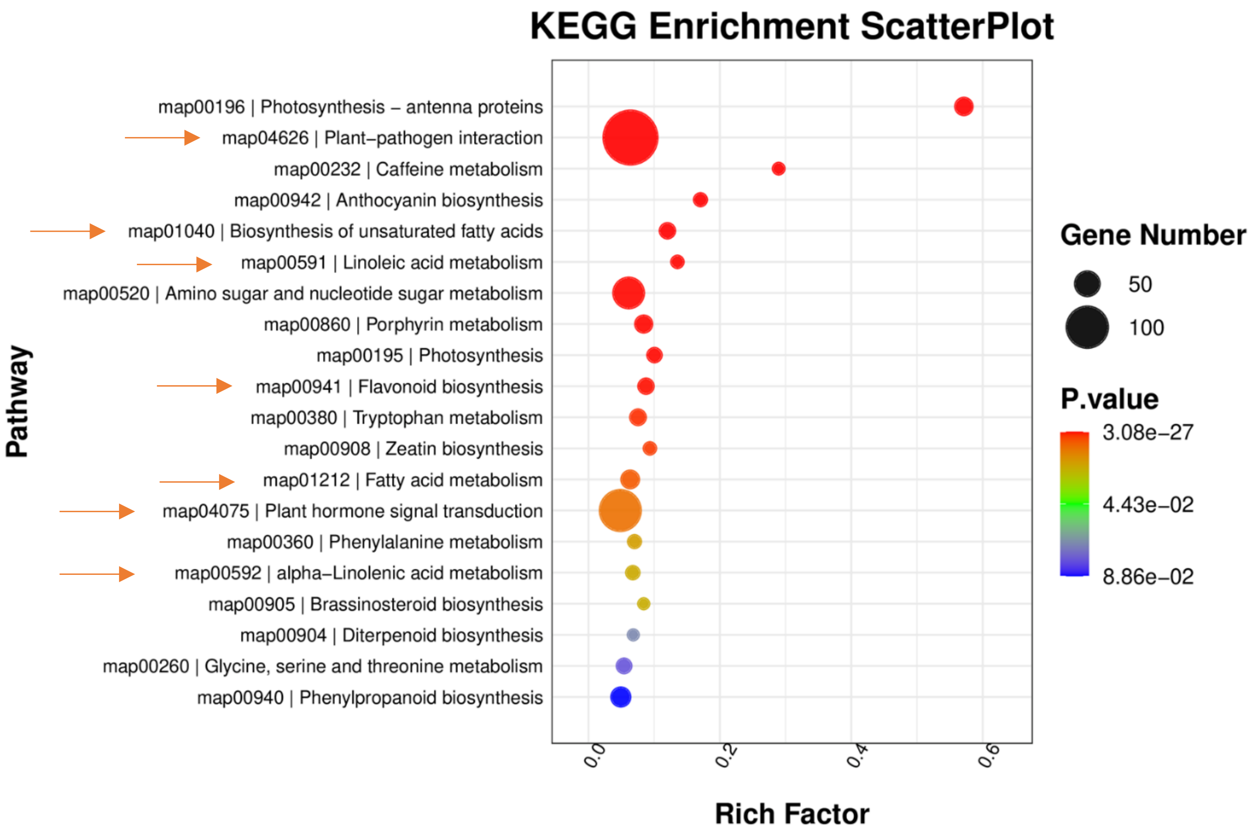
KEGG Enrichment ScatterPlot
map00196 | Photosynthesis – antenna proteins
map04626 | Plant–pathogen interaction
map00232 | Caffeine metabolism
map00942 | Anthocyanin biosynthesis
map01040 | Biosynthesis of unsaturated fatty acids
map00591 | Linoleic acid metabolism
map00520 | Amino sugar and nucleotide sugar metabolism
map00860 | Porphyrin metabolism
map00195 | Photosynthesis
map00941 | Flavonoid biosynthesis
map00380 | Tryptophan metabolism
map00908 | Zeatin biosynthesis
map01212 | Fatty acid metabolism
map04075 | Plant hormone signal transduction
map00360 | Phenylalanine metabolism
map00592 | alpha–Linolenic acid metabolism
map00905 | Brassinosteroid biosynthesis
map00904 | Diterpenoid biosynthesis
map00260 | Glycine, serine and threonine metabolism
map00940 | Phenylpropanoid biosynthesis
Pathway
0.0
0.2
0.4
0.6
Rich Factor
Gene Number
50
100
P.value
3.08e–27
4.43e–02
8.86e–02

Figure S2.

A

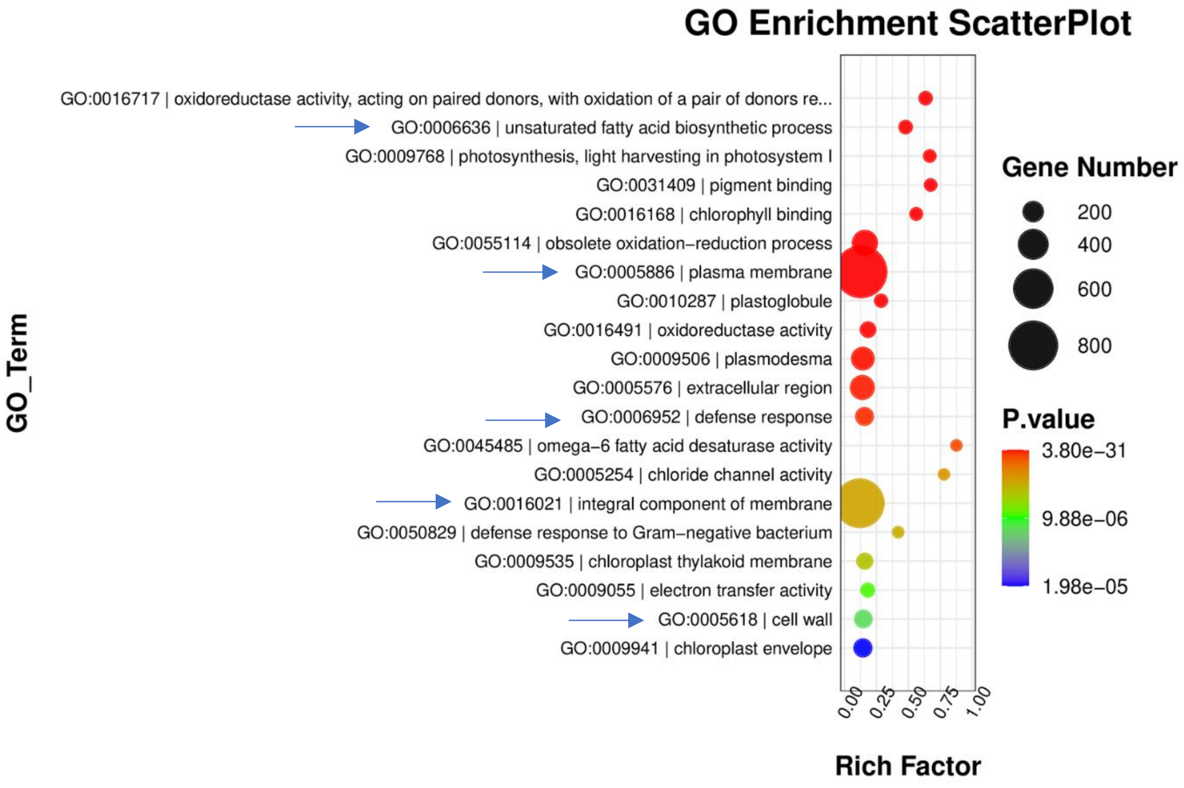

GO Enrichment ScatterPlot
GO_Term
GO:0016717 | oxidoreductase activity, acting on paired donors, with oxidation of a pair of donors re...
GO:0006636 | unsaturated fatty acid biosynthetic process
GO:0009768 | photosynthesis, light harvesting in photosystem I
GO:0031409 | pigment binding
GO:0016168 | chlorophyll binding
GO:0055114 | obsolete oxidation−reduction process
GO:0005886 | plasma membrane
GO:0010287 | plastoglobule
GO:0016491 | oxidoreductase activity
GO:0009506 | plasmodesma
GO:0005576 | extracellular region
GO:0006952 | defense response
GO:0045485 | omega−6 fatty acid desaturase activity
GO:0005254 | chloride channel activity
GO:0016021 | integral component of membrane
GO:0050829 | defense response to Gram−negative bacterium
GO:0009535 | chloroplast thylakoid membrane
GO:0009055 | electron transfer activity
GO:0005618 | cell wall
GO:0009941 | chloroplast envelope
0.00
0.25
0.50
0.75
1.00
Rich Factor
Gene Number
200
400
600
800
P.value
3.80e−31
9.88e−06
1.98e−05

B

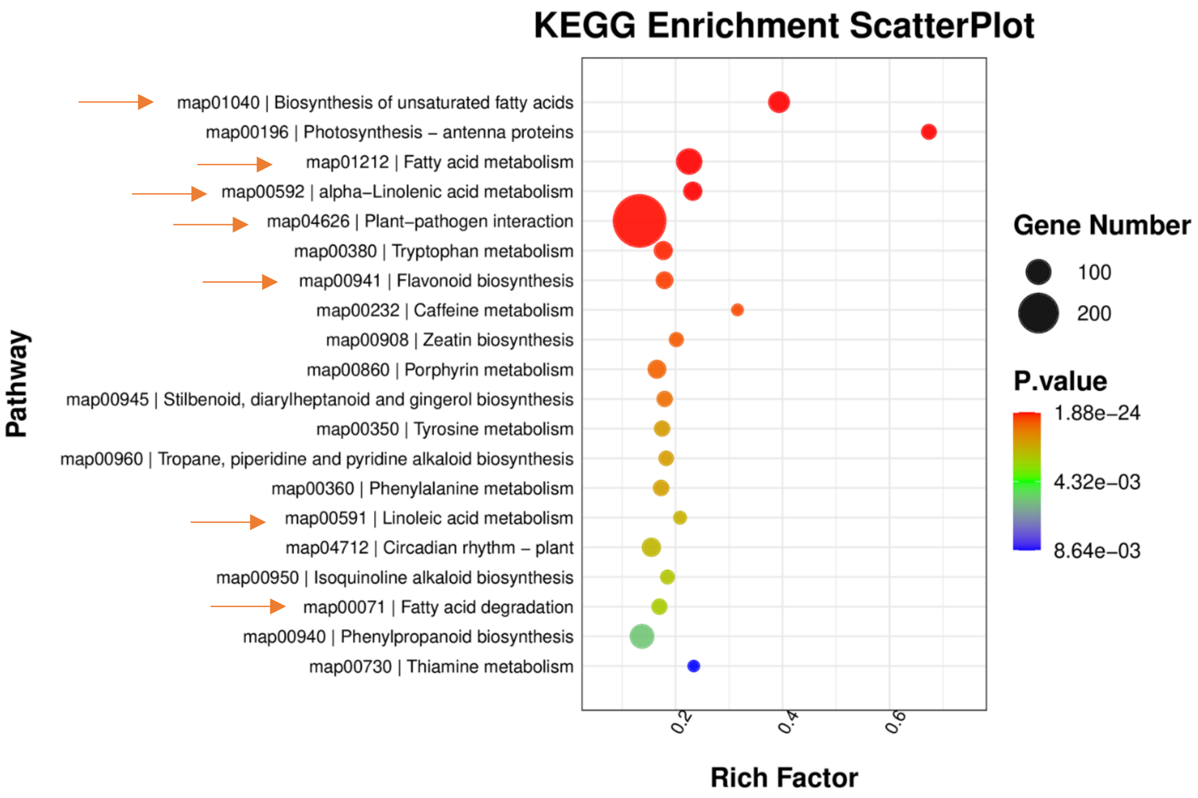
KEGG Enrichment ScatterPlot
map01040 | Biosynthesis of unsaturated fatty acids
map00196 | Photosynthesis – antenna proteins
map01212 | Fatty acid metabolism
map00592 | alpha–Linolenic acid metabolism
map04626 | Plant–pathogen interaction
map00380 | Tryptophan metabolism
map00941 | Flavonoid biosynthesis
map00232 | Caffeine metabolism
map00908 | Zeatin biosynthesis
map00860 | Porphyrin metabolism
map00945 | Stilbenoid, diarylheptanoid and gingerol biosynthesis
map00350 | Tyrosine metabolism
map00960 | Tropane, piperidine and pyridine alkaloid biosynthesis
map00360 | Phenylalanine metabolism
map00591 | Linoleic acid metabolism
map04712 | Circadian rhythm – plant
map00950 | Isoquinoline alkaloid biosynthesis
map00071 | Fatty acid degradation
map00940 | Phenylpropanoid biosynthesis
map00730 | Thiamine metabolism
Pathway
0.2
0.4
0.6
Rich Factor
Gene Number
100
200
P.value
1.88e–24
4.32e–03
8.64e–03

Figure S3.

A

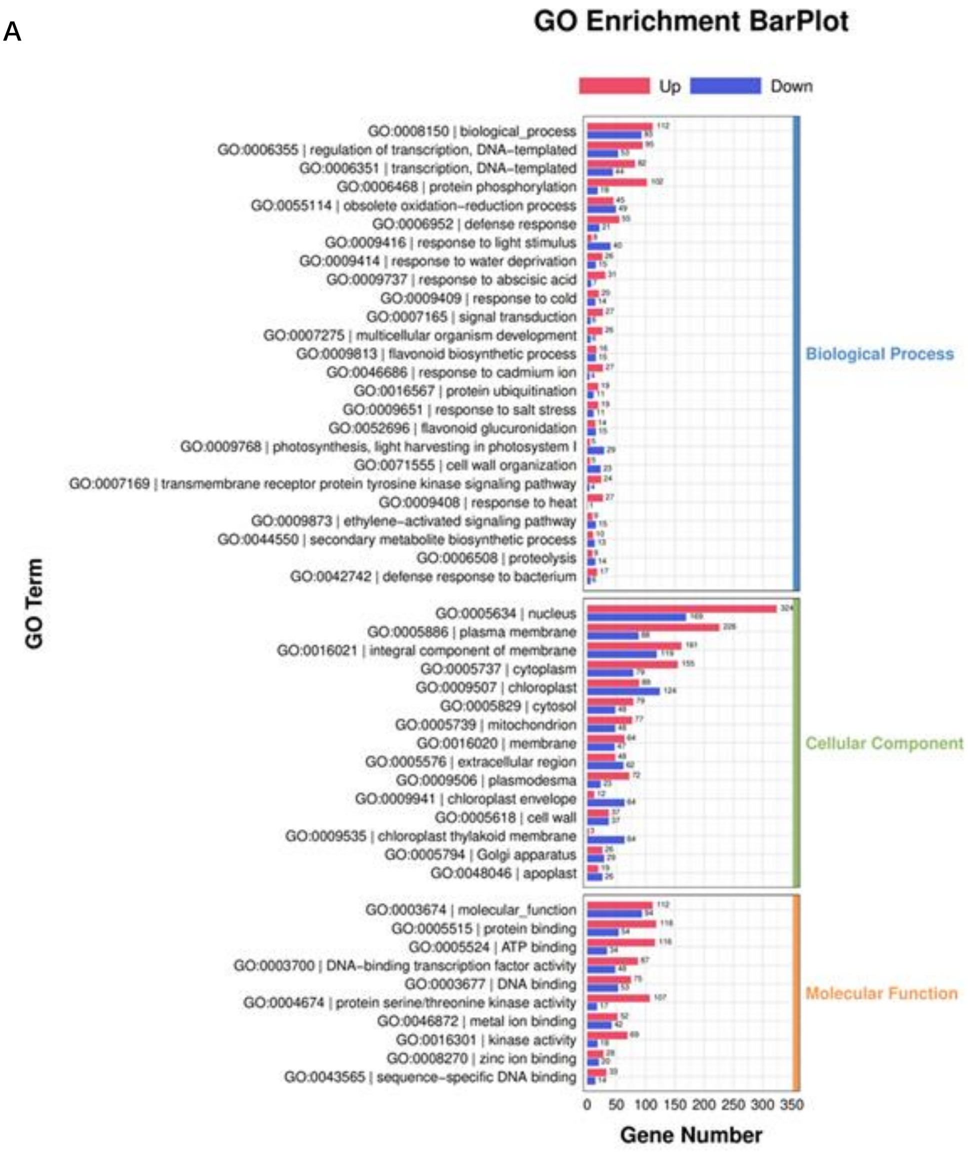

GO Enrichment BarPlot
Up
Down
GO Term
GO:0008150 | biological_process
GO:0006355 | regulation of transcription, DNA−templated
GO:0006351 | transcription, DNA−templated
GO:0006468 | protein phosphorylation
GO:0055114 | obsolete oxidation−reduction process
GO:0006952 | defense response
GO:0009416 | response to light stimulus
GO:0009414 | response to water deprivation
GO:0009737 | response to abscisic acid
GO:0009409 | response to cold
GO:0007165 | signal transduction
GO:0007275 | multicellular organism development
GO:0009813 | flavonoid biosynthetic process
GO:0046686 | response to cadmium ion
GO:0016567 | protein ubiquitination
GO:0009651 | response to salt stress
GO:0052696 | flavonoid glucuronidation
GO:0009768 | photosynthesis, light harvesting in photosystem I
GO:0071555 | cell wall organization
GO:0007169 | transmembrane receptor protein tyrosine kinase signaling pathway
GO:0009408 | response to heat
GO:0009873 | ethylene−activated signaling pathway
GO:0044550 | secondary metabolite biosynthetic process
GO:0006508 | proteolysis
GO:0042742 | defense response to bacterium
Biological Process
GO:0005634 | nucleus
GO:0005886 | plasma membrane
GO:0016021 | integral component of membrane
GO:0005737 | cytoplasm
GO:0009507 | chloroplast
GO:0005829 | cytosol
GO:0005739 | mitochondrion
GO:0016020 | membrane
GO:0005576 | extracellular region
GO:0009506 | plasmodesma
GO:0009941 | chloroplast envelope
GO:0005618 | cell wall
GO:0009535 | chloroplast thylakoid membrane
GO:0005794 | Golgi apparatus
GO:0048046 | apoplast
Cellular Component
GO:0003674 | molecular_function
GO:0005515 | protein binding
GO:0005524 | ATP binding
GO:0003700 | DNA-binding transcription factor activity
GO:0003677 | DNA binding
GO:0004674 | protein serine/threonine kinase activity
GO:0046872 | metal ion binding
GO:0016301 | kinase activity
GO:0008270 | zinc ion binding
GO:0043565 | sequence−specific DNA binding
Molecular Function
0 50 100 150 200 250 300 350
Gene Number

B

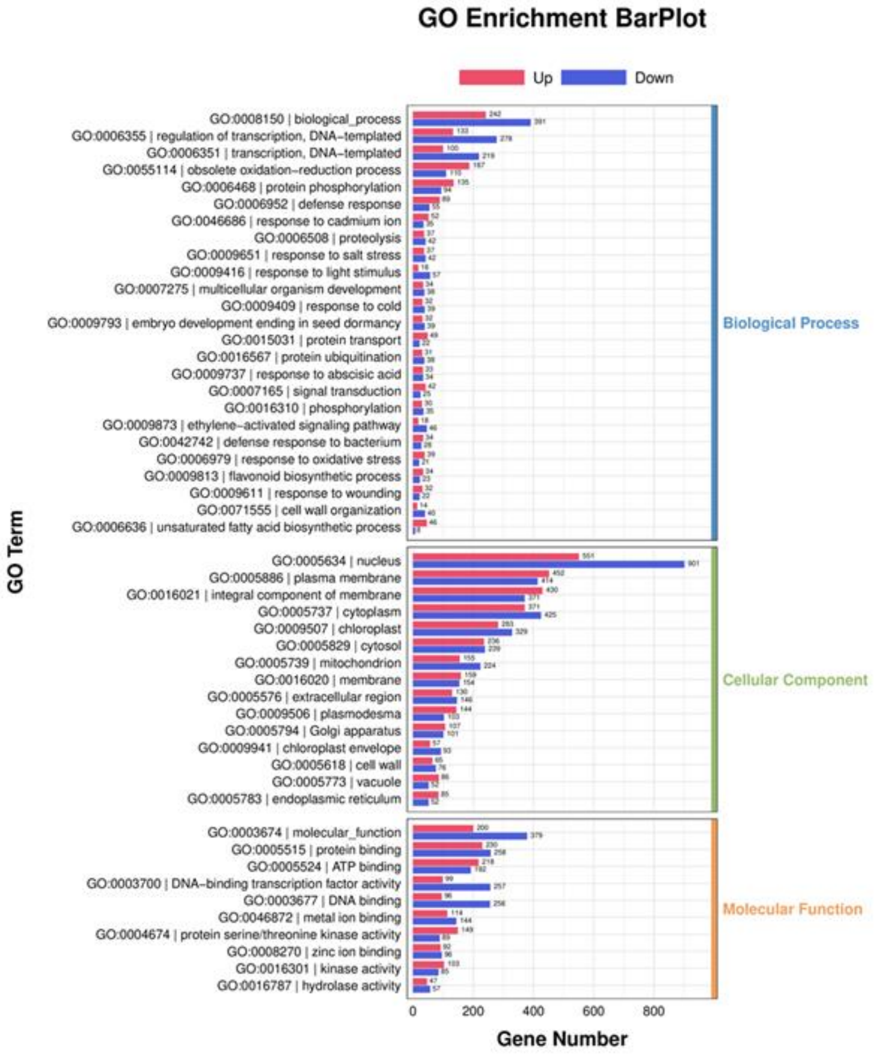

GO Enrichment BarPlot
Up
Down
GO:0008150 | biological_process
GO:0006355 | regulation of transcription, DNA−templated
GO:0006351 | transcription, DNA−templated
GO:0055114 | obsolete oxidation−reduction process
GO:0006468 | protein phosphorylation
GO:0006952 | defense response
GO:0046686 | response to cadmium ion
GO:0006508 | proteolysis
GO:0009651 | response to salt stress
GO:0009416 | response to light stimulus
GO:0007275 | multicellular organism development
GO:0009409 | response to cold
GO:0009793 | embryo development ending in seed dormancy
GO:0015031 | protein transport
GO:0016567 | protein ubiquitination
GO:0009737 | response to abscisic acid
GO:0007165 | signal transduction
GO:0016310 | phosphorylation
GO:0009873 | ethylene−activated signaling pathway
GO:0042742 | defense response to bacterium
GO:0006979 | response to oxidative stress
GO:0009813 | flavonoid biosynthetic process
GO:0009611 | response to wounding
GO:0071555 | cell wall organization
GO:0006636 | unsaturated fatty acid biosynthetic process
GO:0005634 | nucleus
GO:0005886 | plasma membrane
GO:0016021 | integral component of membrane
GO:0005737 | cytoplasm
GO:0009507 | chloroplast
GO:0005829 | cytosol
GO:0005739 | mitochondrion
GO:0016020 | membrane
GO:0005576 | extracellular region
GO:0009506 | plasmodesma
GO:0005794 | Golgi apparatus
GO:0009941 | chloroplast envelope
GO:0005618 | cell wall
GO:0005773 | vacuole
GO:0005783 | endoplasmic reticulum
GO:0003674 | molecular_function
GO:0005515 | protein binding
GO:0005524 | ATP binding
GO:0003700 | DNA−binding transcription factor activity
GO:0003677 | DNA binding
GO:0046872 | metal ion binding
GO:0004674 | protein serine/threonine kinase activity
GO:0008270 | zinc ion binding
GO:0016301 | kinase activity
GO:0016787 | hydrolase activity
Biological Process
Cellular Component
Molecular Function
GO Term
0
200
400
600
800
Gene Number

Figure S4.

A

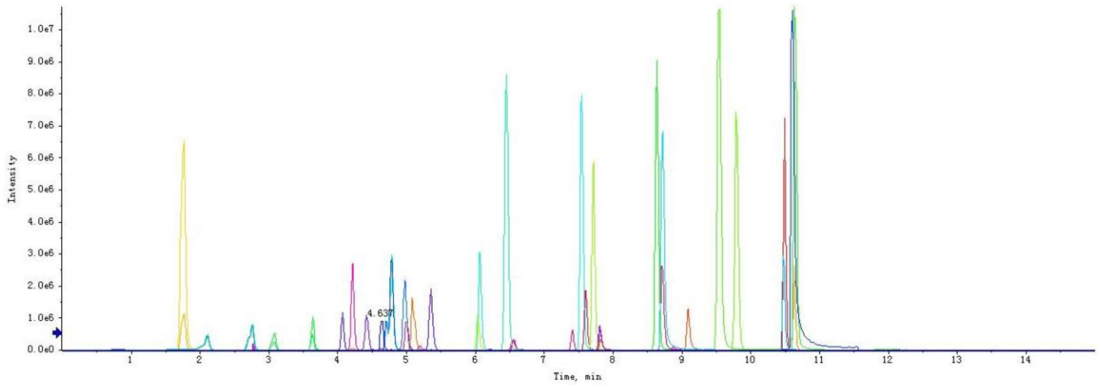

Intensity
1.0e7
9.0e6
8.0e6
7.0e6
6.0e6
5.0e6
4.0e6
3.0e6
2.0e6
1.0e6
0.0e0
4.637
1
2
3
4
5
6
7
8
9
10
11
12
13
14
Time, min

B

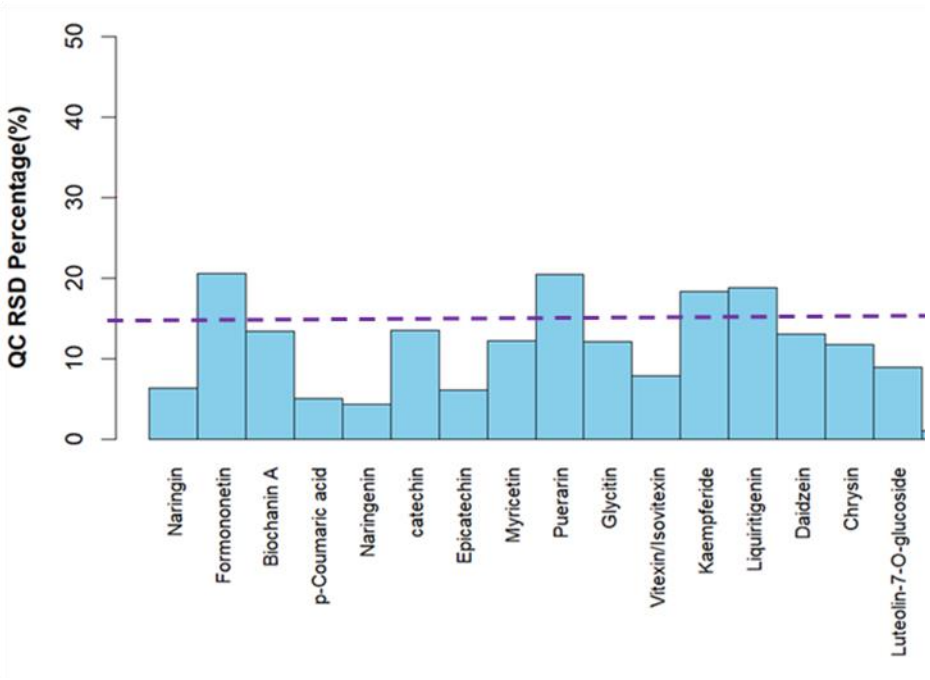

QC RSD Percentage(%)
50
40
30
20
10
0
Naringin
Formononetin
Biochanin A
p-Coumaric acid
Naringenin
catechin
Epicatechin
Myricetin
Puerarin
Glycitin
Vitexin/Isovitexin
Kaempferide
Liquiritigenin
Daidzein
Chrysin
Luteolin-7-O-glucoside

Figure S5.

A

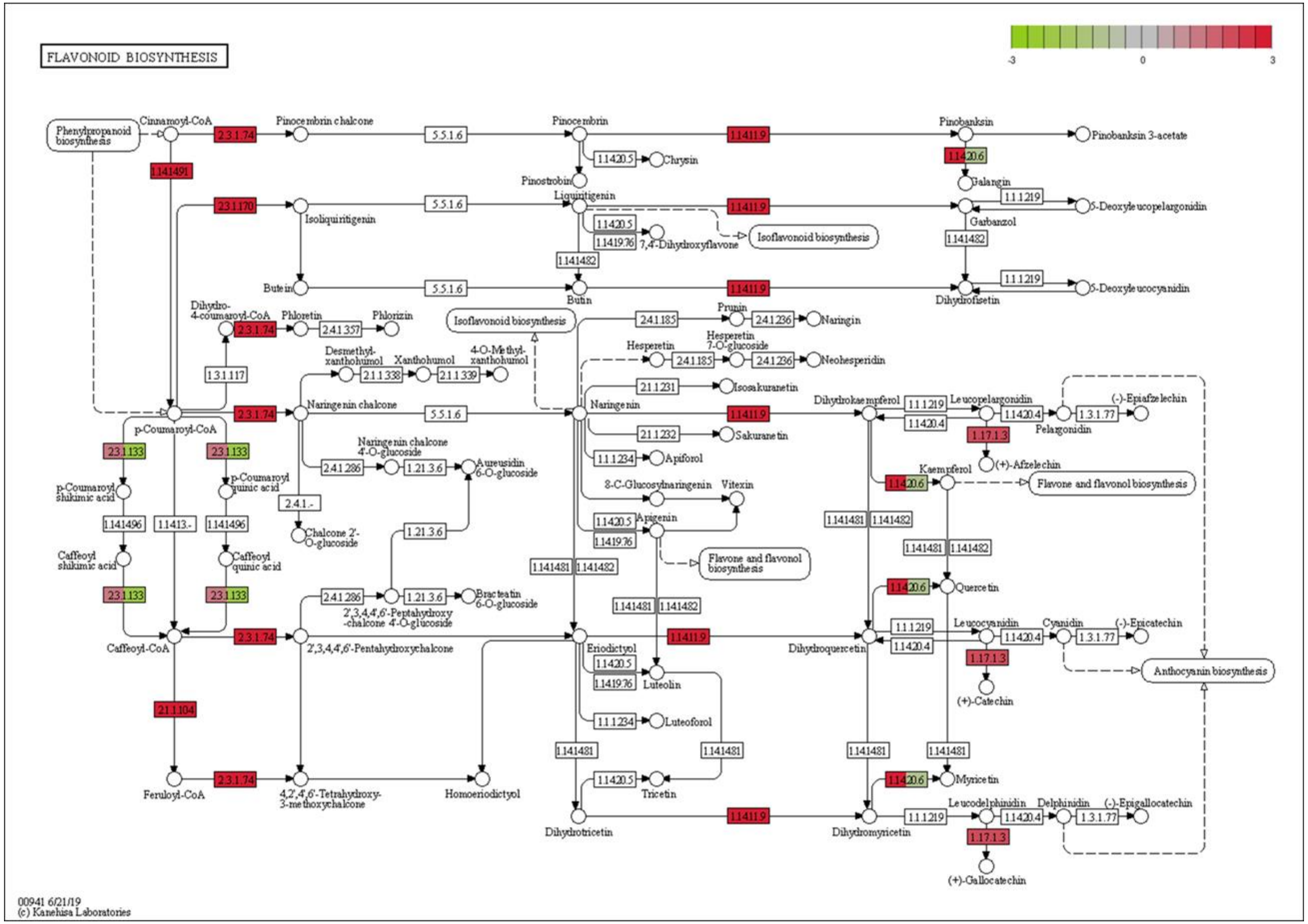
FLAVONOID BIOSYNTHESIS
-3
0
3
Phenylpropanoid biosynthesis
Cinnamoyl-CoA
2.3.1.74
Pinocembrin chalcone
5.5.1.6
Pinocembrin
1.14.11.9
Pinobanksin
Pinobanksin 3-acetate
1.14.20.6
Galangin
1.14.20.5
Chrysin
Pinostrobin
1.14.14.91
2.3.1.170
Isoliquiritigenin
Liquiritigenin
1.1.1.219
5-Deoxyleucopelargonidin
Garbanzol
1.14.19.76
7,4'-Dihydroxyflavone
Isoflavonoid biosynthesis
1.14.14.82
Butein
Butin
Dihydrofisetin
5-Deoxyleucocyanidin
Dihydro-4-coumaroyl-CoA
Phloretin
2.4.1.357
Phlorizin
2.4.1.185
Prunin
2.4.1.236
Naringin
Hesperetin
Hesperetin 7-O-glucoside
Neohesperidin
1.3.1.117
Desmethylxanthohumol
2.1.1.338
Xanthohumol
2.1.1.339
4-O-Methylxanthohumol
2.1.1.231
Isosakuranetin
Naringenin chalcone
Naringenin
Dihydrokaempferol
1.14.20.4
Leucopelargonidin
Pelargonidin
1.3.1.77
(-)-Epiafzelechin
1.17.1.3
(+)-Afzelechin
p-Coumaroyl-CoA
2.3.1.133
2.1.1.232
Sakuranetin
Naringenin chalcone 4'-O-glucoside
2.4.1.286
1.21.3.6
Aureusidin 6-O-glucoside
1.1.1.234
Apiforol
Kaempferol
Flavone and flavonol biosynthesis
p-Coumaroyl shikimic acid
p-Coumaroyl quinic acid
2.4.1.-
8-C-Glucosylnaringenin
Vitexin
1.14.13.-
1.14.14.96
Chalcone 2'-O-glucoside
Apigenin
1.14.14.81
Caffeoyl shikimic acid
Caffeoyl quinic acid
Quercetin
Bracteatin 6-O-glucoside
2',3,4,4',6'-Peptahydroxychalcone 4'-O-glucoside
Caffeoyl-CoA
2',3,4,4',6'-Pentahydroxychalcone
Eriodictyol
Dihydroquercetin
Leucocyanidin
Cyanidin
(-)-Epicatechin
Anthocyanin biosynthesis
Luteolin
(+)-Catechin
2.1.1.104
Luteoforol
Feruloyl-CoA
4,2',4',6'-Tetrahydroxy-3-methoxychalcone
Homoeriodictyol
Tricetin
Myricetin
Dihydrotricetin
Dihydromyricetin
Leucodelphinidin
Delphinidin
(-)-Epigallocatechin
(+)-Gallocatechin
00941 6/21/19
(c) Kanehisa Laboratories

B

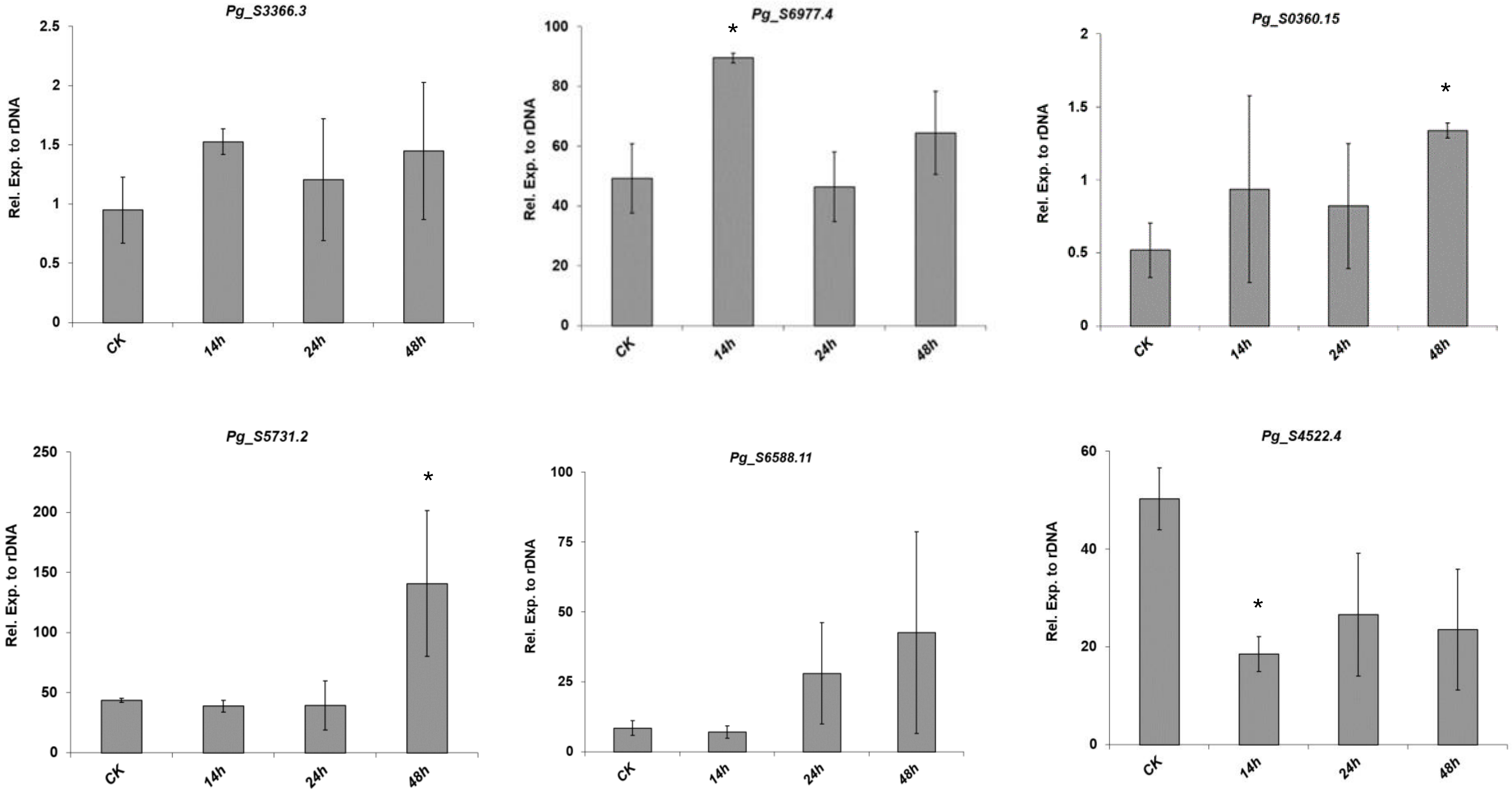
Pg_S3366.3
Pg_S6977.4
Pg_S0360.15
Pg_S5731.2
Pg_S6588.11
Pg_S4522.4
Rel. Exp. to rDNA
CK
14h
24h
48h
*

C

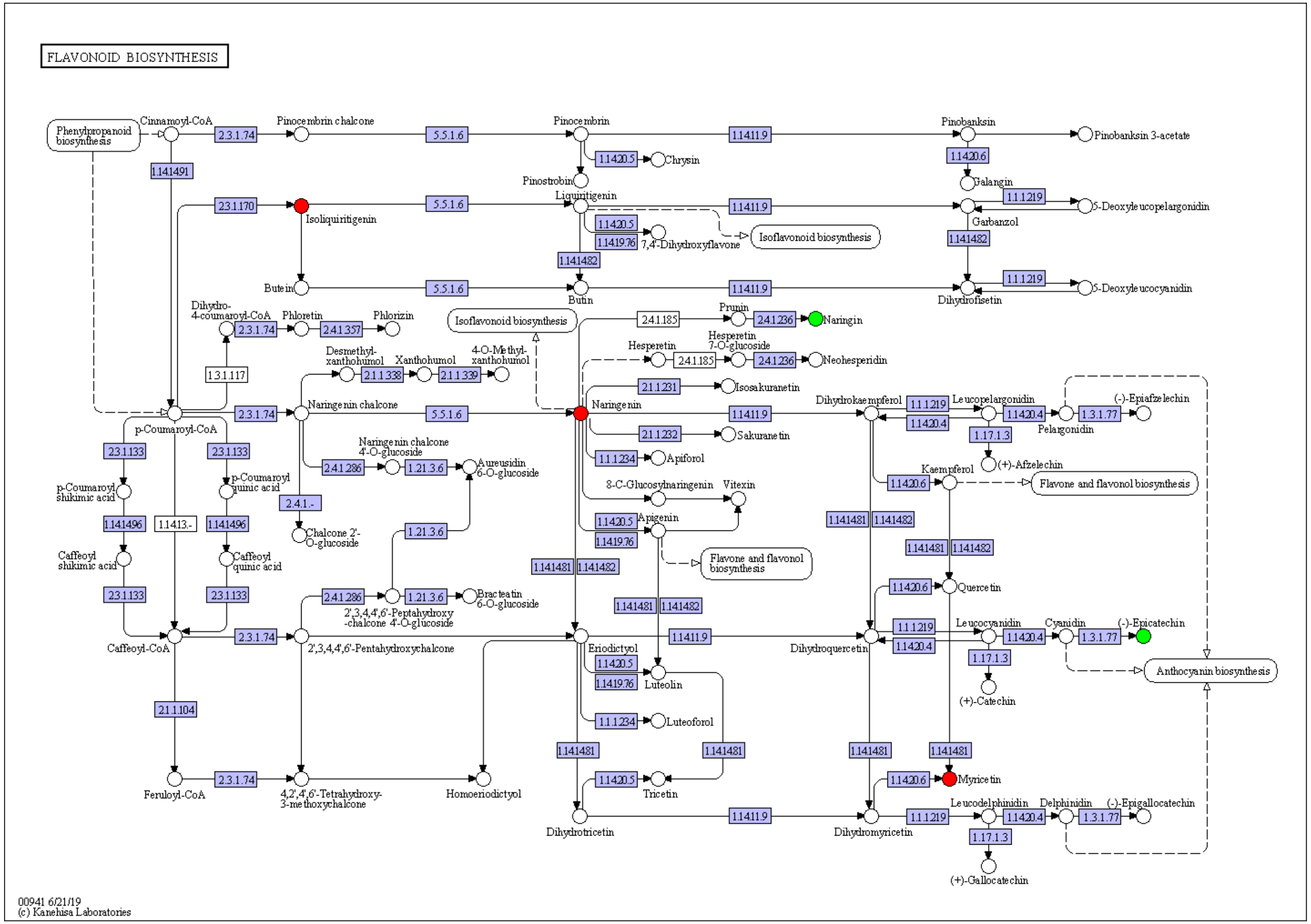

FLAVONOID BIOSYNTHESIS
Phenylpropanoid biosynthesis
Cinnamoyl-CoA
Pinocembrin chalcone
Pinocembrin
Pinobanksin
Pinobanksin 3-acetate
Chrysin
Pinostrobin
Galangin
Isoliquiritigenin
Liquiritigenin
5-Deoxyleucopelargonidin
Garbanzol
7,4'-Dihydroxyflavone
Isoflavonoid biosynthesis
Butein
Butin
Dihydrofisetin
5-Deoxyleucocyanidin
Dihydro-4-coumaroyl-CoA
Phloretin
Phlorizin
Prunin
Naringin
Hesperetin
Hesperetin 7-O-glucoside
Neohesperidin
Desmethyl-xanthohumol
Xanthohumol
4-O-Methyl-xanthohumol
Isosakuranetin
p-Coumaroyl-CoA
Naringenin chalcone
Naringenin
Dihydrokaempferol
Leucopelargonidin
Pelargonidin
(-)-Epiafzelechin
(+)-Afzelechin
Sakuranetin
Apiforol
Naringenin chalcone 4'-O-glucoside
Aureusidin 6-O-glucoside
p-Coumaroyl shikimic acid
p-Coumaroyl quinic acid
Kaempferol
Flavone and flavonol biosynthesis
8-C-Glucosylnaringenin
Vitexin
Apigenin
Chalcone 2'-O-glucoside
Caffeoyl shikimic acid
Caffeoyl quinic acid
Quercetin
Bracteatin 6-O-glucoside
2',3,4,4',6'-Peptahydroxy-chalcone 4'-O-glucoside
Caffeoyl-CoA
2',3,4,4',6'-Pentahydroxychalcone
Eriodictyol
Dihydroquercetin
Leucocyanidin
Cyanidin
(-)-Epicatechin
Anthocyanin biosynthesis
Luteolin
(+)-Catechin
Luteoforol
Feruloyl-CoA
4,2',4',6'-Tetrahydroxy-3-methoxychalcone
Homoeriodictyol
Tricetin
Myricetin
Dihydrotricetin
Dihydromyricetin
Leucodelphinidin
Delphinidin
(-)-Epigallocatechin
(+)-Gallocatechin
2.3.1.74
5.5.1.6
1.14.11.9
1.14.20.5
1.14.20.6
1.14.14.91
2.3.1.170
1.1.1.219
1.14.19.76
1.14.14.82
2.4.1.185
2.4.1.236
2.4.1.357
1.3.1.117
2.1.1.338
2.1.1.339
2.1.1.231
2.1.1.232
2.3.1.133
2.4.1.286
1.21.3.6
1.1.1.234
2.4.1.-
1.14.13.-
1.14.14.96
1.14.14.81
1.14.20.4
1.17.1.3
1.3.1.77
2.1.1.104
00941 6/21/19
(c) Kanehisa Laboratories

Figure S6.

A

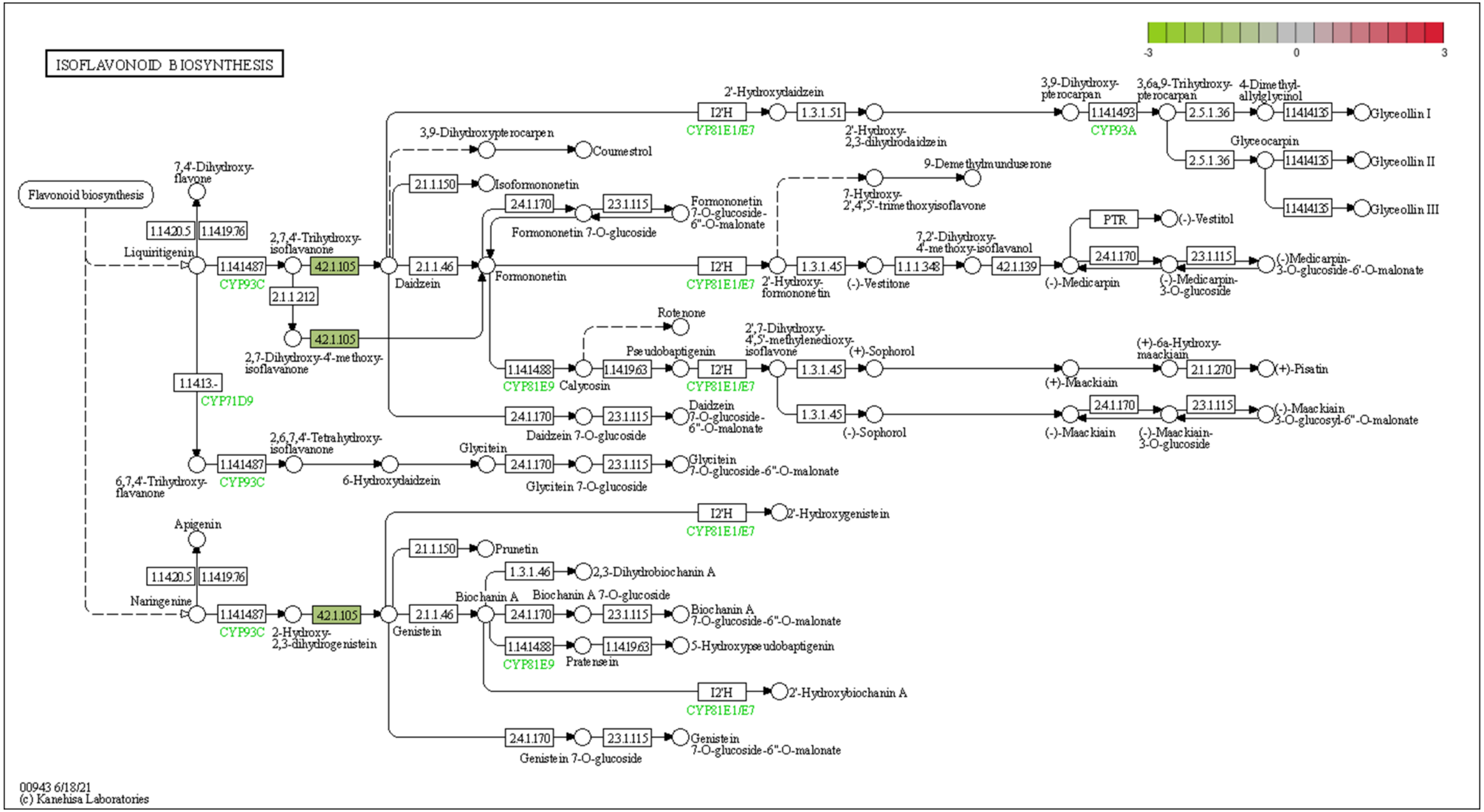
ISOFLAVONOID BIOSYNTHESIS
-3
0
3
Flavonoid biosynthesis
7,4'-Dihydroxy-flavone
Liquiritigenin
2,7,4'-Trihydroxy-isoflavanone
Daidzein
Formononetin
Isoformononetin
3,9-Dihydroxypterocarpen
Coumestrol
Formononetin 7-O-glucoside
Formononetin 7-O-glucoside-6"-O-malonate
2,7-Dihydroxy-4'-methoxy-isoflavanone
2'-Hydroxydaidzein
2'-Hydroxy-2,3-dihydrodaidzein
9-Demethylmunduserone
7-Hydroxy-2',4',5'-trimethoxyisoflavone
2'-Hydroxy-formononetin
(-)-Vestitone
7,2'-Dihydroxy-4'-methoxy-isoflavanol
(-)-Medicarpin
(-)-Vestitol
(-)-Medicarpin-3-O-glucoside
(-)-Medicarpin-3-O-glucoside-6'-O-malonate
3,9-Dihydroxy-pterocarpan
3,6a,9-Trihydroxy-pterocarpan
4-Dimethyl-allylglycinol
Glyceocarpin
Glyceollin I
Glyceollin II
Glyceollin III
Rotenone
Pseudobaptigenin
Calycosin
2',7-Dihydroxy-4',5'-methylenedioxy-isoflavone
(+)-Sophorol
(-)-Sophorol
(+)-Maackiain
(+)-6a-Hydroxy-maackiain
(+)-Pisatin
(-)-Maackiain
(-)-Maackiain-3-O-glucoside
(-)-Maackiain-3-O-glucosyl-6"-O-malonate
Daidzein 7-O-glucoside
Daidzein 7-O-glucoside-6"-O-malonate
6,7,4'-Trihydroxy-flavanone
2,6,7,4'-Tetrahydroxy-isoflavanone
6-Hydroxydaidzein
Glycitein
Glycitein 7-O-glucoside
Glycitein 7-O-glucoside-6"-O-malonate
Apigenin
Naringenine
2-Hydroxy-2,3-dihydrogenistein
Genistein
2'-Hydroxygenistein
Prunetin
Biochanin A
2,3-Dihydrobiochanin A
Biochanin A 7-O-glucoside
Biochanin A 7-O-glucoside-6"-O-malonate
Pratensein
5-Hydroxypseudobaptigenin
2'-Hydroxybiochanin A
Genistein 7-O-glucoside
Genistein 7-O-glucoside-6"-O-malonate
1.14.20.5
1.14.19.76
1.14.14.87
4.2.1.105
2.1.1.212
1.14.13.-
2.1.1.150
2.1.1.46
2.4.1.170
2.3.1.115
I2'H
1.3.1.51
1.14.14.93
2.5.1.36
1.14.14.135
1.3.1.45
1.1.1.348
4.2.1.139
PTR
1.14.14.88
1.14.19.63
2.1.1.270
1.3.1.46
CYP93C
CYP71D9
CYP81E1/E7
CYP81E9
CYP93A
00943 6/18/21
(c) Kanehisa Laboratories

B

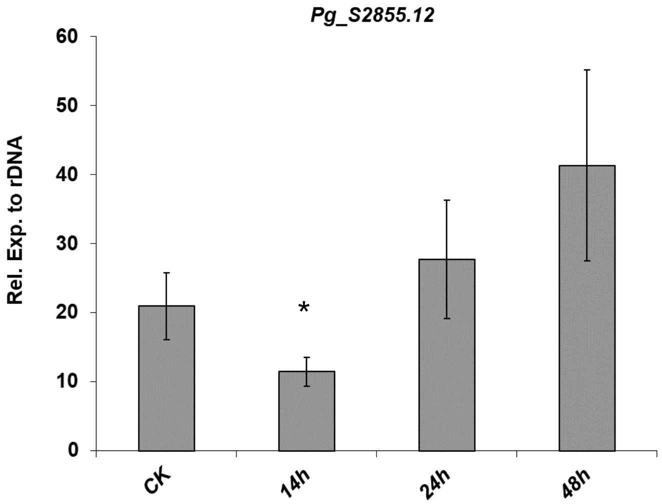

Pg_S2855.12
Rel. Exp. to rDNA
60
50
40
30
20
10
0
*
CK
14h
24h
48h

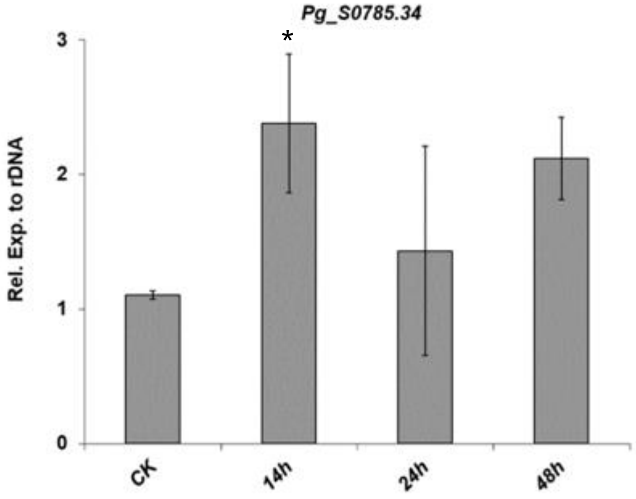

Pg_S0785.34
Rel. Exp. to rDNA
3
2
1
0
*
CK
14h
24h
48h

C

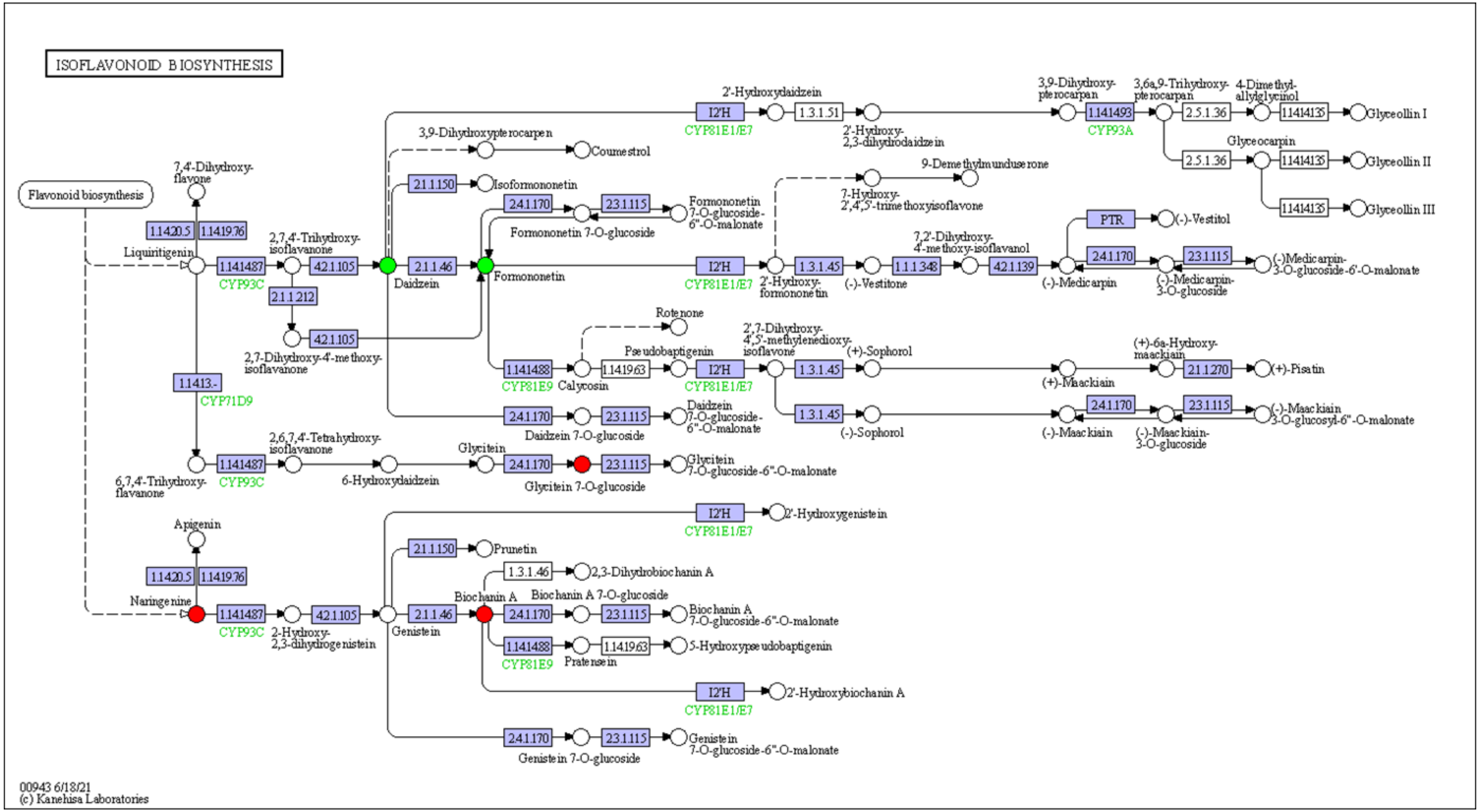

ISOFLAVONOID BIOSYNTHESIS
Flavonoid biosynthesis
7,4'-Dihydroxy-flavone
1.14.20.5
1.14.19.76
Liquiritigenin
1.14.14.87
CYP93C
2,7,4'-Trihydroxy-isoflavanone
4.2.1.105
2.1.1.212
2,7-Dihydroxy-4'-methoxy-isoflavanone
Daidzein
2.1.1.46
Formononetin
2.1.1.150
Isoformononetin
3,9-Dihydroxypterocarpen
Coumestrol
2.4.1.170
2.3.1.115
Formononetin 7-O-glucoside
Formononetin 7-O-glucoside-6"-O-malonate
I2'H
CYP81E1/E7
2'-Hydroxydaidzein
1.3.1.51
2'-Hydroxy-2,3-dihydrodaidzein
3,9-Dihydroxy-pterocarpan
1.14.14.93
CYP93A
3,6a,9-Trihydroxy-pterocarpan
2.5.1.36
4-Dimethyl-allylglycinol
1.14.14.135
Glyceollin I
Glyceocarpin
Glyceollin II
Glyceollin III
9-Demethylmunduserone
7-Hydroxy-2',4',5'-trimethoxyisoflavone
2'-Hydroxy-formononetin
1.3.1.45
(-)-Vestitone
1.1.1.348
7,2'-Dihydroxy-4'-methoxy-isoflavanol
4.2.1.139
(-)-Medicarpin
PTR
(-)-Vestitol
(-)-Medicarpin-3-O-glucoside
(-)-Medicarpin-3-O-glucoside-6'-O-malonate
Rotenone
Pseudobaptigenin
1.14.14.88
CYP81E9
Calycosin
1.14.19.63
2',7-Dihydroxy-4',5'-methylenedioxy-isoflavone
(+)-Sophorol
(+)-Maackiain
(+)-6a-Hydroxy-maackiain
2.1.1.270
(+)-Pisatin
(-)-Sophorol
(-)-Maackiain
(-)-Maackiain-3-O-glucoside
(-)-Maackiain-3-O-glucosyl-6"-O-malonate
1.14.13.-
CYP71D9
Daidzein 7-O-glucoside
Daidzein 7-O-glucoside-6"-O-malonate
6,7,4'-Trihydroxy-flavanone
2,6,7,4'-Tetrahydroxy-isoflavanone
6-Hydroxydaidzein
Glycitein
Glycitein 7-O-glucoside
Glycitein 7-O-glucoside-6"-O-malonate
Apigenin
Naringenine
2-Hydroxy-2,3-dihydrogenistein
Genistein
2'-Hydroxygenistein
Prunetin
1.3.1.46
2,3-Dihydrobiochanin A
Biochanin A
Biochanin A 7-O-glucoside
Biochanin A 7-O-glucoside-6"-O-malonate
Pratensein
5-Hydroxypseudobaptigenin
2'-Hydroxybiochanin A
Genistein 7-O-glucoside
Genistein 7-O-glucoside-6"-O-malonate
00943 6/18/21
(c) Kanehisa Laboratories

Figure S7.

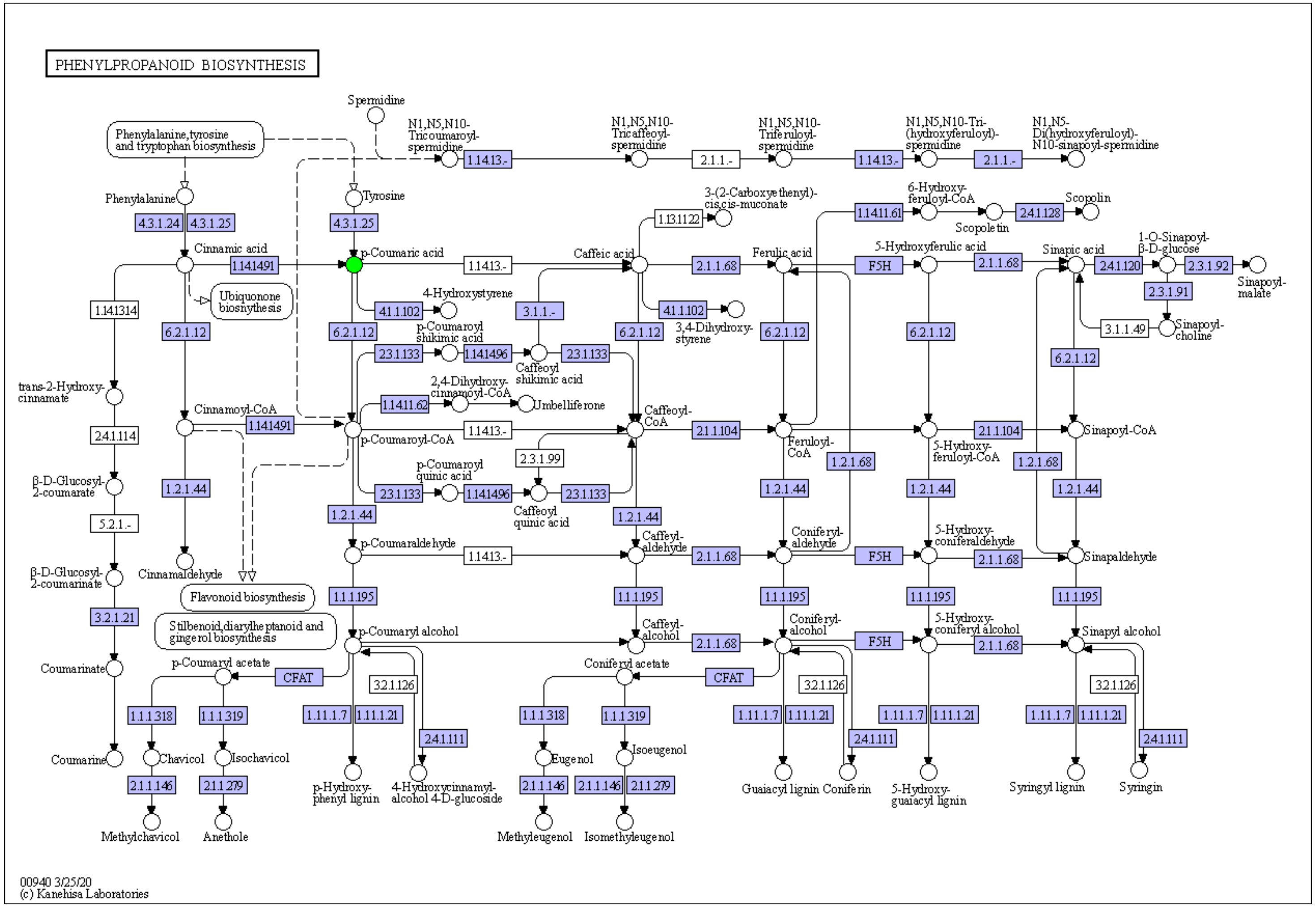
PHENYLPROPANOID BIOSYNTHESIS
Spermidine
Phenylalanine, tyrosine and tryptophan biosynthesis
N1,N5,N10-Tricoumaroyl-spermidine
N1,N5,N10-Tricaffeoyl-spermidine
N1,N5,N10-Triferuloyl-spermidine
N1,N5,N10-Tri-(hydroxyferuloyl)-spermidine
N1,N5-Di(hydroxyferuloyl)-N10-sinapoyl-spermidine
1.14.13.-
2.1.1.-
Phenylalanine
Tyrosine
3-(2-Carboxyethenyl)-cis,cis-muconate
6-Hydroxy-feruloyl-CoA
Scopoletin
Scopolin
4.3.1.24
4.3.1.25
1.13.11.22
1.14.11.61
2.4.1.128
Cinnamic acid
p-Coumaric acid
Caffeic acid
Ferulic acid
5-Hydroxyferulic acid
Sinapic acid
1-O-Sinapoyl-β-D-glucose
Sinapoyl-malate
1.14.14.91
1.14.13.-
2.1.1.68
F5H
2.4.1.120
2.3.1.92
2.3.1.91
Ubiquinone biosynthesis
4-Hydroxystyrene
3,4-Dihydroxy-styrene
4.1.1.102
3.1.1.-
1.14.13.14
6.2.1.12
p-Coumaroyl shikimic acid
Caffeoyl shikimic acid
2.3.1.133
1.14.14.96
3.1.1.49
Sinapoyl-choline
trans-2-Hydroxy-cinnamate
2,4-Dihydroxy-cinnamoyl-CoA
Umbelliferone
1.14.11.62
Cinnamoyl-CoA
p-Coumaroyl-CoA
Caffeoyl-CoA
Feruloyl-CoA
5-Hydroxy-feruloyl-CoA
Sinapoyl-CoA
2.4.1.114
2.1.1.104
2.3.1.99
1.2.1.68
β-D-Glucosyl-2-coumarate
p-Coumaroyl quinic acid
Caffeoyl quinic acid
1.2.1.44
5.2.1.-
Cinnamaldehyde
p-Coumaraldehyde
Caffeyl-aldehyde
Coniferyl-aldehyde
5-Hydroxy-coniferaldehyde
Sinapaldehyde
β-D-Glucosyl-2-coumarinate
Flavonoid biosynthesis
1.1.1.195
3.2.1.21
Stilbenoid, diarylheptanoid and gingerol biosynthesis
p-Coumaryl alcohol
Caffeyl-alcohol
Coniferyl-alcohol
5-Hydroxy-coniferyl alcohol
Sinapyl alcohol
Coumarinate
p-Coumaryl acetate
Coniferyl acetate
CFAT
3.2.1.126
1.1.1.318
1.1.1.319
1.11.1.7
1.11.1.21
2.4.1.111
Coumarine
Chavicol
Isochavicol
Eugenol
Isoeugenol
2.1.1.146
2.1.1.279
p-Hydroxy-phenyl lignin
4-Hydroxycinnamyl-alcohol 4-D-glucoside
Guaiacyl lignin
Coniferin
5-Hydroxy-guaiacyl lignin
Syringyl lignin
Syringin
Methylchavicol
Anethole
Methyleugenol
Isomethyleugenol
00940 3/25/20
(c) Kanehisa Laboratories

Figure S8.

A

48h 72h 96h

CK treatment CK treatment CK treatment

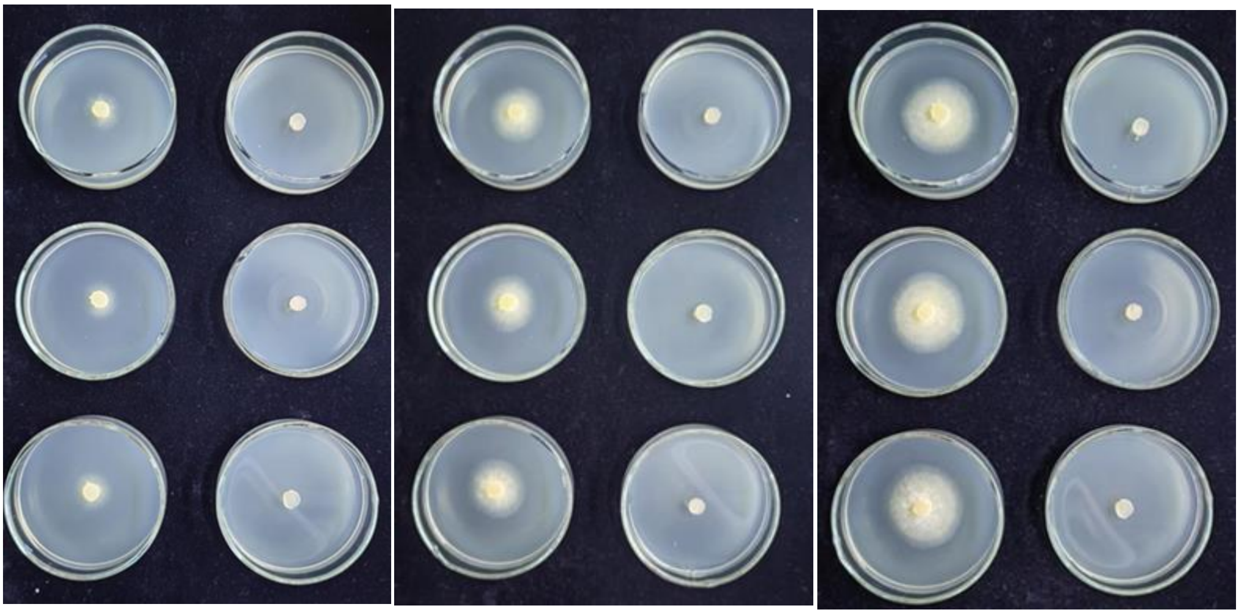

p-Coumaric acid (10.0 µmol/mL)

B

48h 72h 96h

CK treatment CK treatment CK treatment

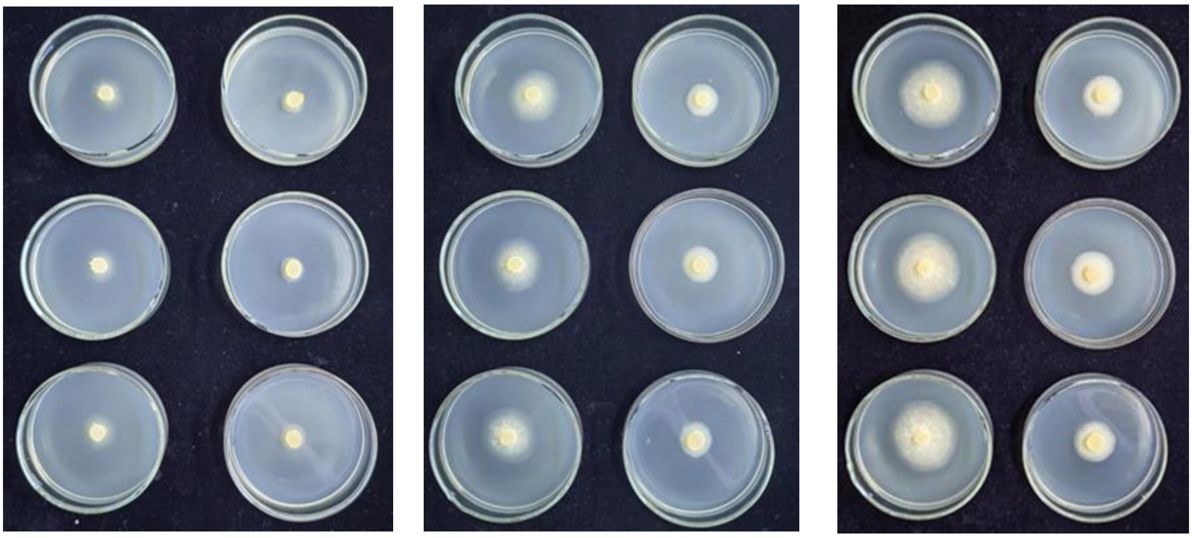

Puerarin (10.0 µmol/mL)

C

48h | 72h | 96h

CK treatment | CK treatment | CK treatment

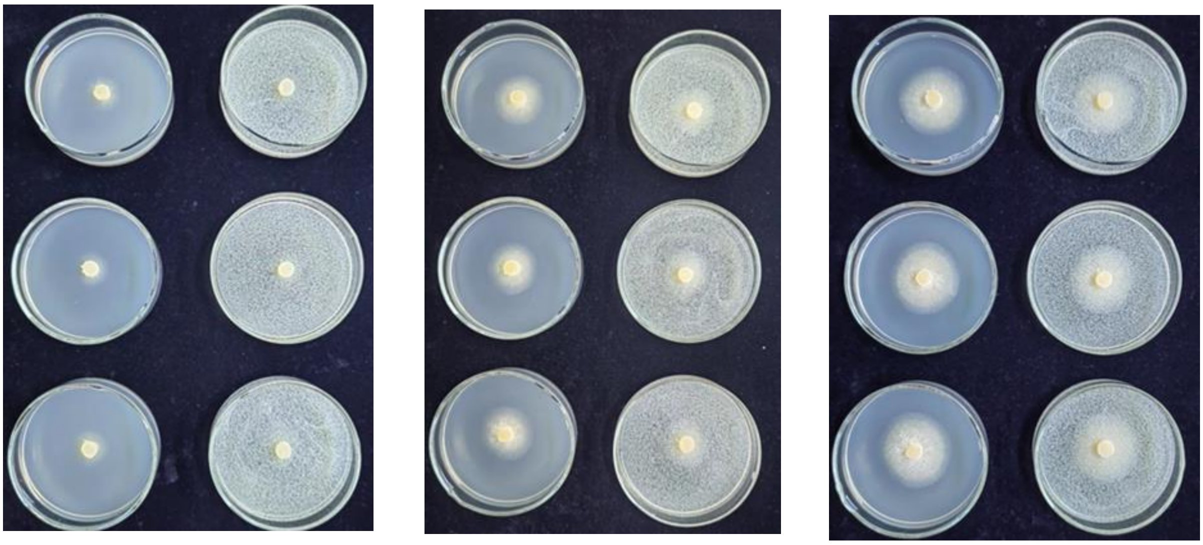

Naringin (10.0 μmol/mL)

D

48h | 72h | 96h

CK treatment | CK treatment | CK treatment

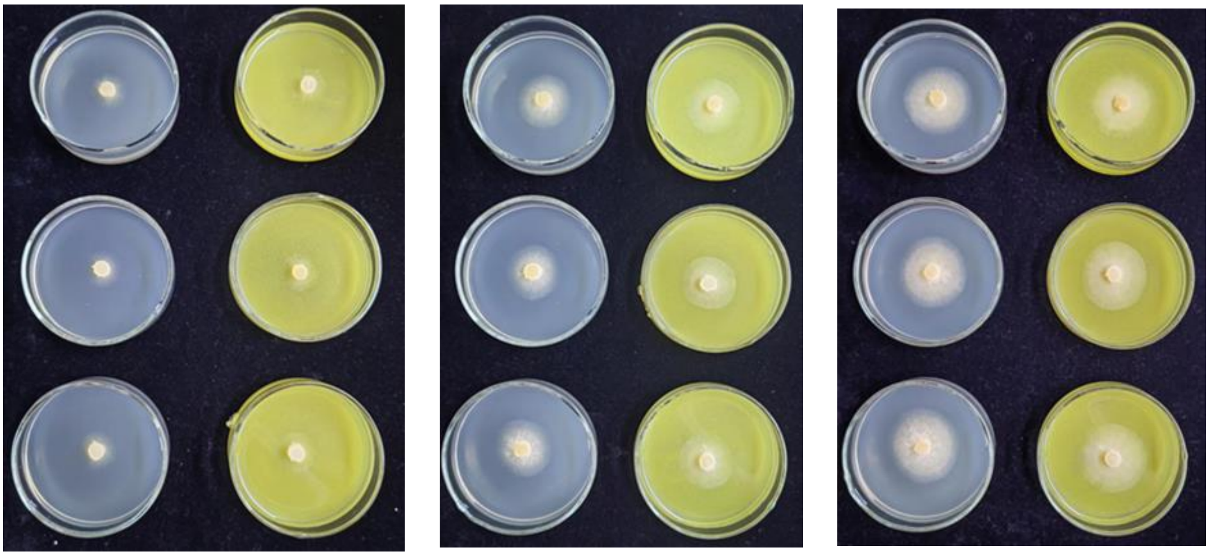

Myricetin (10.0 μmol/mL)

E

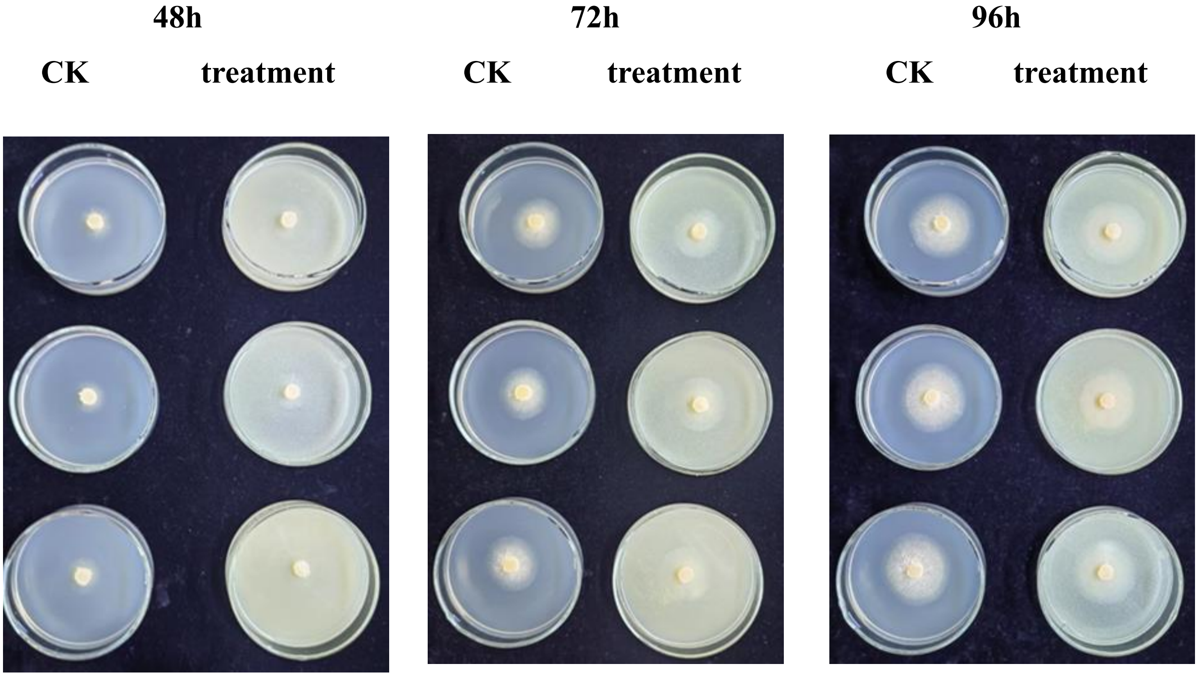

Chrysin (10.0 µmol/mL)

F

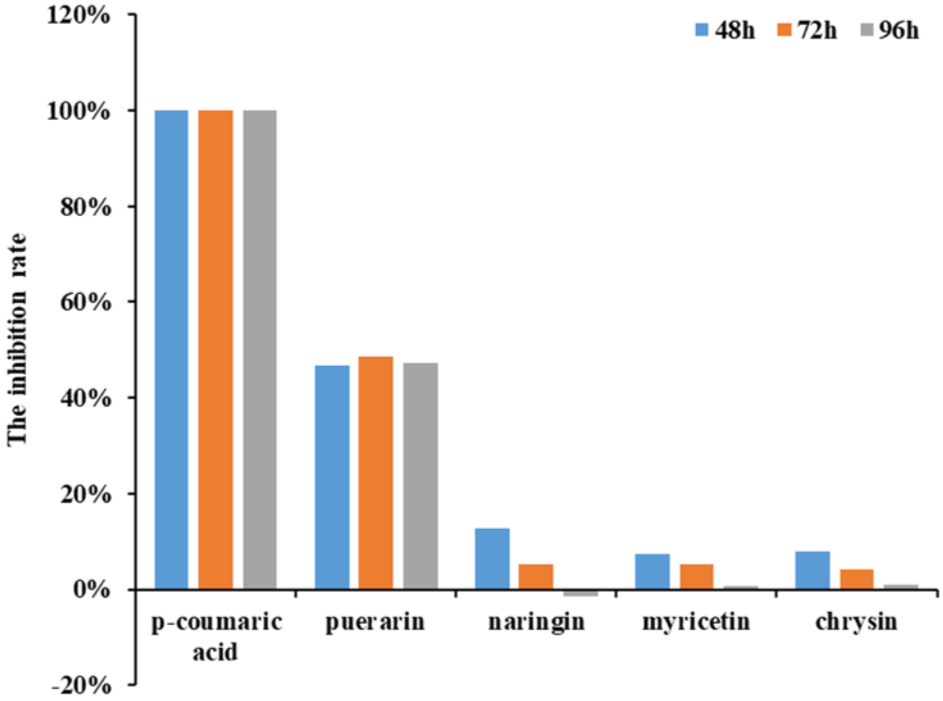

Figure S9.

Supplementary Table 1. Linear and Mass information of standard components used in this study.

NO.	Component Name	Mass Info	Retention Time	Linear	R	Linear range(ng/mL)
1	Rutin	611.0 / 303.0	5.019887044	y = 2.49832e-4 x + -3.71659e-4	0.99973	0.1-500
2	Daidzin	417.0 / 255.0	4.344905397	y = 4.72168e-4 x + -0.00266	0.99848	0.1-500
3	Genistin	433.0 / 271.0	5.717388985	y = 6.90946e-4 x + 2.87693e-4	0.9993	0.1-500
4	p-Coumaric acid	165.0 / 147.0	4.617662294	y = 6.30695e-4 x + -0.00154	0.99999	0.1-500
5	Luteolin	287.0 / 153.0	7.964607341	y = 1.61206e-4 x + -5.21800e-4	0.9996	0.1-500
6	kaempferol	287.0 / 153.0	9.002093023	y = 2.61461e-5 x + -3.52723e-4	0.99955	0.1-500
7	Taxifolin	305.0 / 153.0	5.453711654	y = 4.80804e-5 x + 2.73326e-4	0.99985	0.1-500
8	Myricetin	319.0 / 153.0	6.74205291	y = 3.76127e-5 x + -3.48528e-4	0.99949	0.1-500
9	Dihydrokaempferol	289.0 / 153.0	6.718815553	y = 392.86803 x + -247.04988	0.99947	0.1-500
10	Glycitin	447.0 / 285.0	4.525677007	y = 2510.10833 x + -12892.48631	0.99933	0.1-500
11	Quercetin 3-O-glucoside	465.0 / 303.0	5.388195042	y = 3.43793e-4 x + -0.00183	0.99873	0.1-500
12	Daidzein	255.0 / 199.0	7.564633039	y = 1.19028e-4 x + 2.58607e-4	0.99941	0.1-500
13	Genistein	271.0 / 153.0	8.841495741	y = 2111.52969 x + -11137.74669	0.99846	0.1-500
14	Eriodictyol	289.0 / 153.0	7.734616386	y = 1613.94702 x + -9910.24347	0.9986	0.1-500
15	Sakuranetin	287.0 / 167.0	10.53306667	y = 0.00142 x + -0.01002	0.99826	0.1-500
16	Quercetin	303.0 / 153.0	7.972306465	y = 9.56121e-5 x + -0.00146	0.99908	0.1-500
17	Isorhamnetin	317.0 / 302.0	9.23007645	y = 3.49695e-4 x + -0.00268	0.99965	0.1-500
18	Luteolin-7-O-glucoside	449.0 / 287.0	5.51286316	y = 1373.15001 x + 1806.34976	0.99996	0.1-500
19	Puerarin	285.0 / 242.0	10.65658569	y = 4.08111e-4 x + 0.00164	0.99951	0.1-500
20	phenylalanine	166.1 / 103.1	1.886351774	y = 3.98615e-4 x + 0.00351	0.99974	0.1-500
21	Naringin	579.0 / 271.0	6.312893186	y = 1613.25261 x + -7884.98839	0.99966	0.1-500

22	Glycetein	283.0 / 268.0	7.872124109	y = 0.00290 x + -0.01764	0.99846	0.1-500
23	Formononetin	267.0 / 223.0	9.907369219	y = 0.00336 x + -0.00684	0.9999	0.1-500
24	Isoliquiritigenin	255.0 / 119.0	9.653416944	y = 15436.31503 x + -9.81769e4	0.99925	0.1-500
25	Biochanin A	283.0 / 268.0	10.66245	y = 27444.78212 x + -1.33827e5	0.99927	0.1-500
26	Naringenin	271.0 / 151.0	8.745231449	y = 0.00321 x + -0.00687	0.99987	0.1-500
27	catechin	289.0 / 245.0	3.299372551	y = 1235.02478 x + -9821.66631	0.99876	0.1-500
28	Epicatechin	289.0 / 203.0	3.865833333	y = 809.93887 x + -4027.73952	0.99954	0.1-500
29	(-)-Epigallocatechin	305.0 / 125.0	2.993866667	y = 4.34809e-4 x + -0.00133	0.99995	0.1-500
30	Butin	271.0 / 135.0	6.634269712	y = 0.00440 x + -0.00268	0.99999	0.1-500
31	Quercitrin	447.0 / 301.0	6.329835398	y = 4241.91353 x + -4.03938e4	0.9985	0.1-500
32	Liquiritigenin	255.0 / 135.0	5.069661853	y = 0.00130 x + -0.01796	0.99893	0.1-500
33	Apigenin	269.0 / 117.0	7.681022435	y = 0.00283 x + -0.01299	0.99958	0.1-500
34	Chrysin	253.0 / 143.0	10.52222245	y = 10165.22727 x + -7.79340e4	0.9993	0.1-500
35	(+)-Gallocatechin	305.0 / 125.0	2.211565212	y = 7.08366e-4 x + -0.00138	0.99982	0.1-500
36	Ferulic acid	193.0 / 134.0	5.220137128	y = 3.79760e-4 x + -0.00551	0.99849	0.1-500
37	Vitexin/Isovitexin	431.0 / 311.0	5.065162898	y = 2486.60051 x + -12507.99751	0.99948	0.1-500
38	Kaempferide	299.0 / 284.0	10.63769142	y = 20123.00419 x + -2.91382e5	0.99835	0.1-500

Supplementary Table 2. Quantitative analysis of selected ginseng compounds (ng/g) in untreated control (CK) or *C. panacicola* FSL treated ginseng leaves (CpFSL).

Metabolite Name	Transitions	Retention Time(min)	QC RSD	Fold change	p-value	CK-1	CK-2	CK-3	CK-4	CpFSL-1	CpFSL-2	CpFSL-3	CpFSL-4
Chrysin	253.0 / 143.0	10.52	0.12		0.00	0.36	0.19	0.17	0.16	0.00	0.00	0.00	0.00
Vitexin/Isovitexin	431.0 / 311.0	5.07	0.08	2.67	0.00	4.35	2.83	4.06	3.30	10.31	7.99	11.35	9.25
Glycitin	447.0 / 285.0	4.53	0.12	1.24	0.00	689.01	562.33	654.87	701.79	776.93	810.50	846.90	807.00
Epicatechin	289.0 / 203.0	3.87	0.06	0.42	0.01	5.54	4.03	4.35	3.28	1.46	1.08	2.66	2.04
Luteolin-7-O-glucoside	449.0 / 287.0	5.51	0.09	1.19	0.01	221.34	187.69	210.41	192.03	235.60	238.07	255.93	236.29
Naringin	579.0 / 271.0	6.31	0.06	0.57	0.01	0.74	0.62	0.96	0.83	0.43	0.44	0.31	0.60
catechin	289.0 / 245.0	3.30	0.14	0.94	0.52	11.26	11.57	11.59	8.24	9.20	10.12	10.08	10.88
Naringenin	271.0 / 151.0	8.75	0.04	1.14	0.58	1.03	0.51	0.50	0.51	0.77	0.57	0.66	0.90
Daidzein	255.0 / 199.0	7.56	0.13	0.94	0.58	38.74	48.60	34.15	38.29	41.94	41.75	32.40	34.58
p-Coumaric acid	165.0 / 147.0	4.62	0.05	0.94	0.65	73.16	107.91	89.06	104.59	72.88	85.92	87.01	108.18
Biochanin A	283.0 / 268.0	10.66	0.13	1.01	0.96	1.01	0.90	0.32	0.96	0.76	0.79	0.88	0.78
Myricetin	319.0 / 153.0	6.74	0.12	1.00	0.98	25.90	33.41	38.83	29.90	31.92	34.93	33.34	28.16
phenylalanine	166.1 / 103.1	1.89	0.01	1.03	0.33	15749.31	16530.26	16888.34	16068.71	16748.66	15038.66	15246.24	16198.63
Kaempferide	299.0 / 284.0	10.64	0.18	0.87	0.26	0.41	0.31	0.27	0.38	0.27	0.30	0.33	0.29
Liquiritigenin	255.0 / 135.0	5.07	0.19	2.60	0.00	6.20	3.55	4.21	3.24	11.02	10.69	11.24	11.80
Puerarin	285.0 / 242.0	10.66	0.20	0.44	0.00	2.22	1.69		1.60	0.80	0.81	0.69	0.90
Formononetin	267.0 / 223.0	9.91	0.21	0.16	0.00	0.41	0.42	0.29	0.46	0.05	0.06	0.00	0.07

Supplementary Table 3. Primers used in this study.

Gene	Forward primer (5' – 3')	Reverse primer (5' – 3')
Pg_S4522.4	GTTTGGCAGTACGGGTATCG	AGAACCAGCACCTCAACACTG
Pg_S3366.3	TTATGGGCTGCTCTGGAGG	TGCTTCCTGGATTGTTCTCAG
Pg_S6977.4	TGTATTTCATCGAGCCGTTGT	TCAGTGAGTTTCTTGGGAGCA
Pg_S0360.15	CAACTTGGCACACTCGTCC	TCCCACTTCTCAATCAACTGG
Pg_S5731.2	TGATTTCGTATTTGTGGACGC	TTCCTCATCGGAGCATCG
Pg_S6588.11	TTCTCACGAGCCCGATGAC	CCATTGAACAGGTTTGCTTCC
Pg_S2855.12	TCCGTAGATTACAGGGCGAG	TTGTCAAGGGTGTGTGATGC
Pg_S0785.34	TTGCTTGTTTGTGTTGCGTC	CATCCACTTTCTTTCACCGC
rDNA	TATTCTGGTGTCCTAGGCGT	ATCCTGGCGTCGAGCTATTT
