# Supplementary material for: Transcriptome and metabolite analyses indicated the underlying molecular responses of Asian ginseng (Panax ginseng) toward Colletotrichum panacicola infection
Source: Front Plant Sci. 2023 Jul 10;14:1182685. doi: 10.3389/fpls.2023.1182685 (PMC10365858; doi:10.3389/fpls.2023.1182685)
Supplement: Supplementary file 3 [file DataSheet_3.pdf]

XIC from CK-1.wiff (sample 1) - CK-1, Experiment 2, -MRM (38 transitions): NEG_MSTD0411_q (579.0 / 271.0)

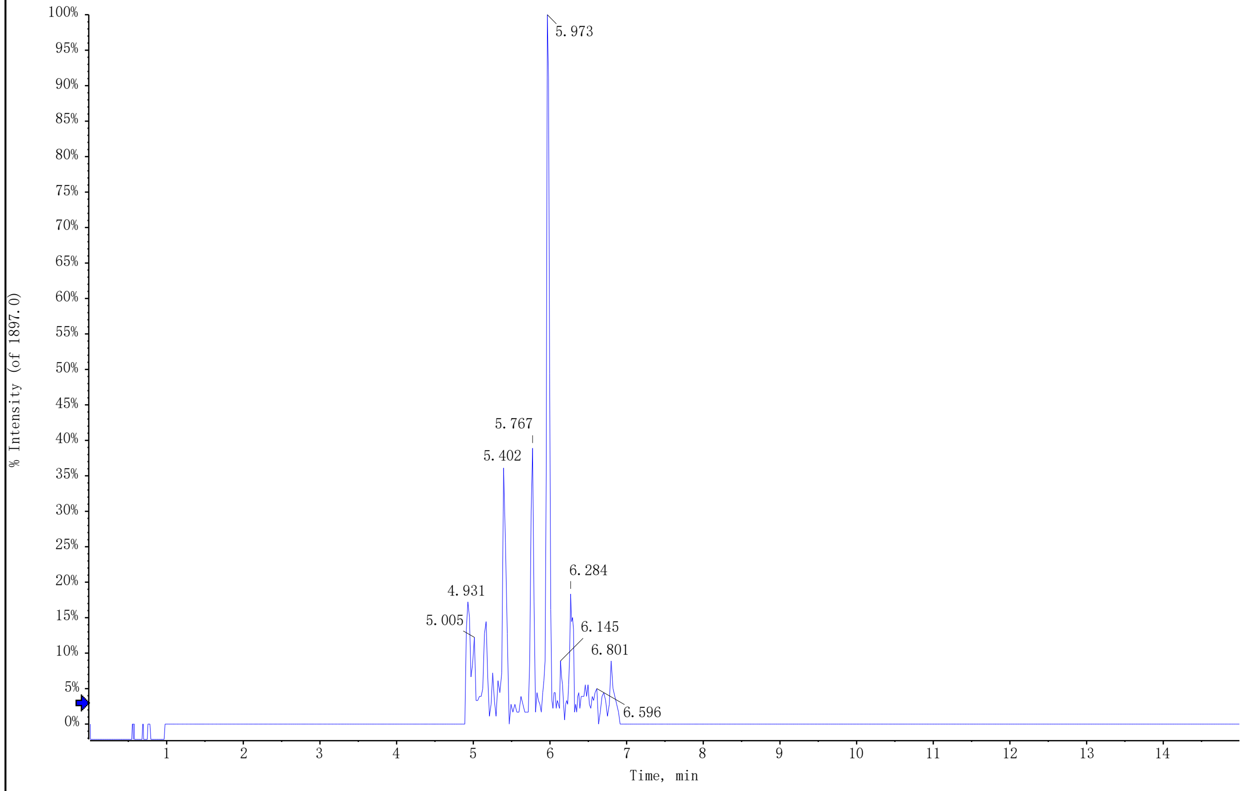

2023/5/9 22:36:21

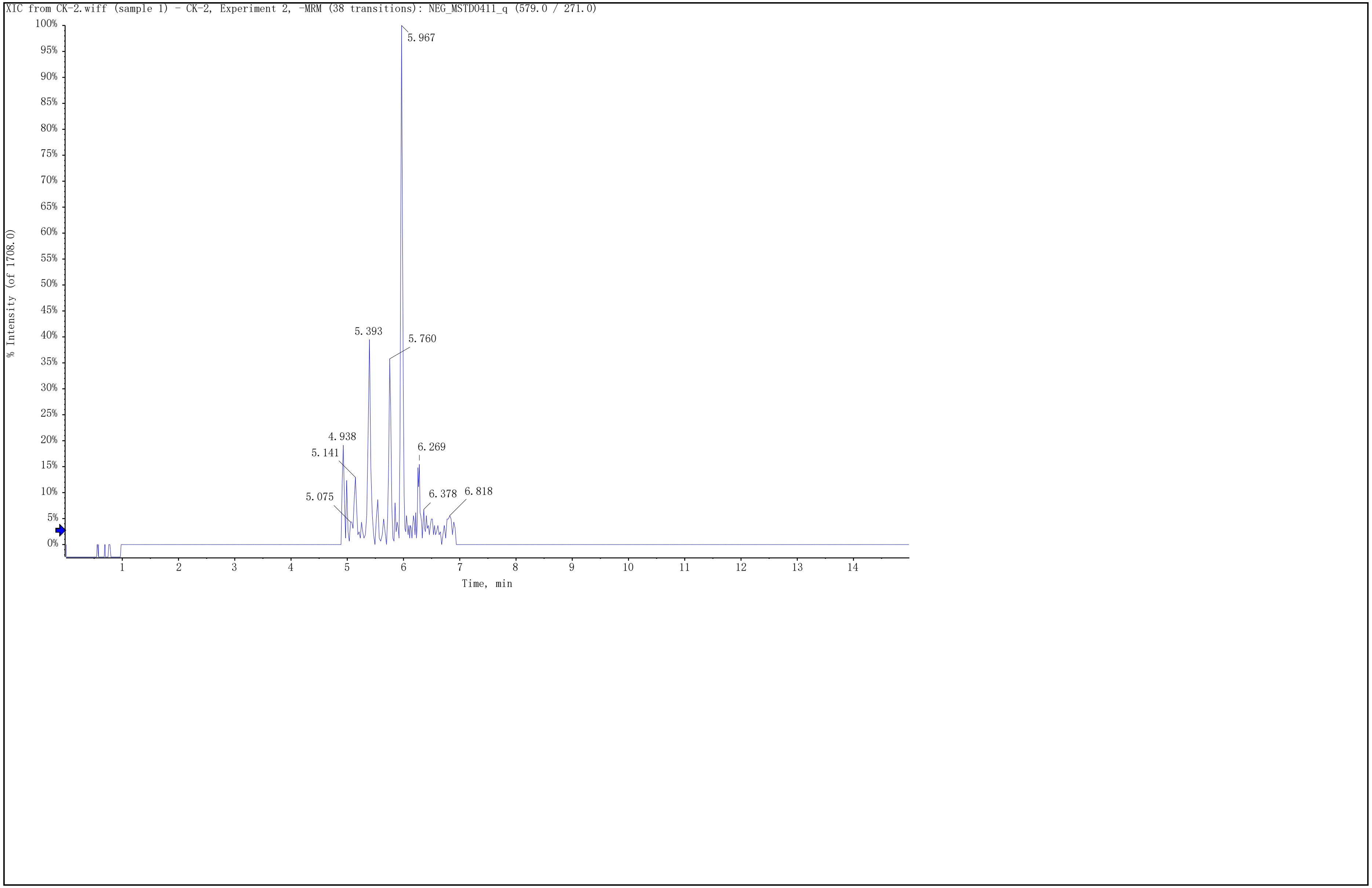

2023/5/10 15:06:18

XIC from CK-3.wiff (sample 1) - CK-3, Experiment 2, -MRM (38 transitions): NEG_MSTD0411_q (579.0 / 271.0)

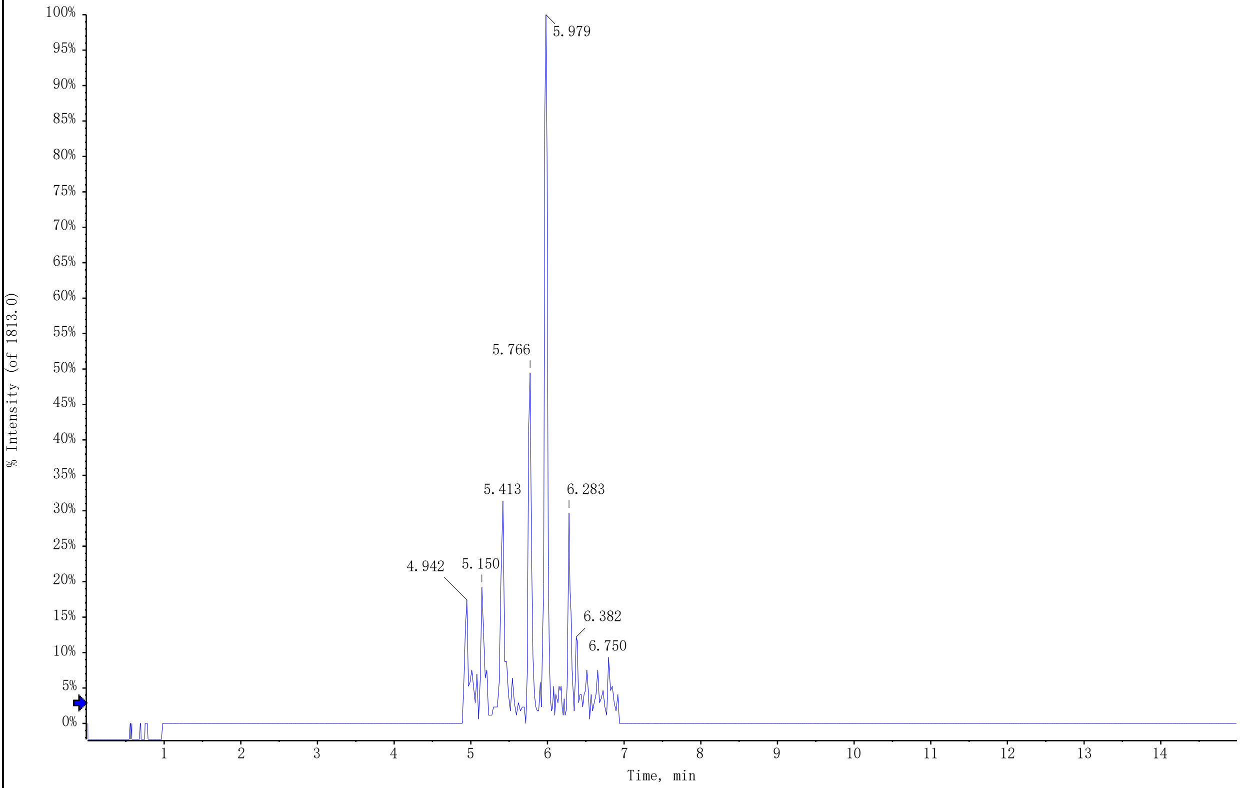

2023/5/10 15:28:04

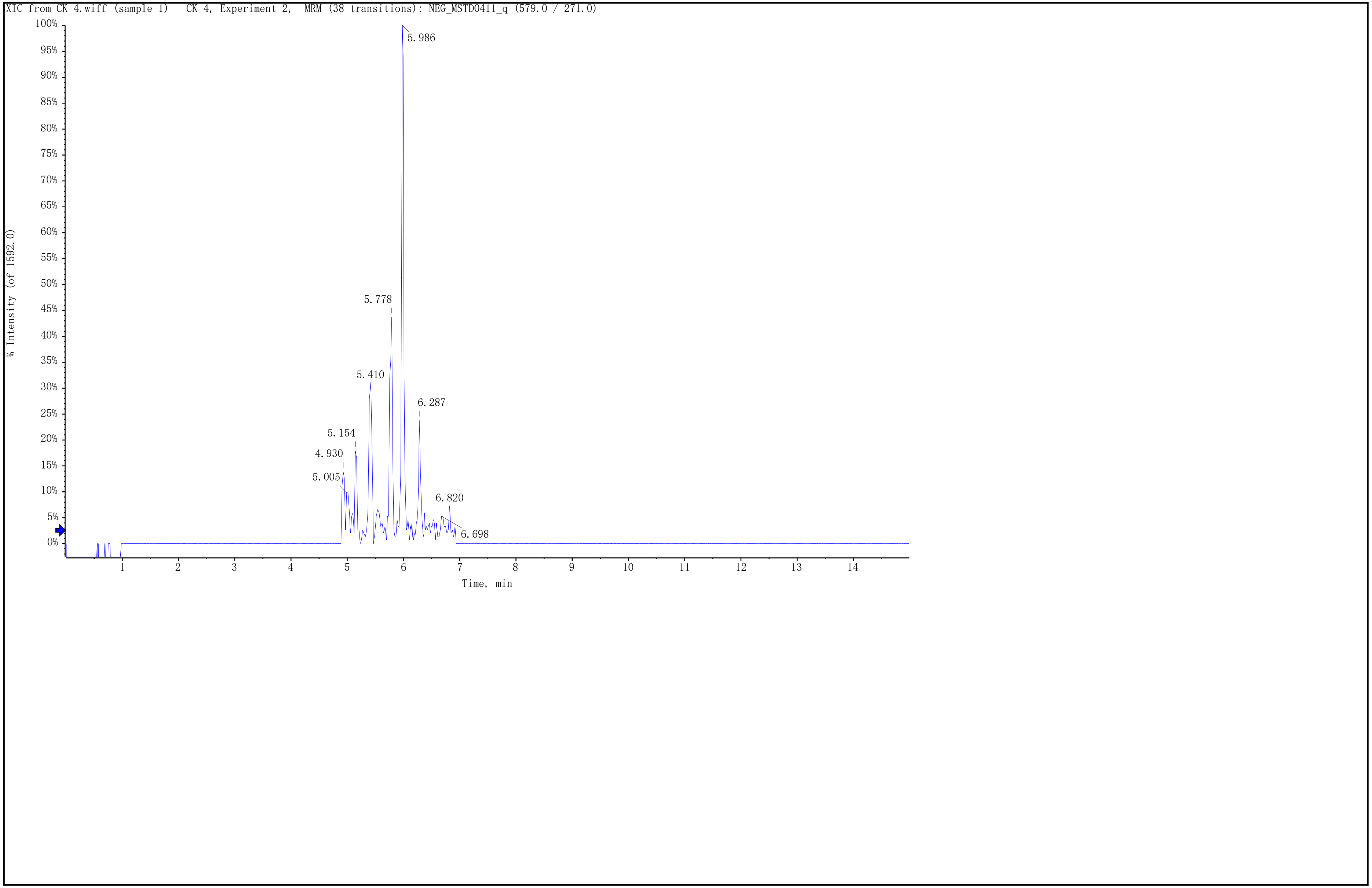

2023/5/10 15:50:12

XIC from CpFSL-1.wiff (sample 1) - CpFSL-1, Experiment 2, -MRM (38 transitions): NEG_MSTD0411_q (579.0 / 271.0)

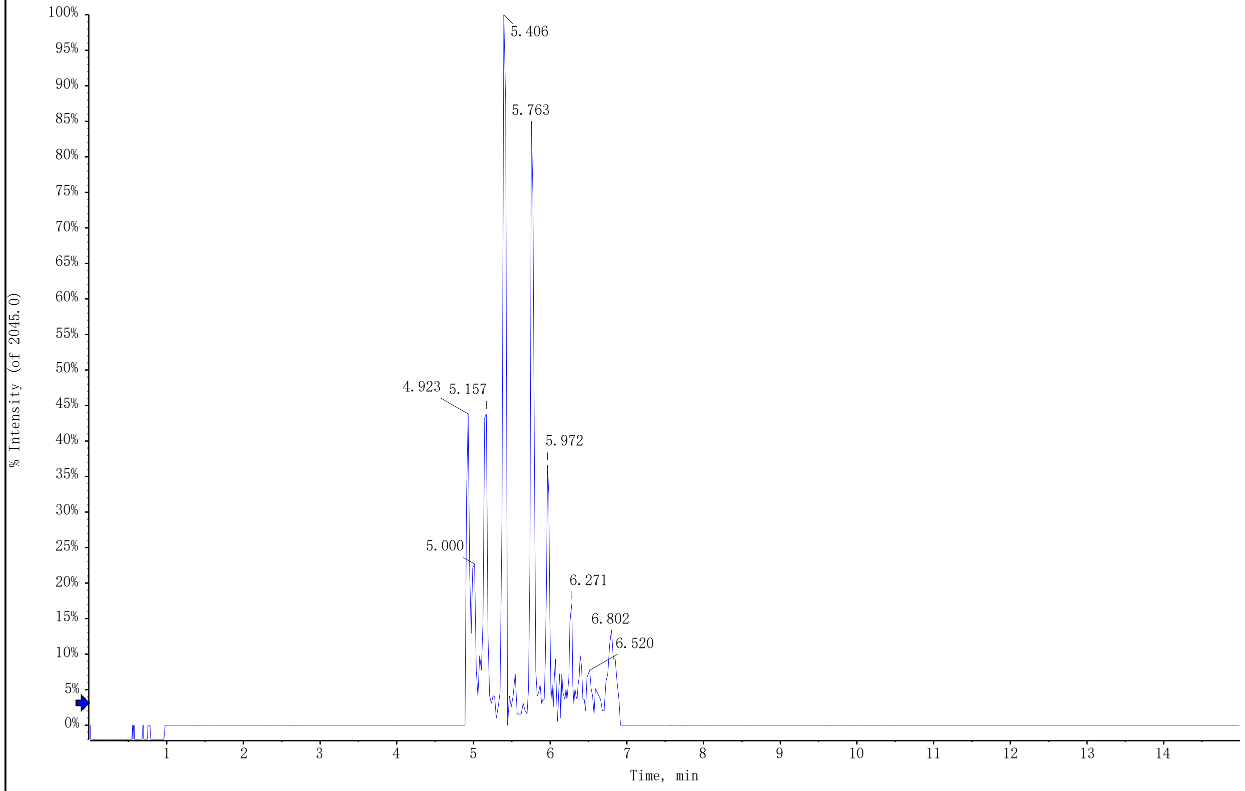

2023/5/10 16:14:07

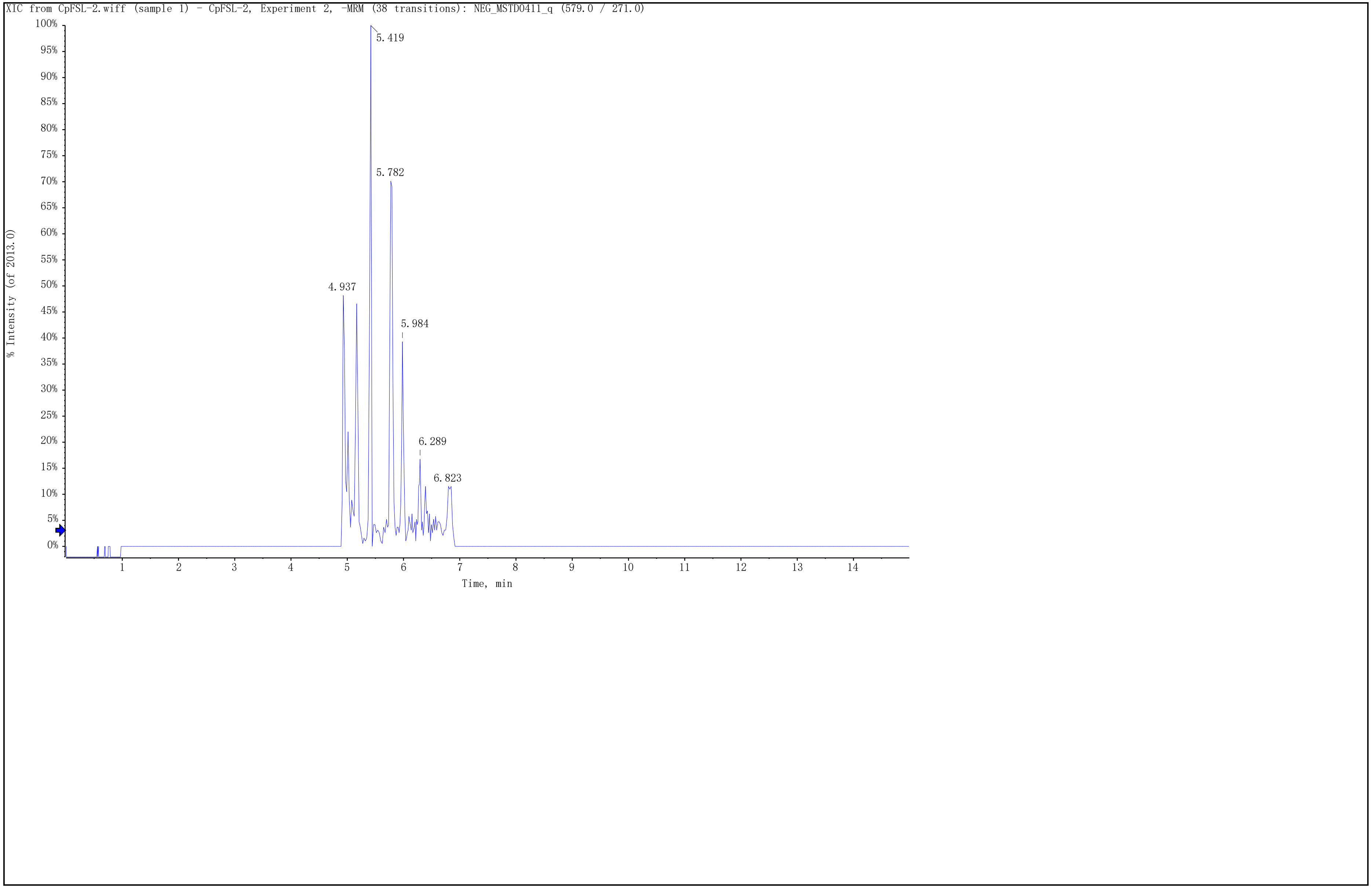

2023/5/10 16:41:24

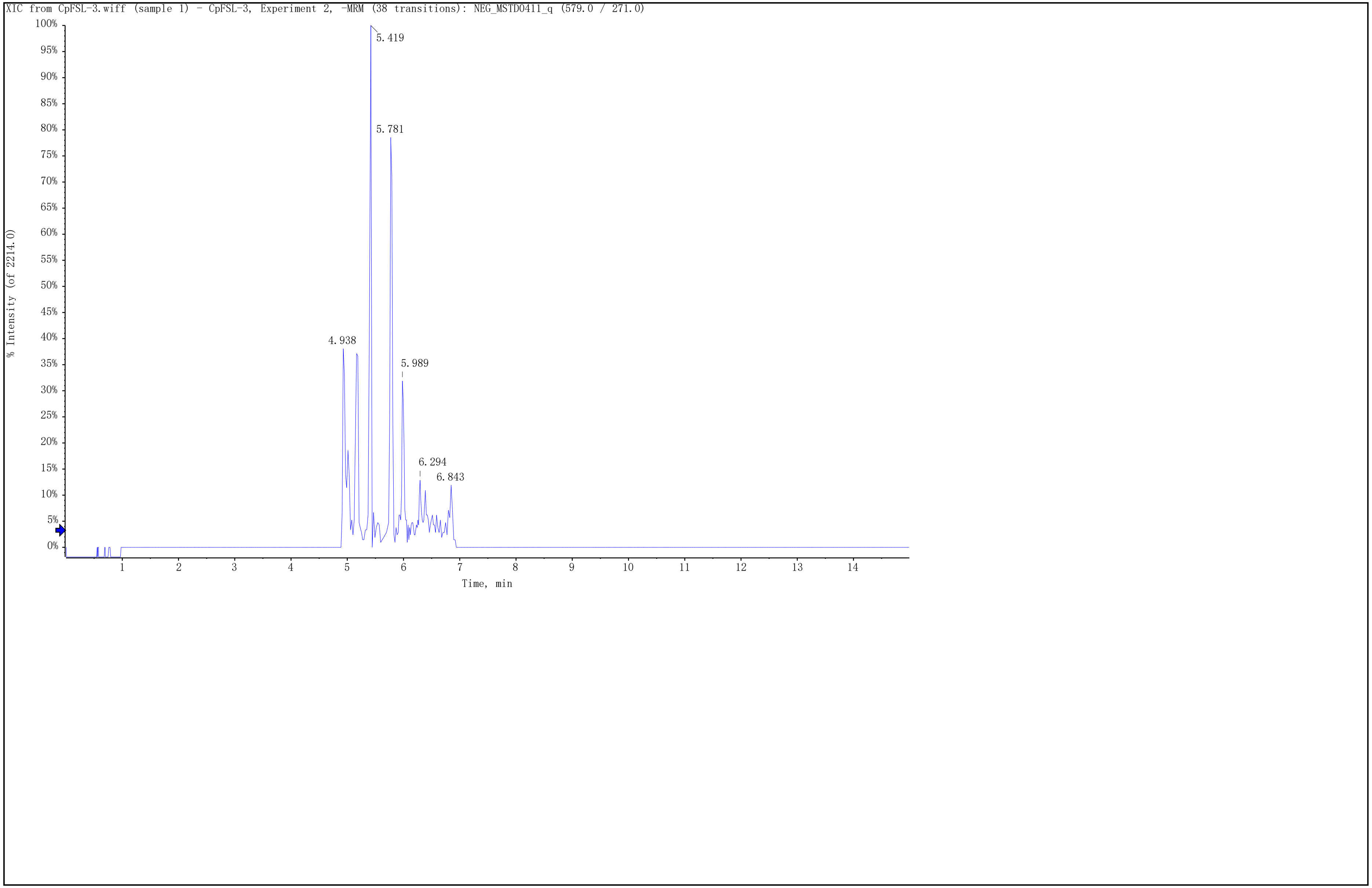

2023/5/10 22:34:26

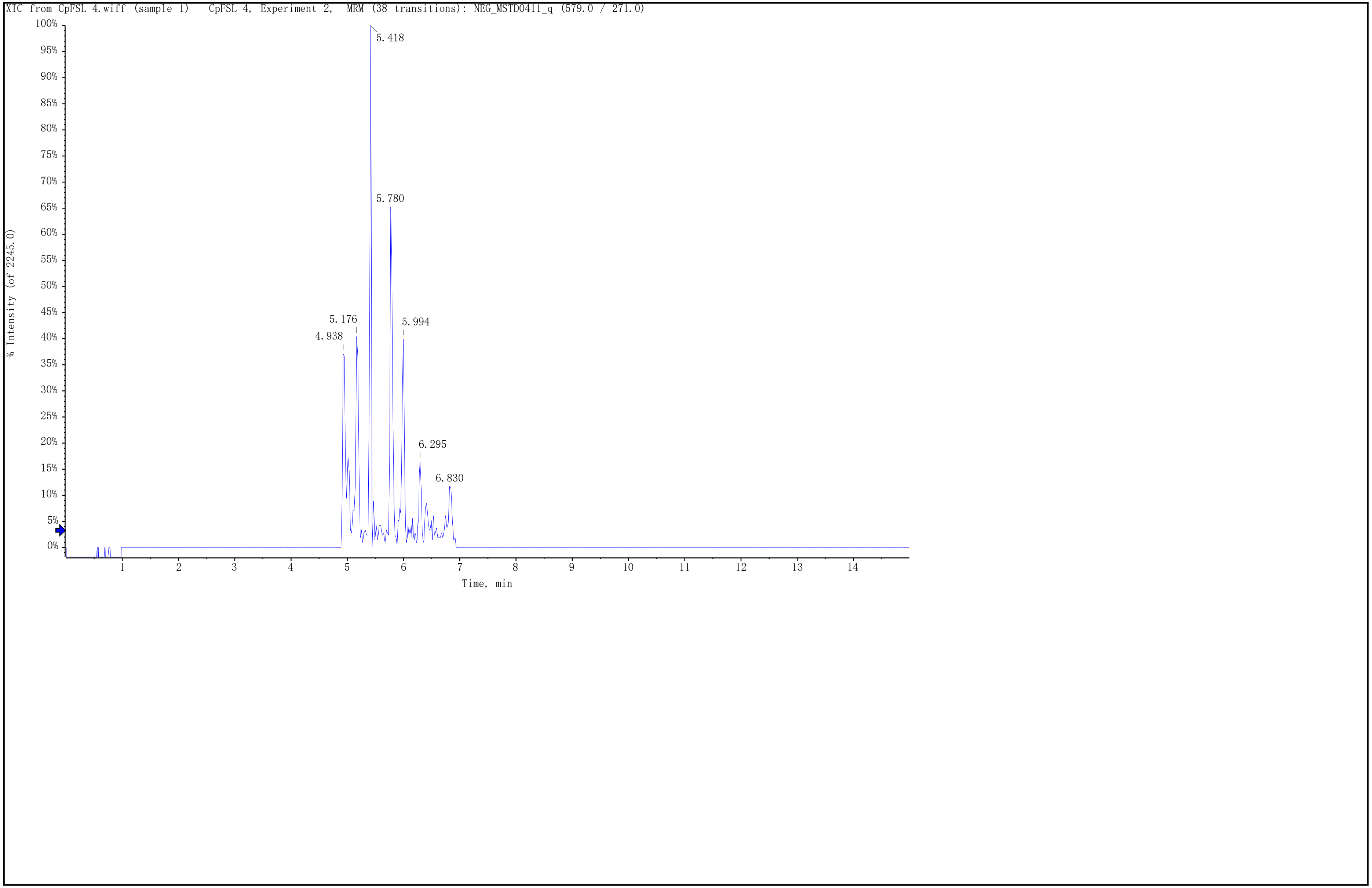

2023/5/11 16:21:17

XIC from CK-1.wiff (sample 1) - CK-1, Experiment 1, +MRM (41 transitions): POS_MSTD0447_q (449.0 / 287.0)

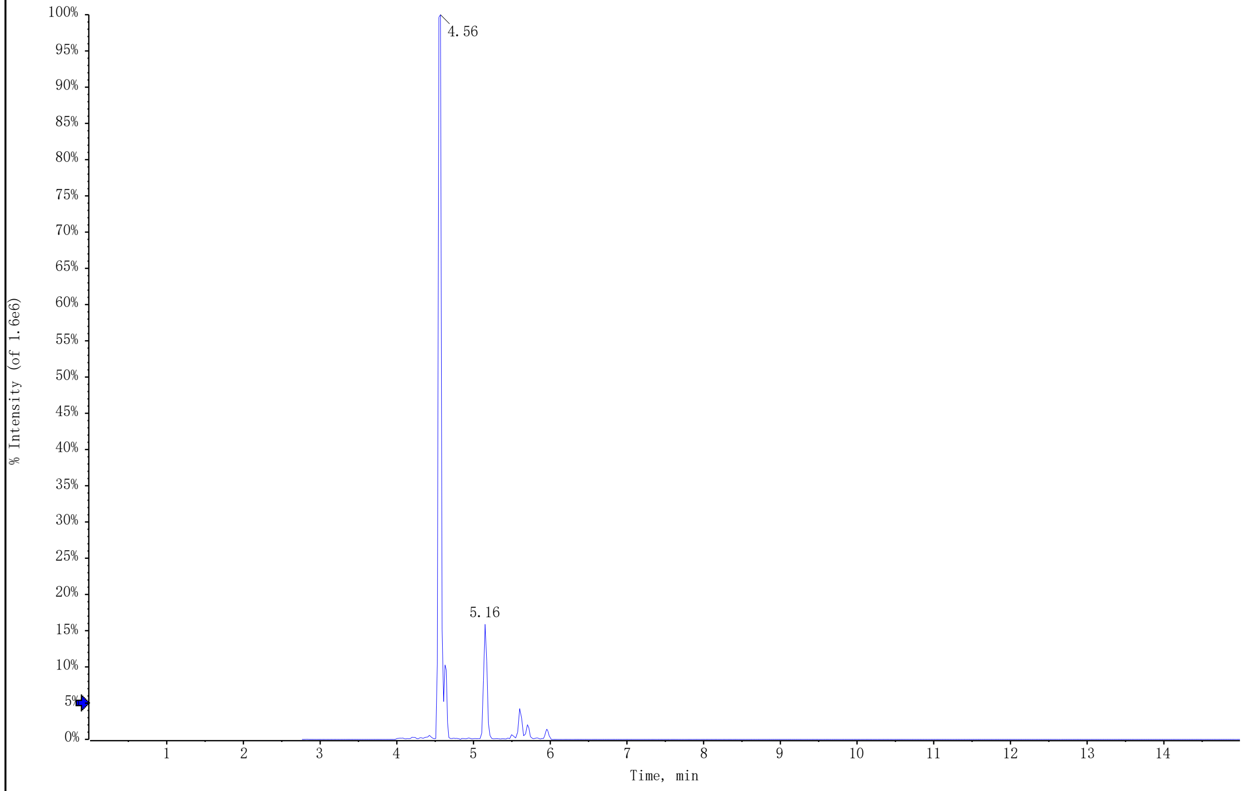

2023/5/9 22:34:22

XIC from CK-2.wiff (sample 1) - CK-2, Experiment 1, +MRM (41 transitions): POS_MSTD0447_q (449.0 / 287.0)

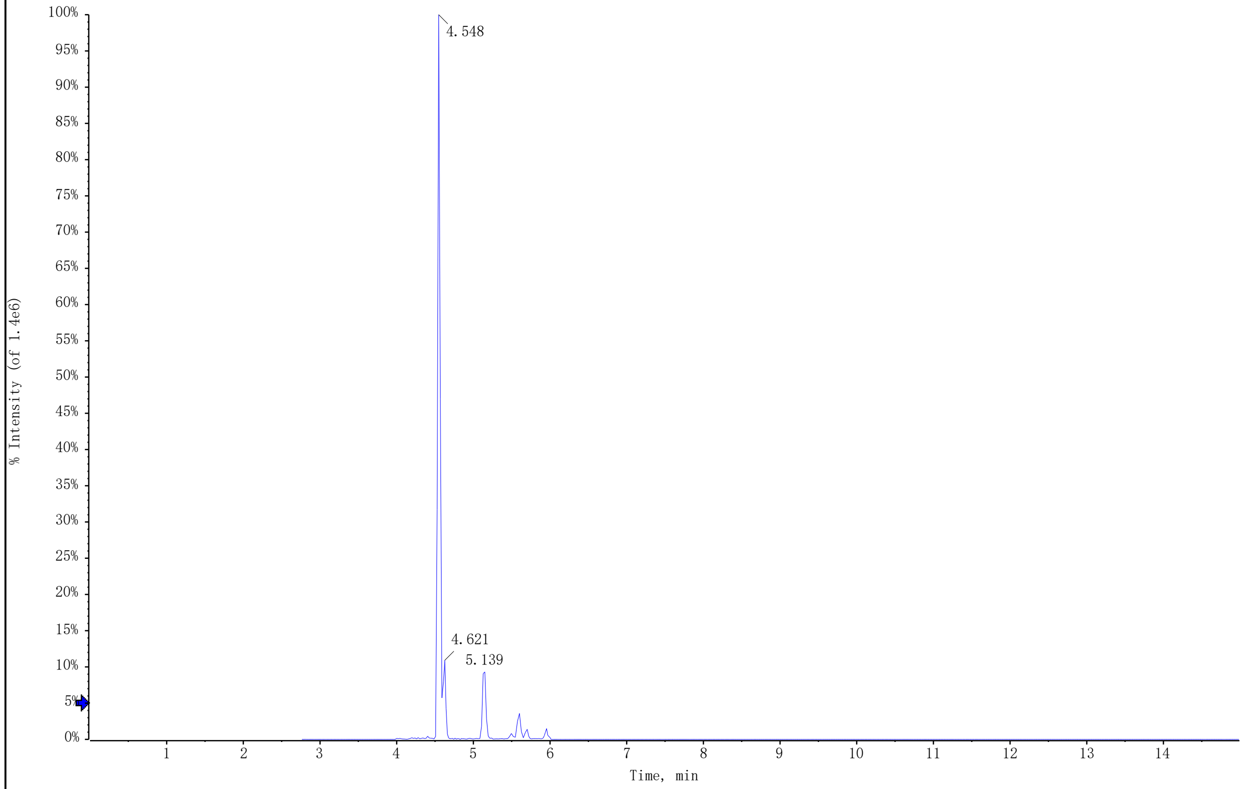

2023/5/10 15:05:21

XIC from CK-3.wiff (sample 1) - CK-3, Experiment 1, +MRM (41 transitions): POS_MSTD0447_q (449.0 / 287.0)

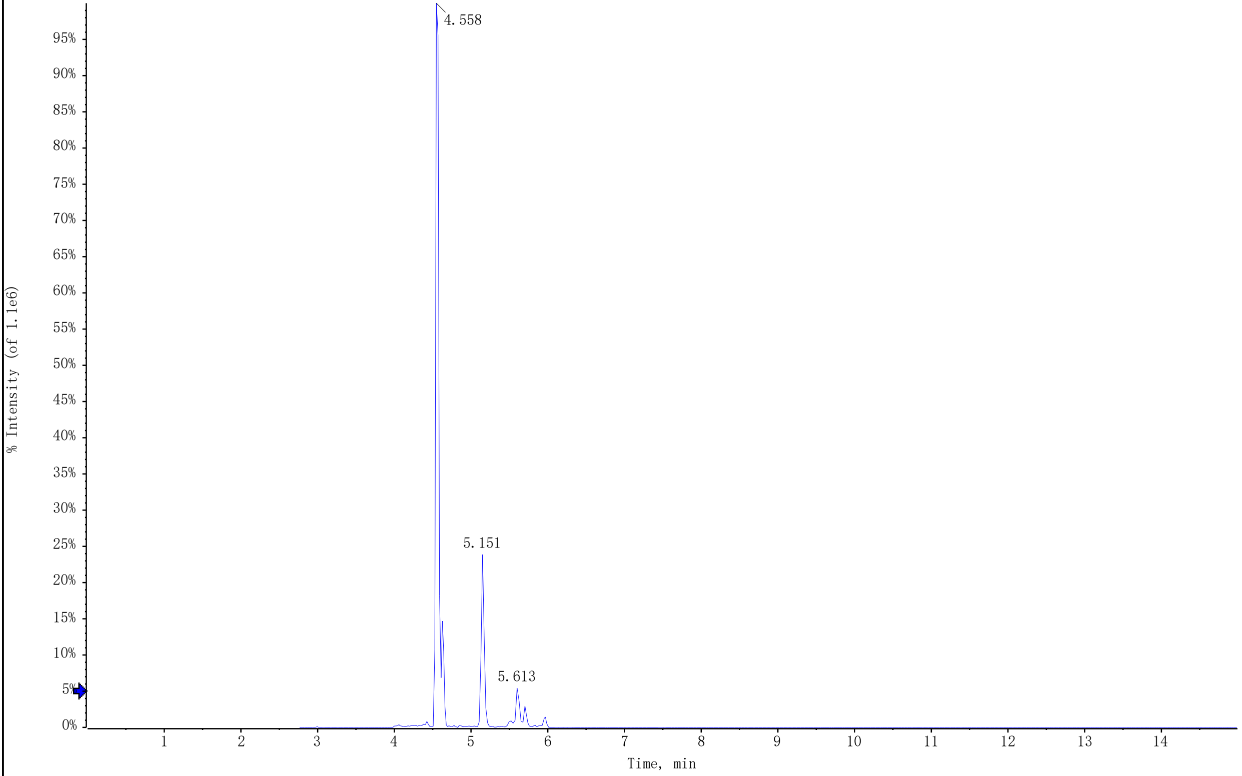

2023/5/10 15:26:45

XIC from CK-4.wiff (sample 1) - CK-4, Experiment 1, +MRM (41 transitions): POS_MSTD0447_q (449.0 / 287.0)

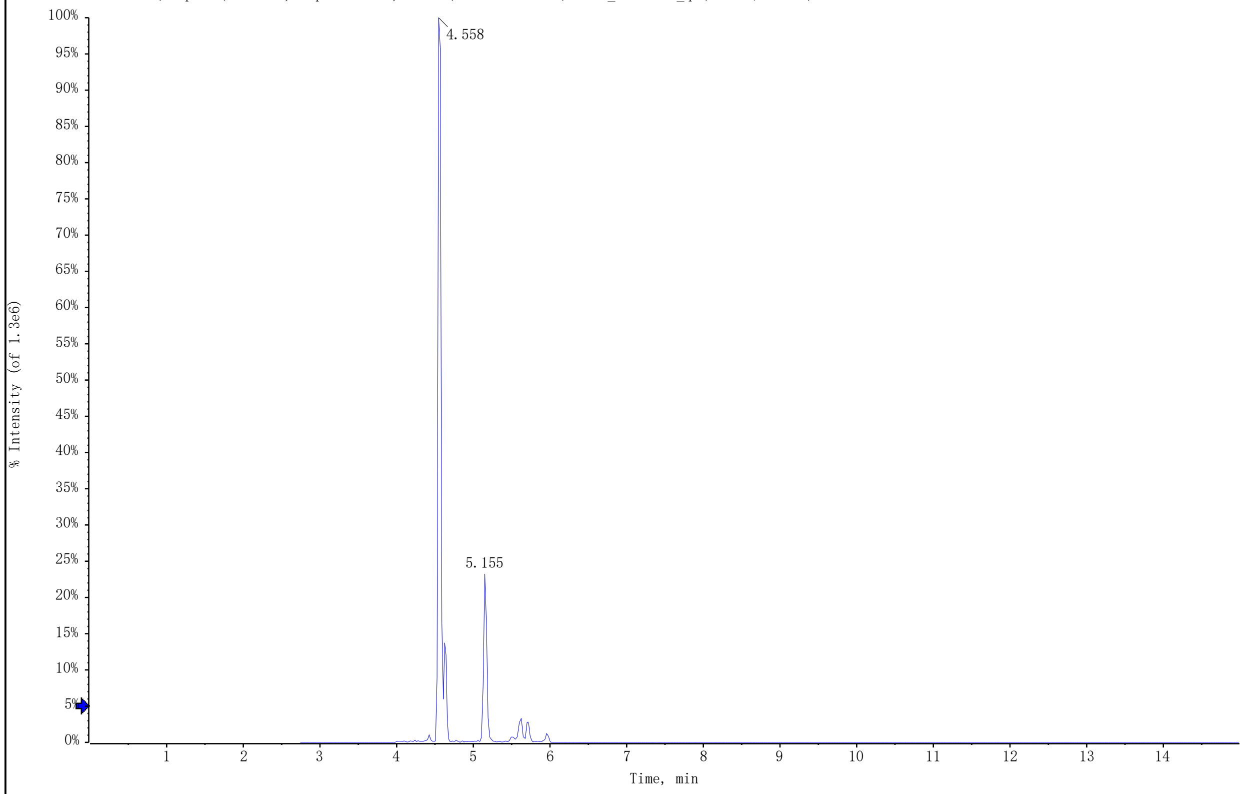

2023/5/10 15:49:17

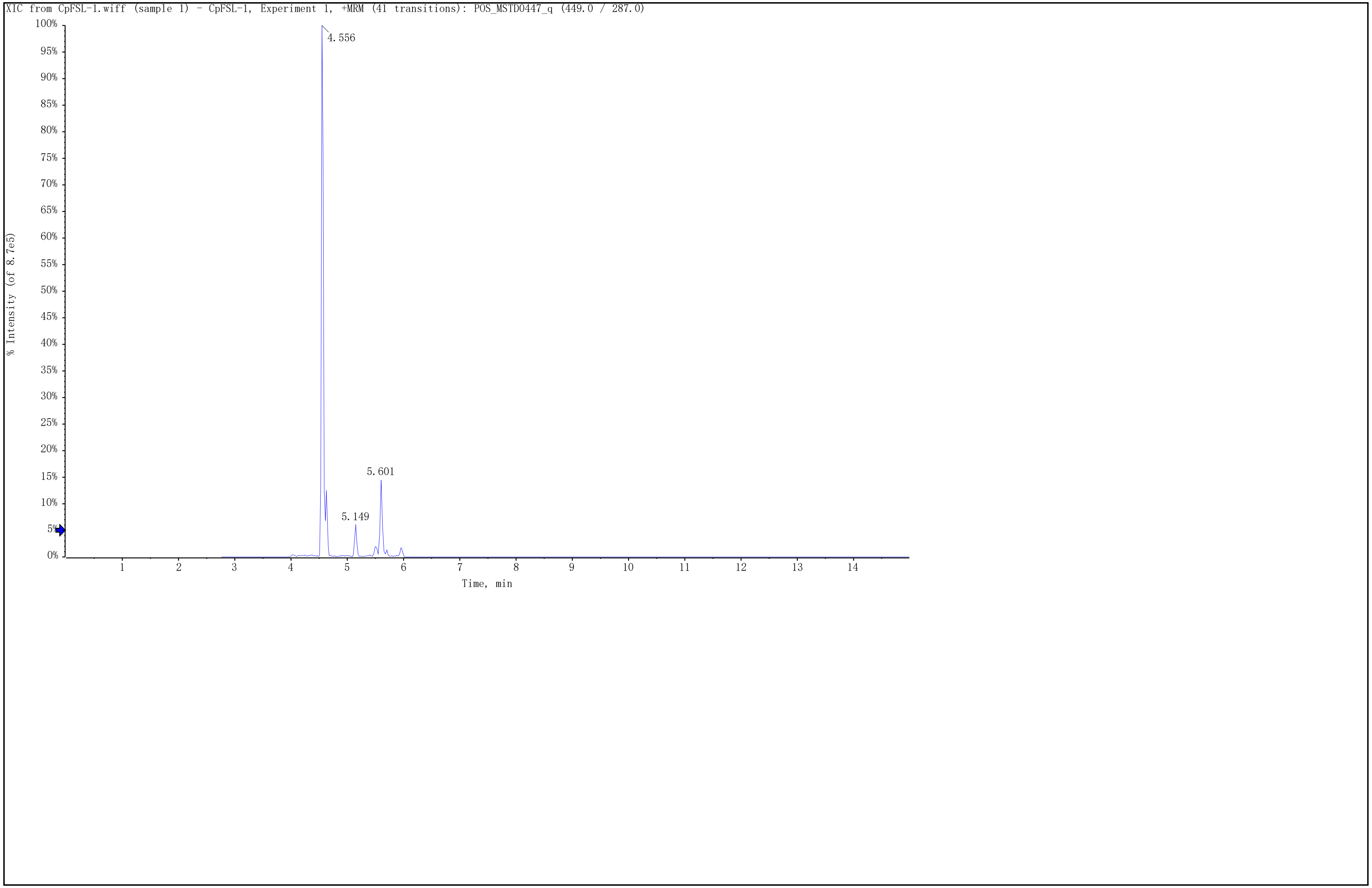

2023/5/10 16:12:42

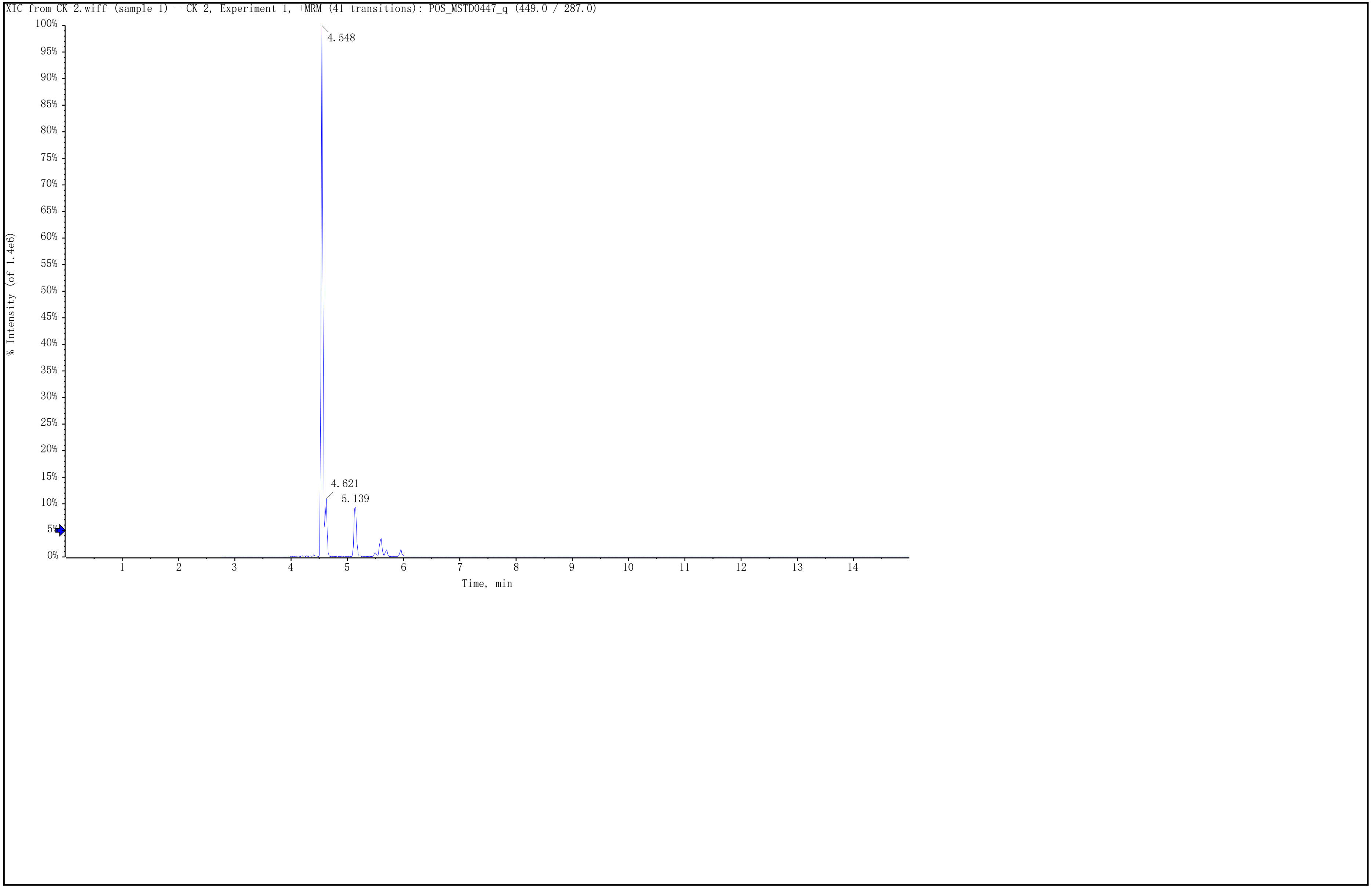

2023/5/10 15:05:21

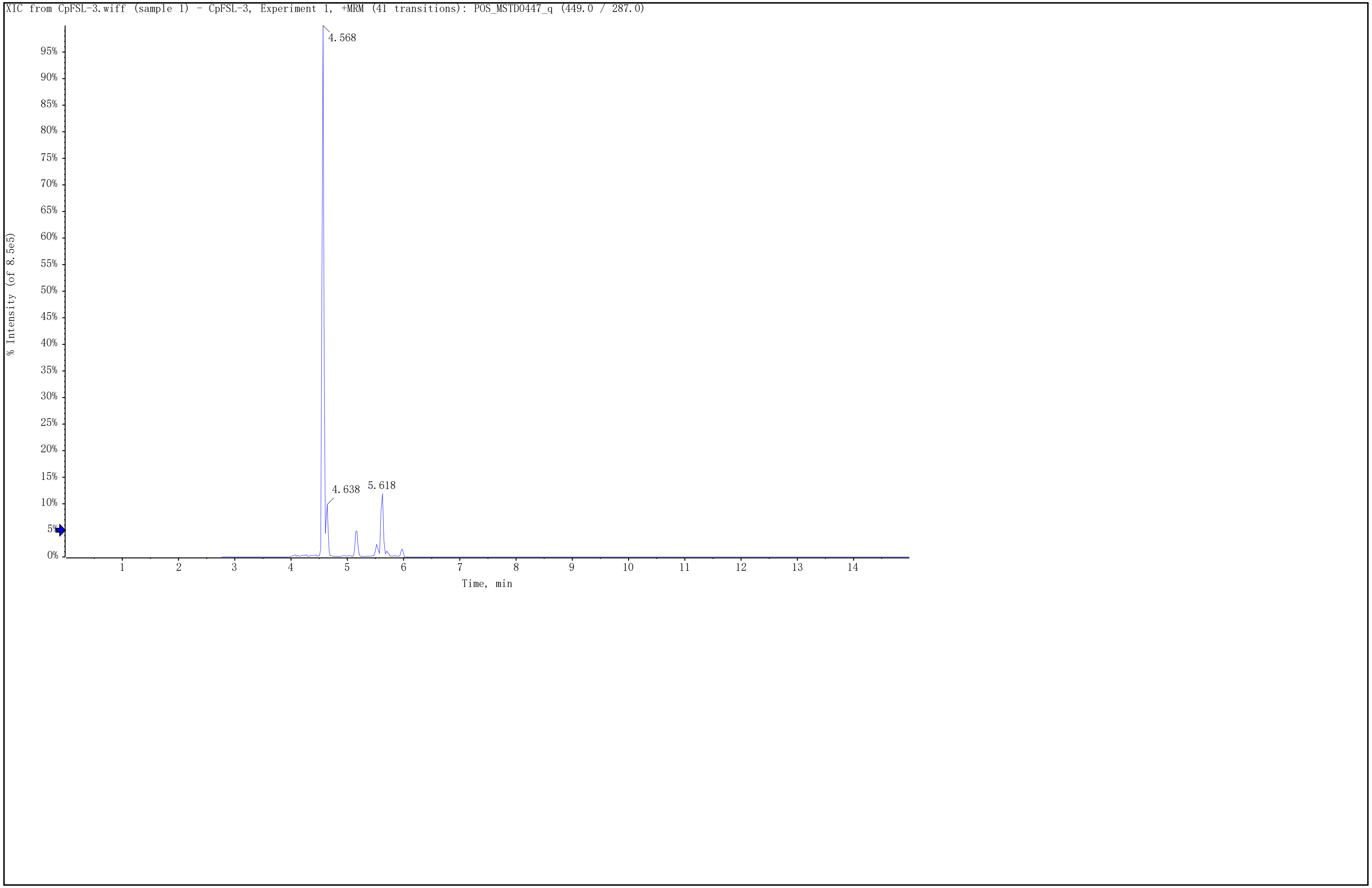

2023/5/10 22:32:51

XIC from CpFSL-4.wiff (sample 1) - CpFSL-4, Experiment 1, +MRM (41 transitions): POS_MSTD0447_q (449.0 / 287.0)

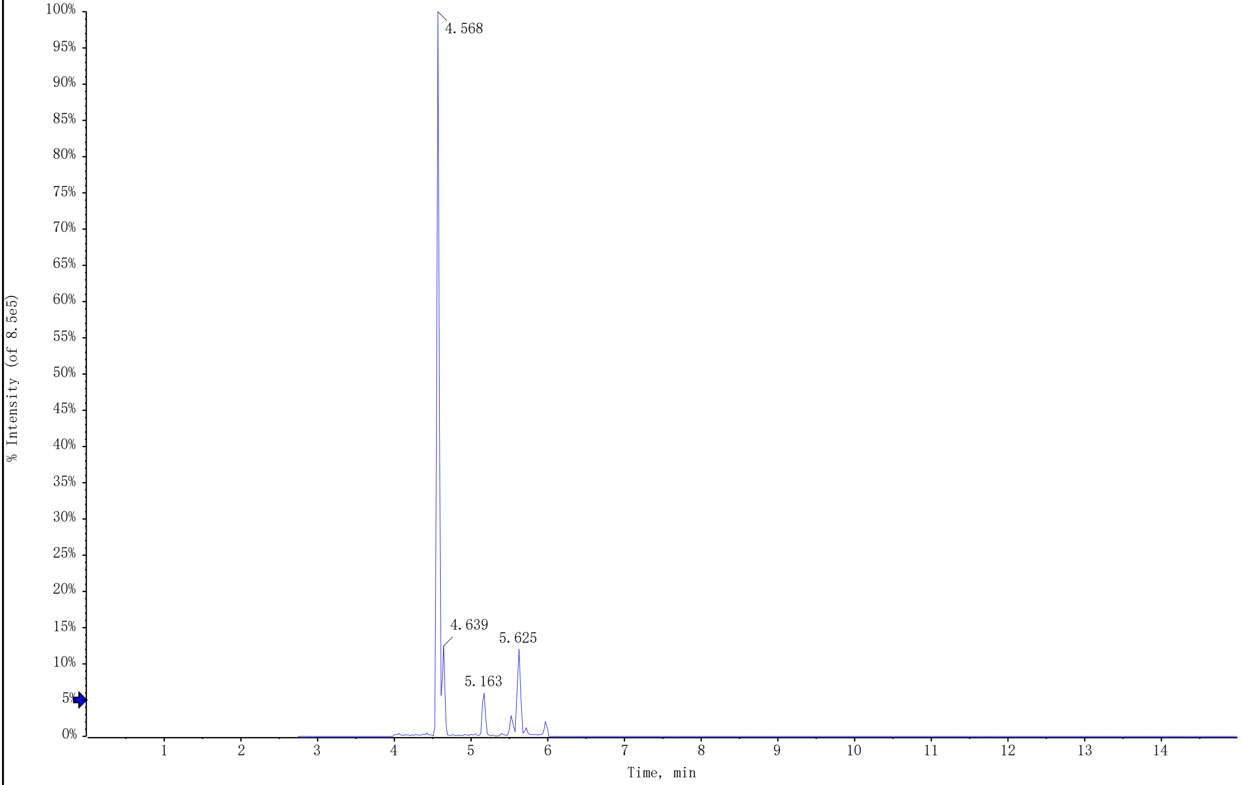

2023/5/11 14:43:05

XIC from CK-1.wiff (sample 1) - CK-1, Experiment 1, +MRM (41 transitions): POS_MSTD0430_q (447.0 / 285.0)

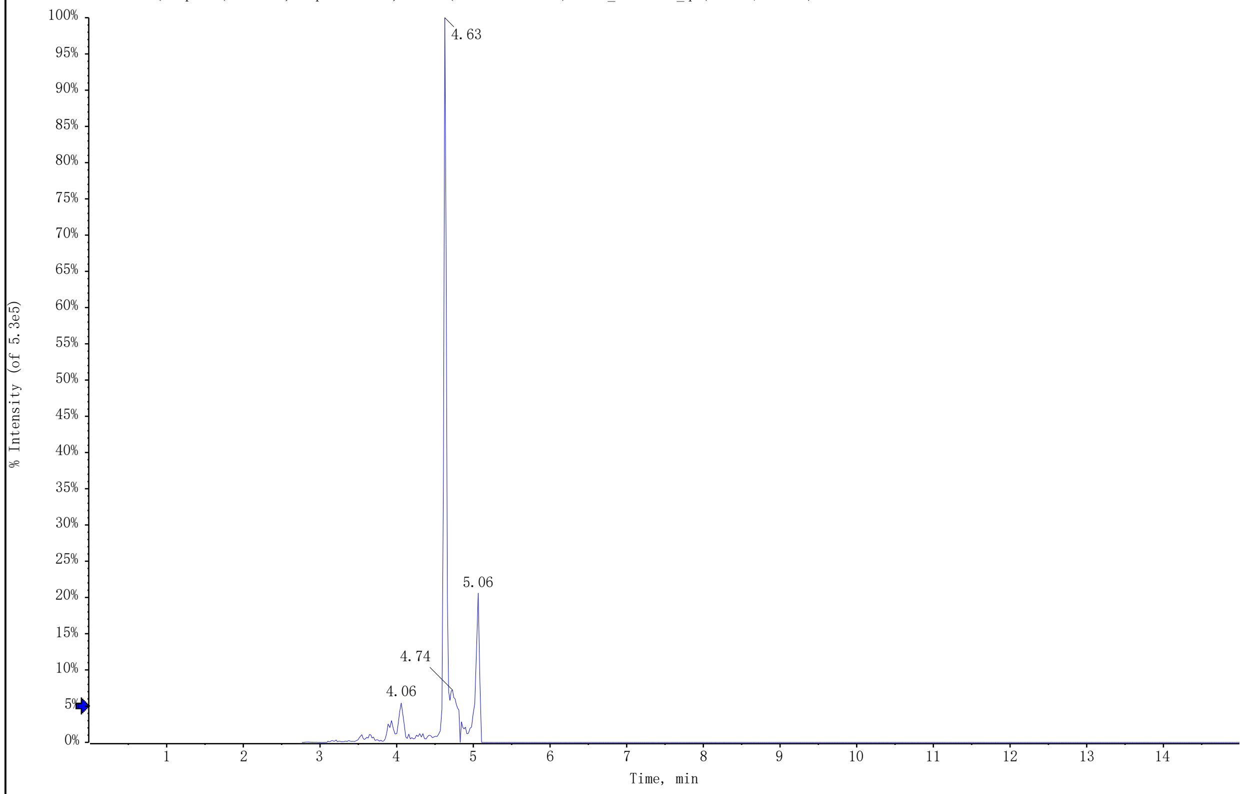

2023/5/9 22:19:25

XIC from CK-2.wiff (sample 1) - CK-2, Experiment 1, +MRM (41 transitions): POS_MSTD0430_q (447.0 / 285.0)

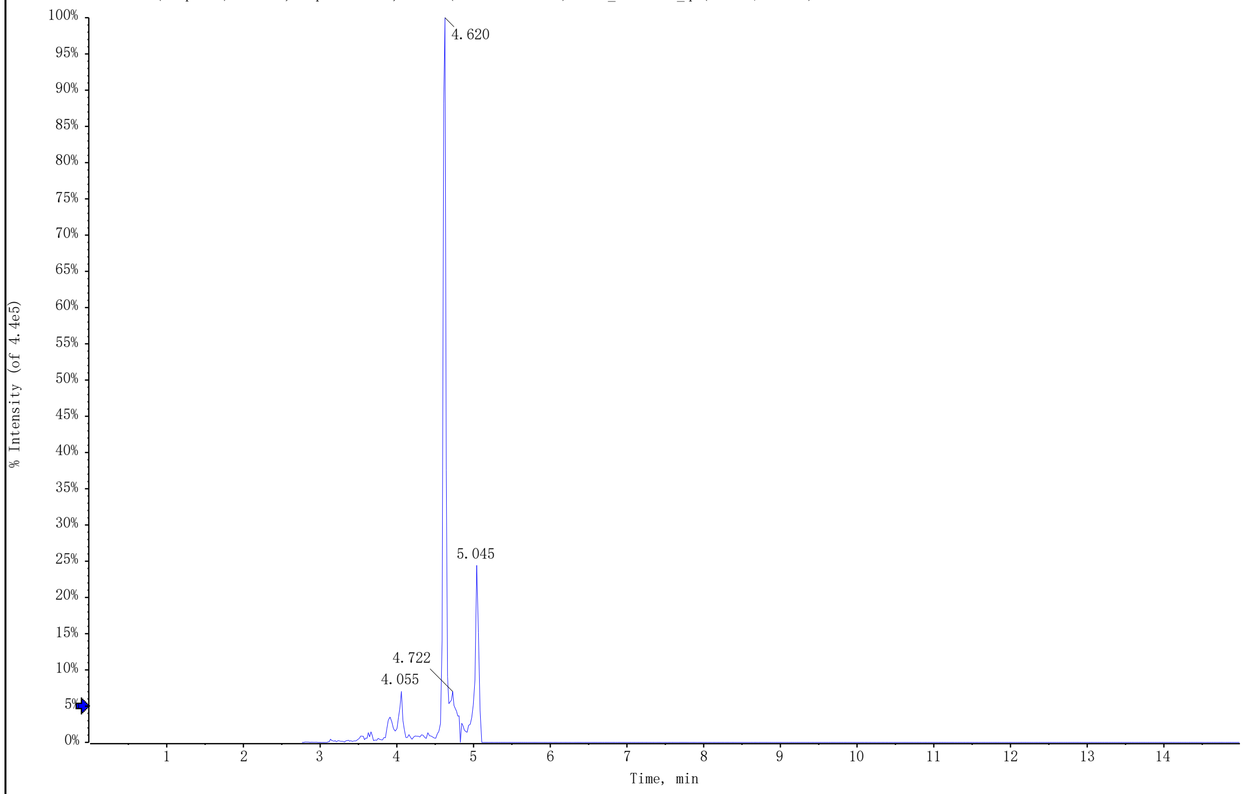

2023/5/10 15:01:53

XIC from CK-3.wiff (sample 1) - CK-3, Experiment 1, +MRM (41 transitions): POS_MSTD0430_q (447.0 / 285.0)

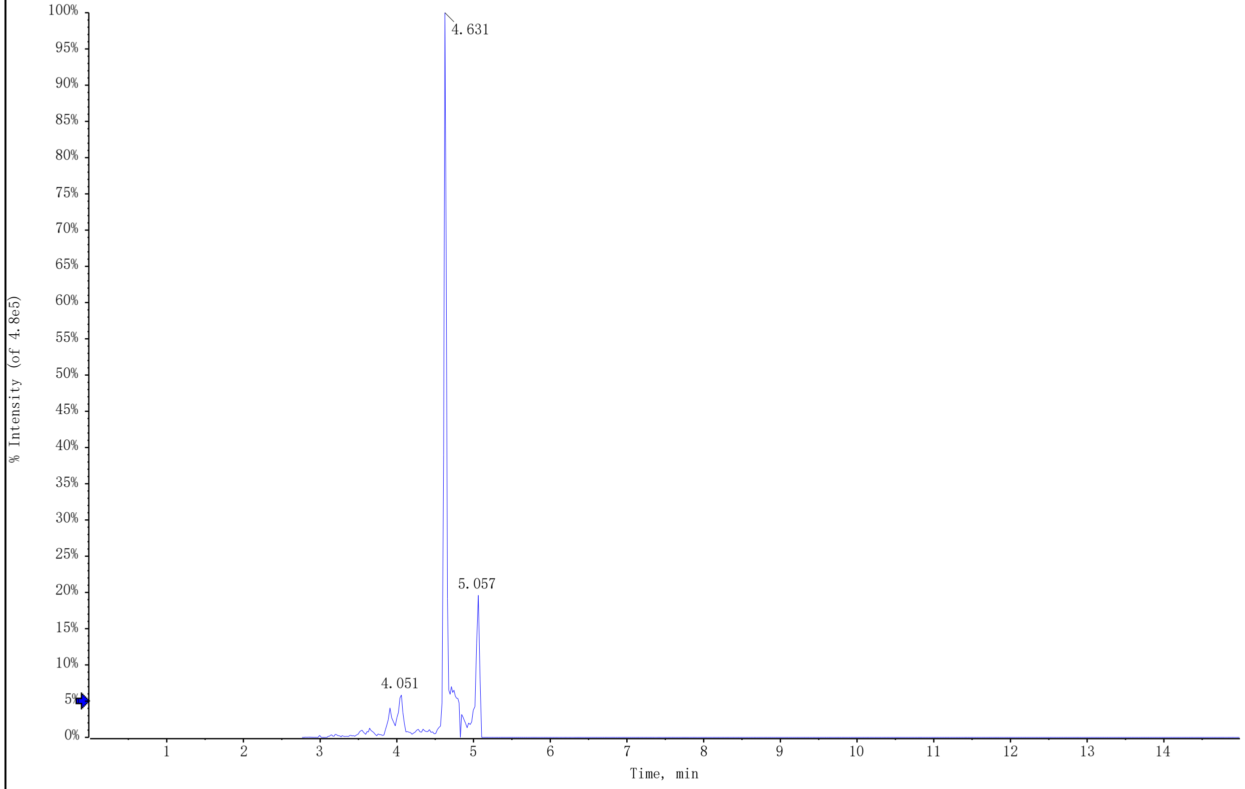

2023/5/10 15:22:33

XIC from CK-4.wiff (sample 1) - CK-4, Experiment 1, +MRM (41 transitions): POS_MSTD0430_q (447.0 / 285.0)

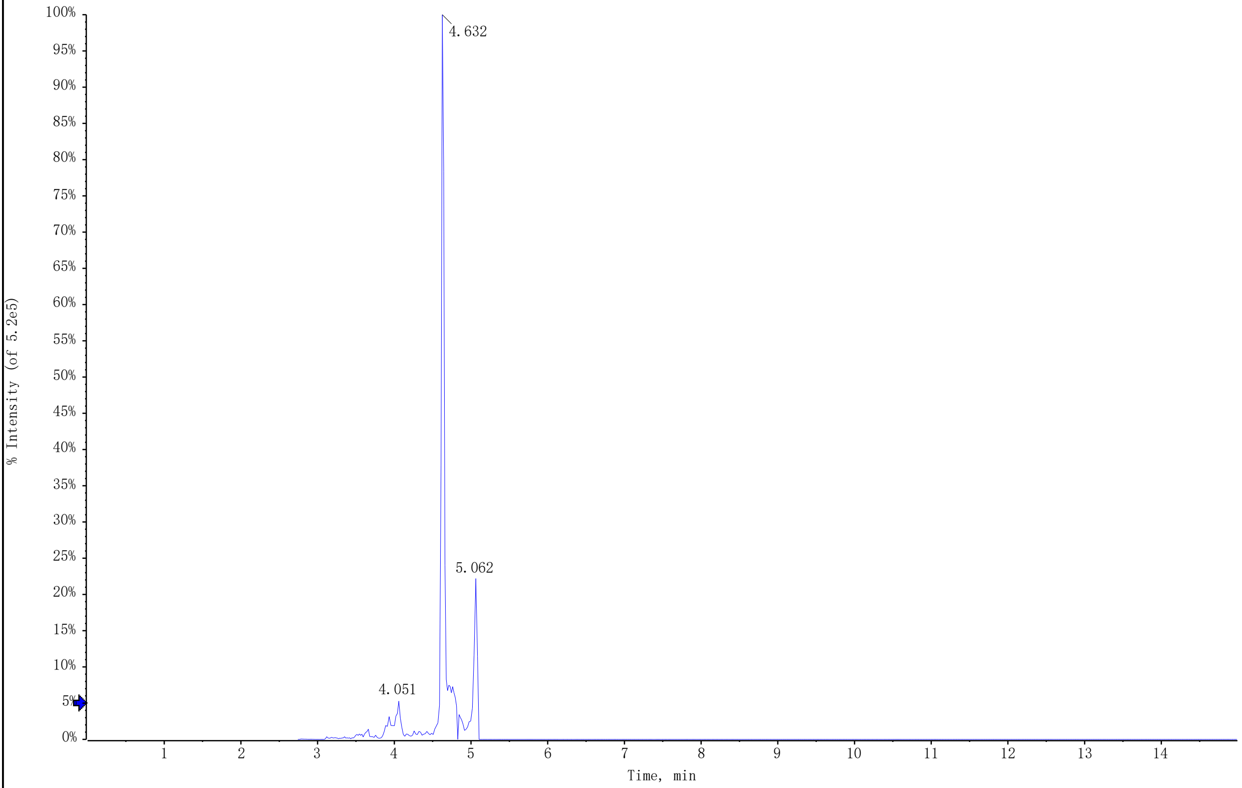

2023/5/10 15:44:06

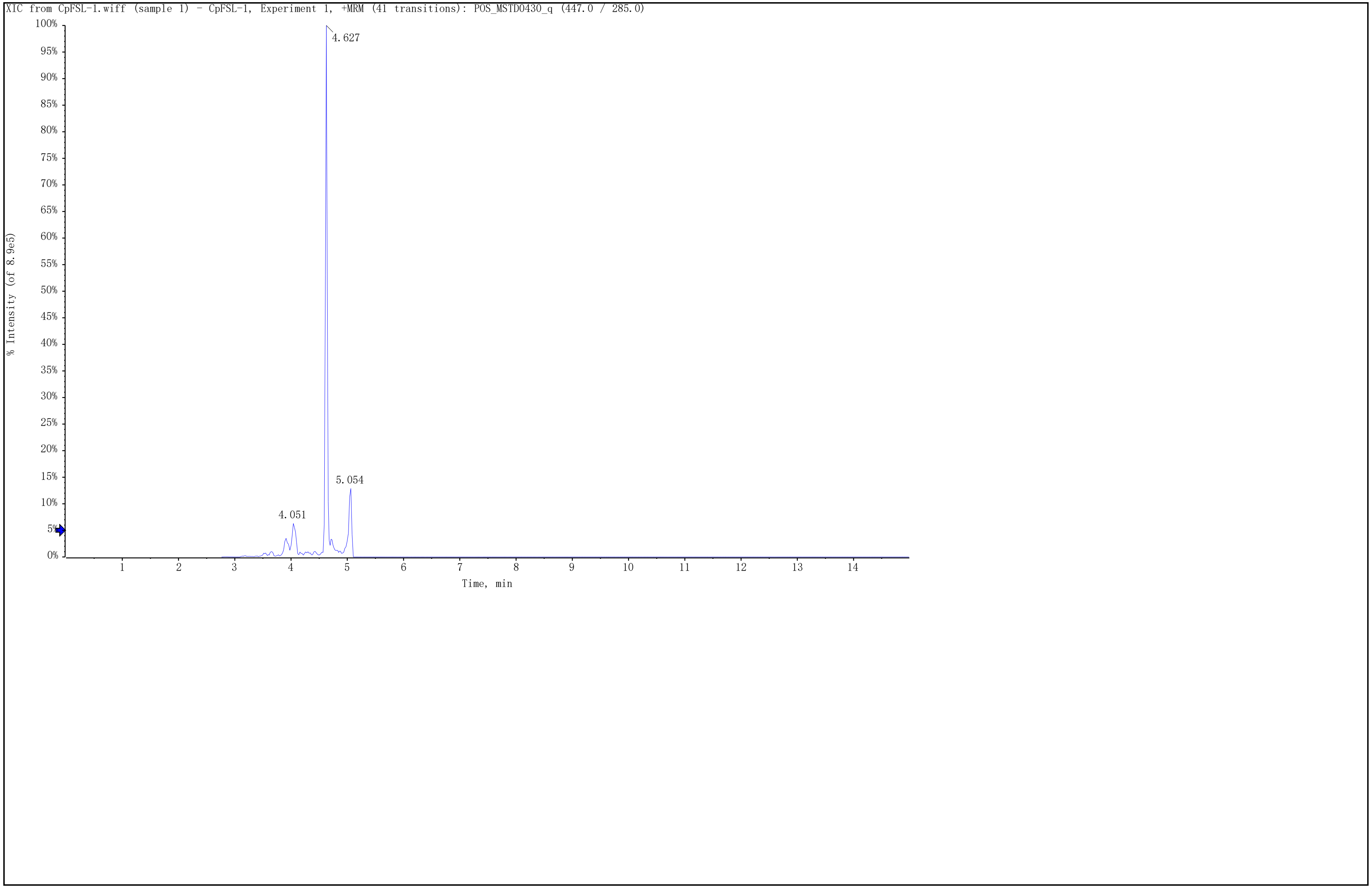

2023/5/10 16:06:45

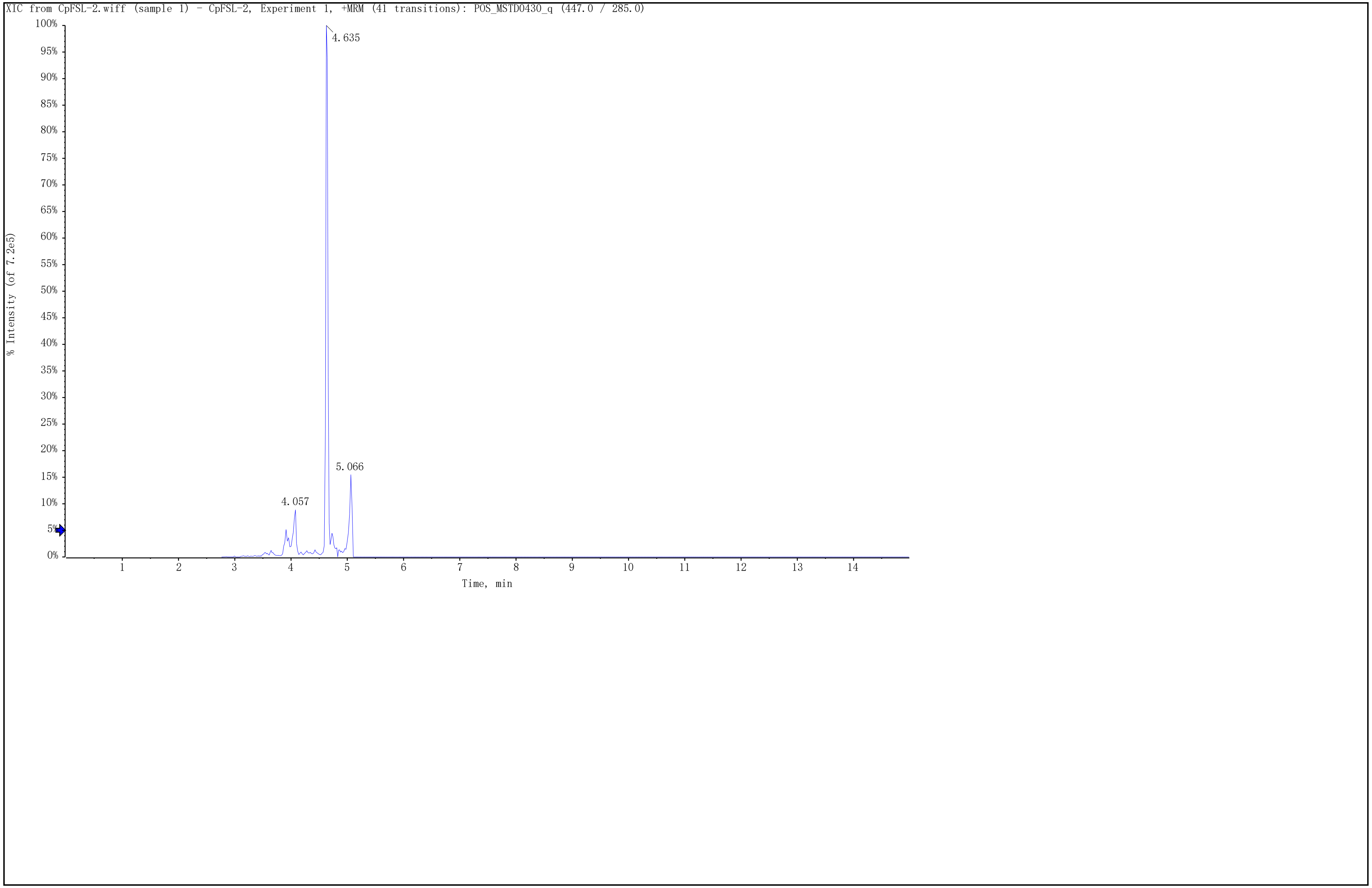

2023/5/10 16:28:06

XIC from CpFSL-3.wiff (sample 1) - CpFSL-3, Experiment 1, +MRM (41 transitions): POS_MSTD0430_q (447.0 / 285.0)

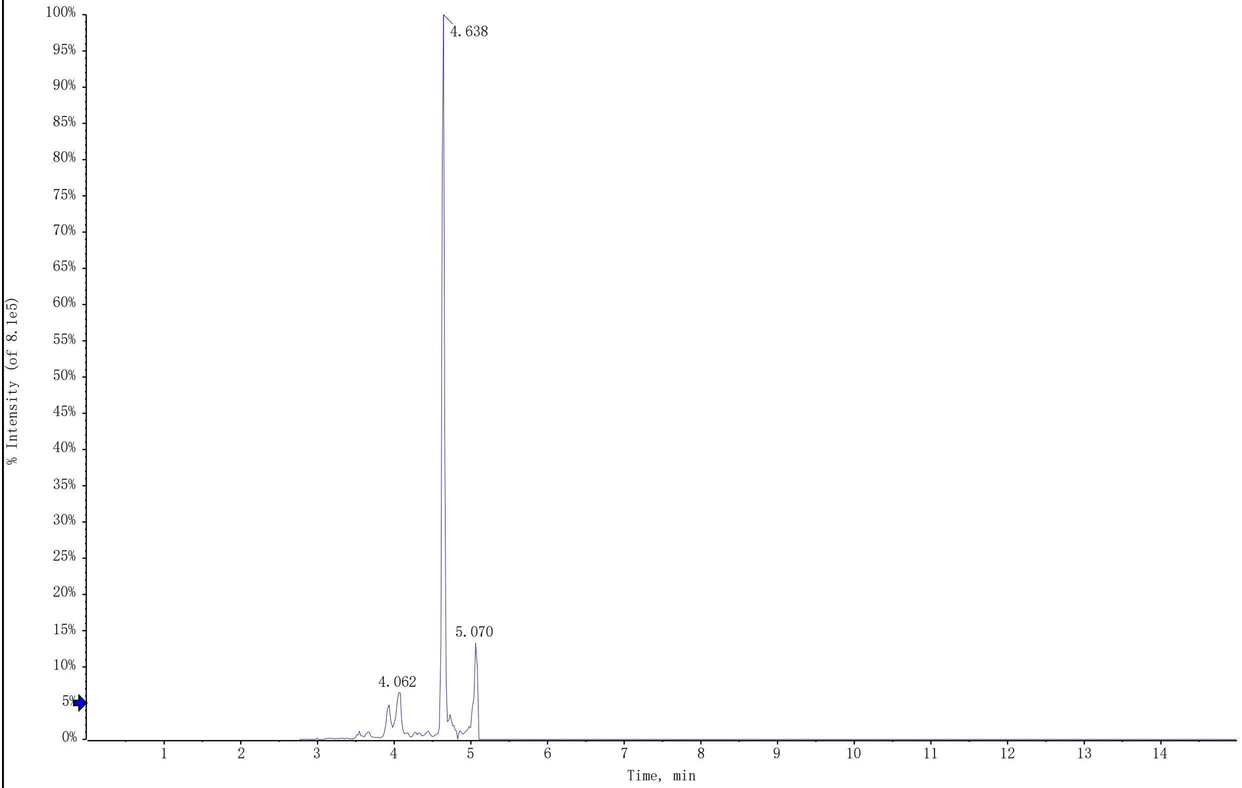

2023/5/10 22:27:17

XIC from CpFSL-4.wiff (sample 1) - CpFSL-4, Experiment 1, +MRM (41 transitions): POS_MSTD0430_q (447.0 / 285.0)

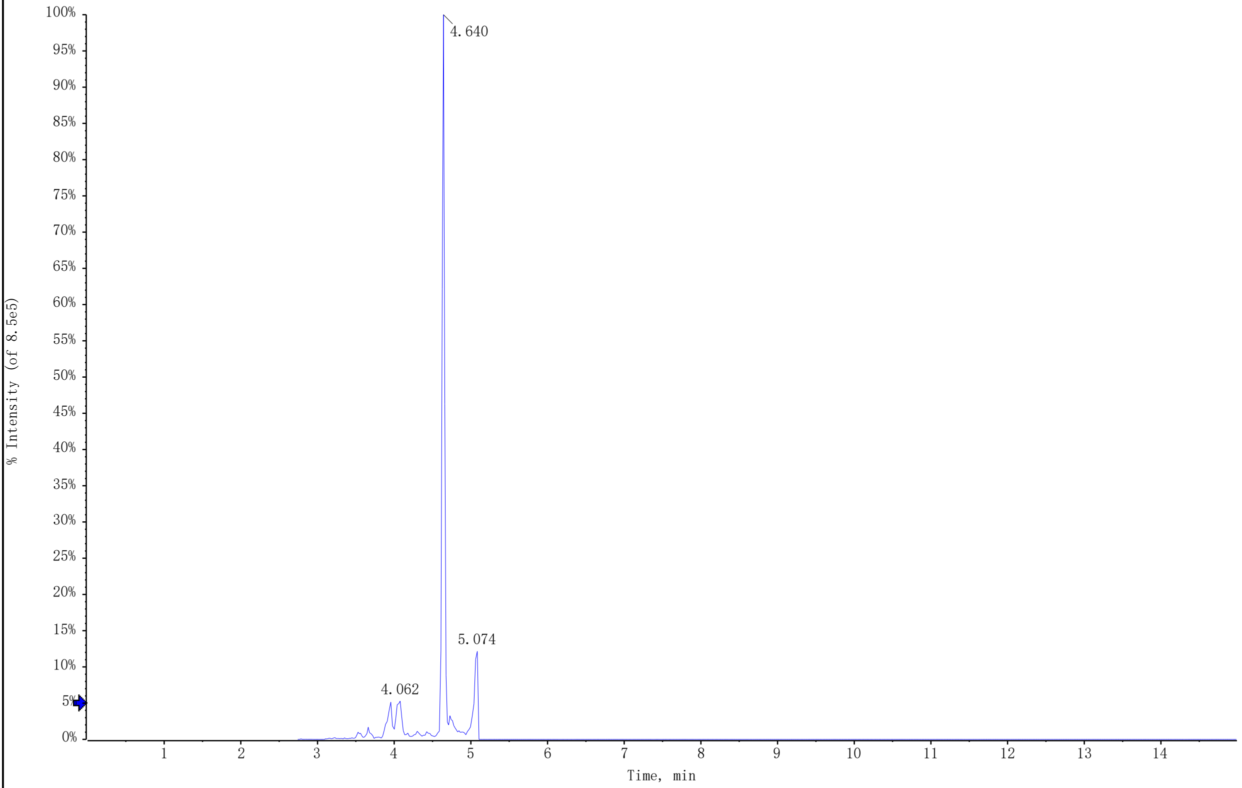

2023/5/11 14:37:06

XIC from CK-2.wiff (sample 1) - CK-2, Experiment 2, -MRM (38 transitions): NEG_MSTD0421_2 (289.0 / 203.0)

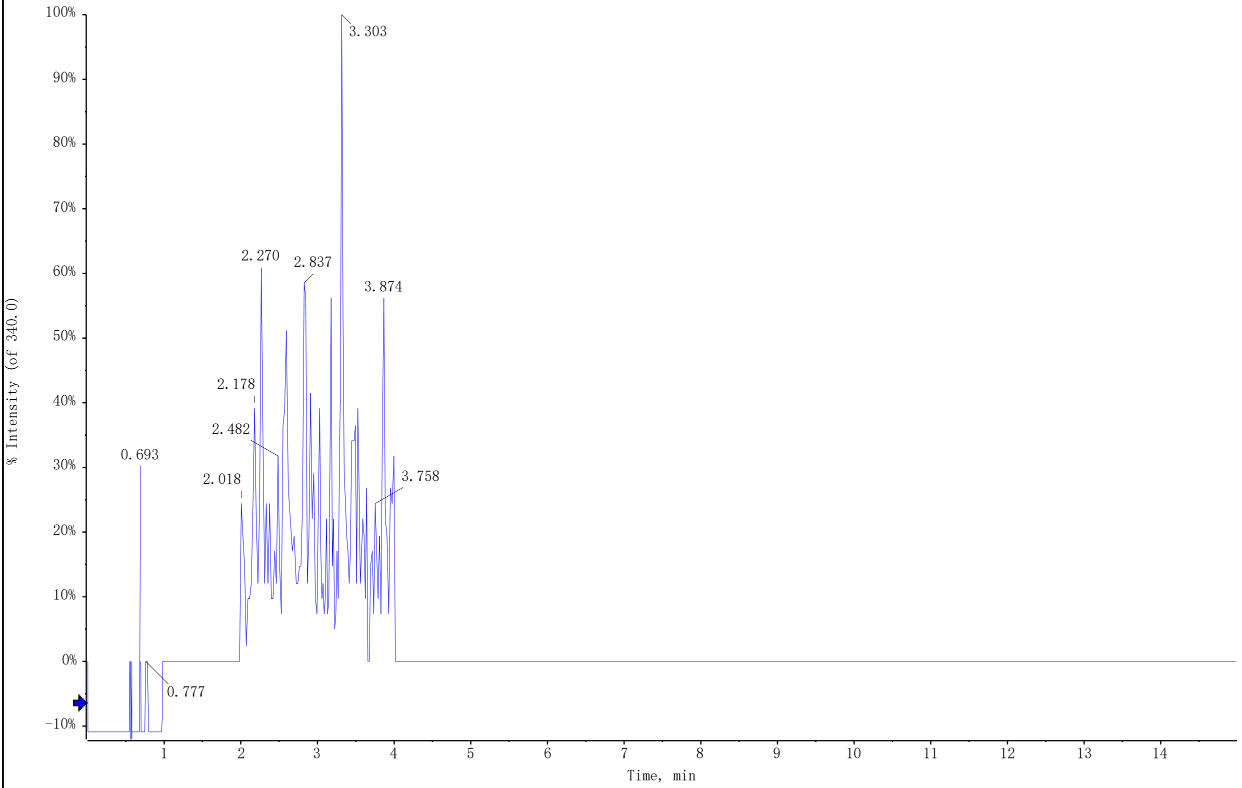

2023/5/10 15:02:58

XIC from CK-3.wiff (sample 1) - CK-3, Experiment 2, -MRM (38 transitions): NEG_MSTD0421_2 (289.0 / 203.0)

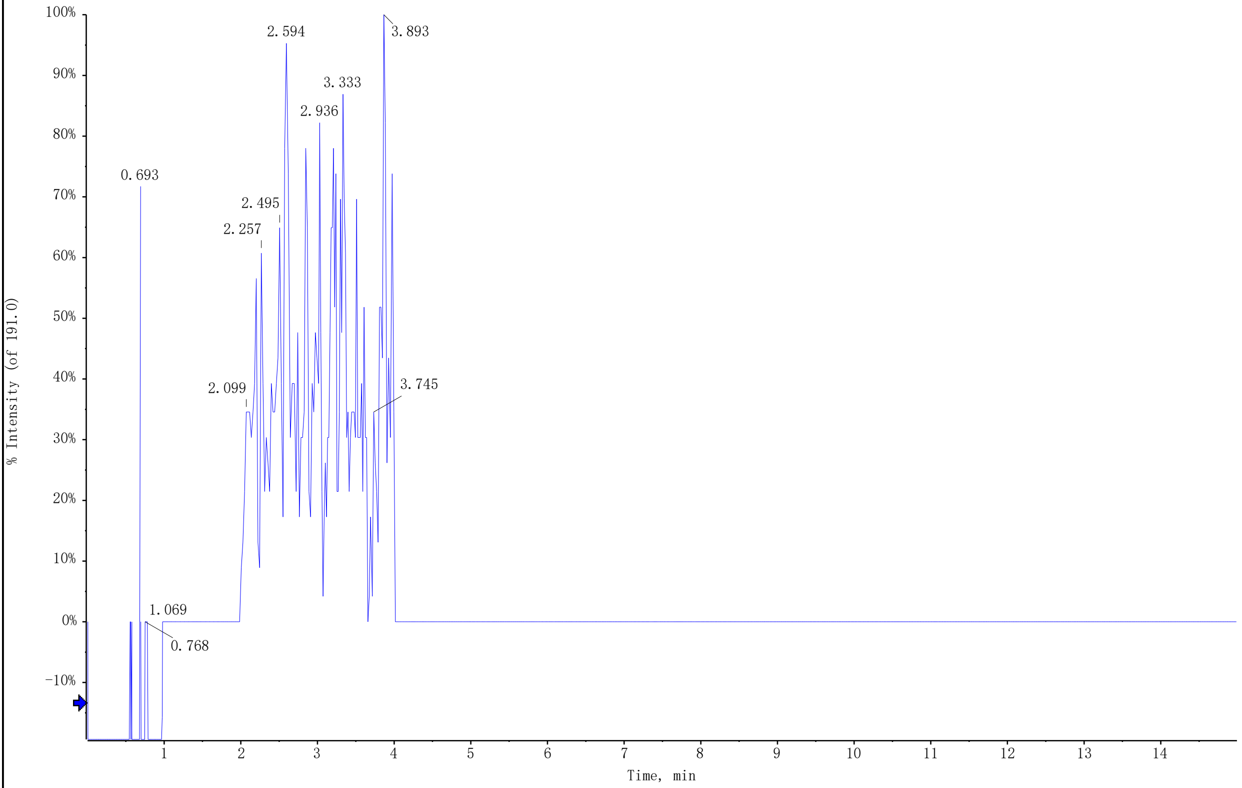

2023/5/10 15:24:02

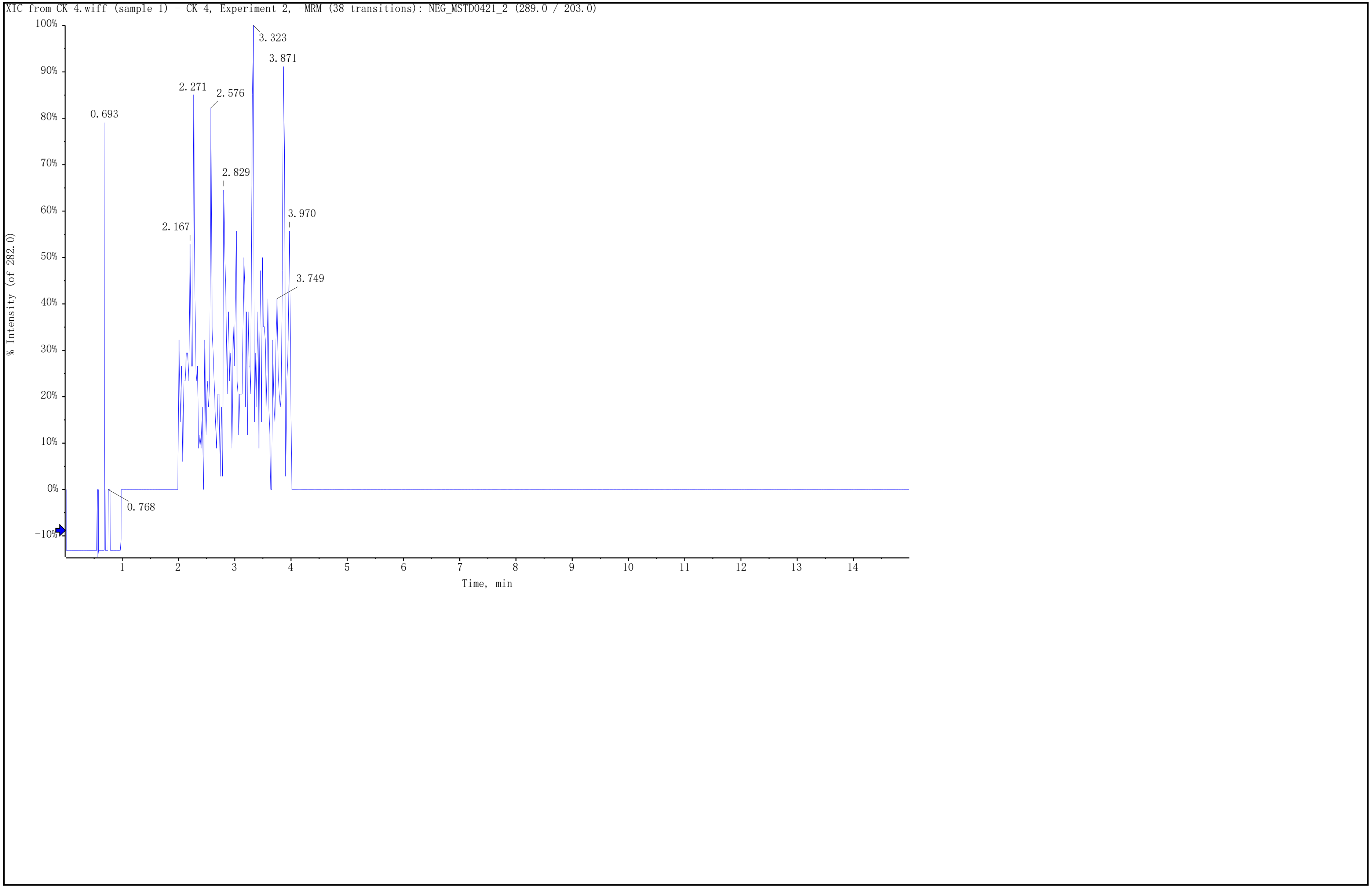

2023/5/10 15:46:33

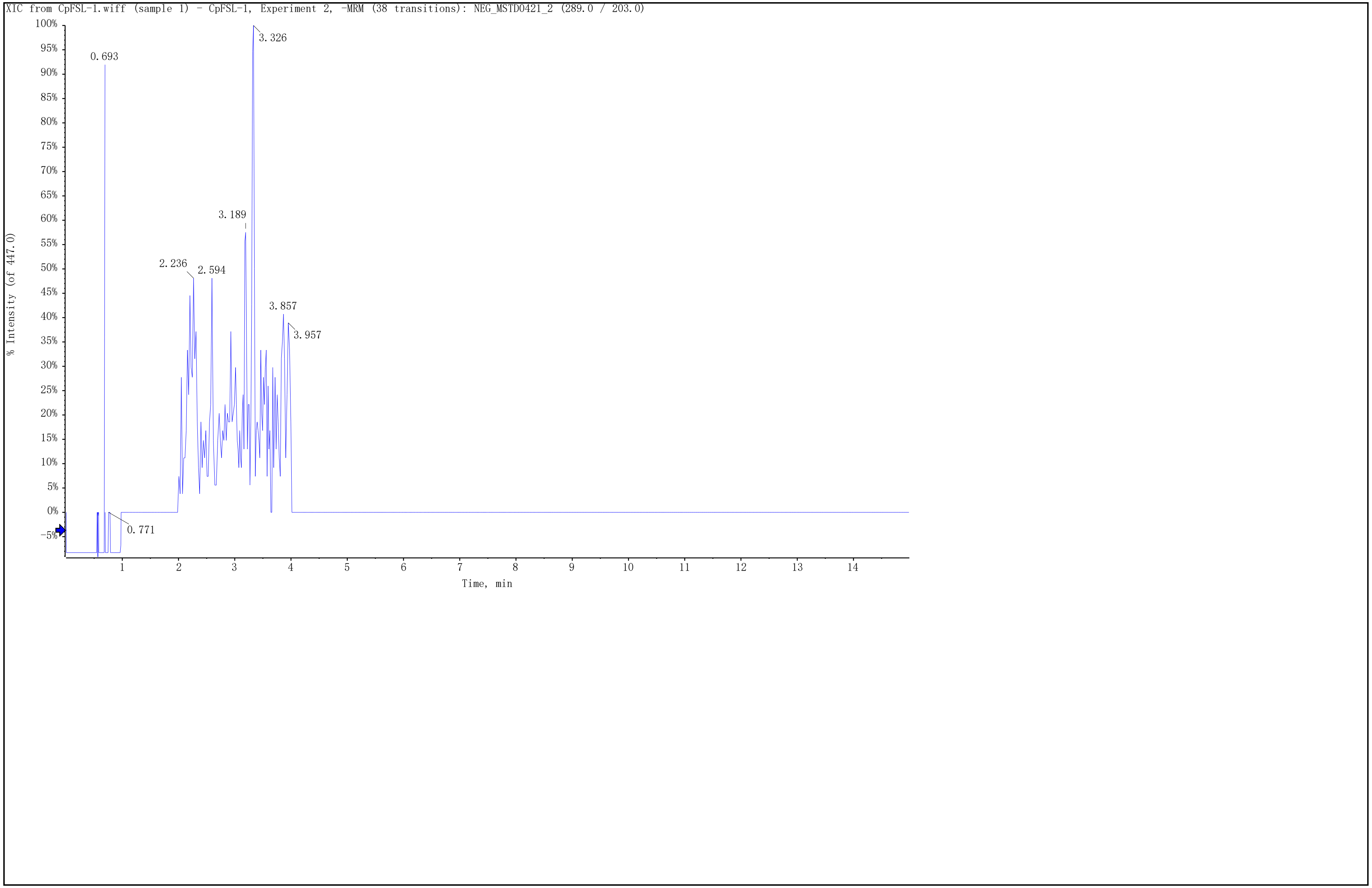

2023/5/10 16:09:33

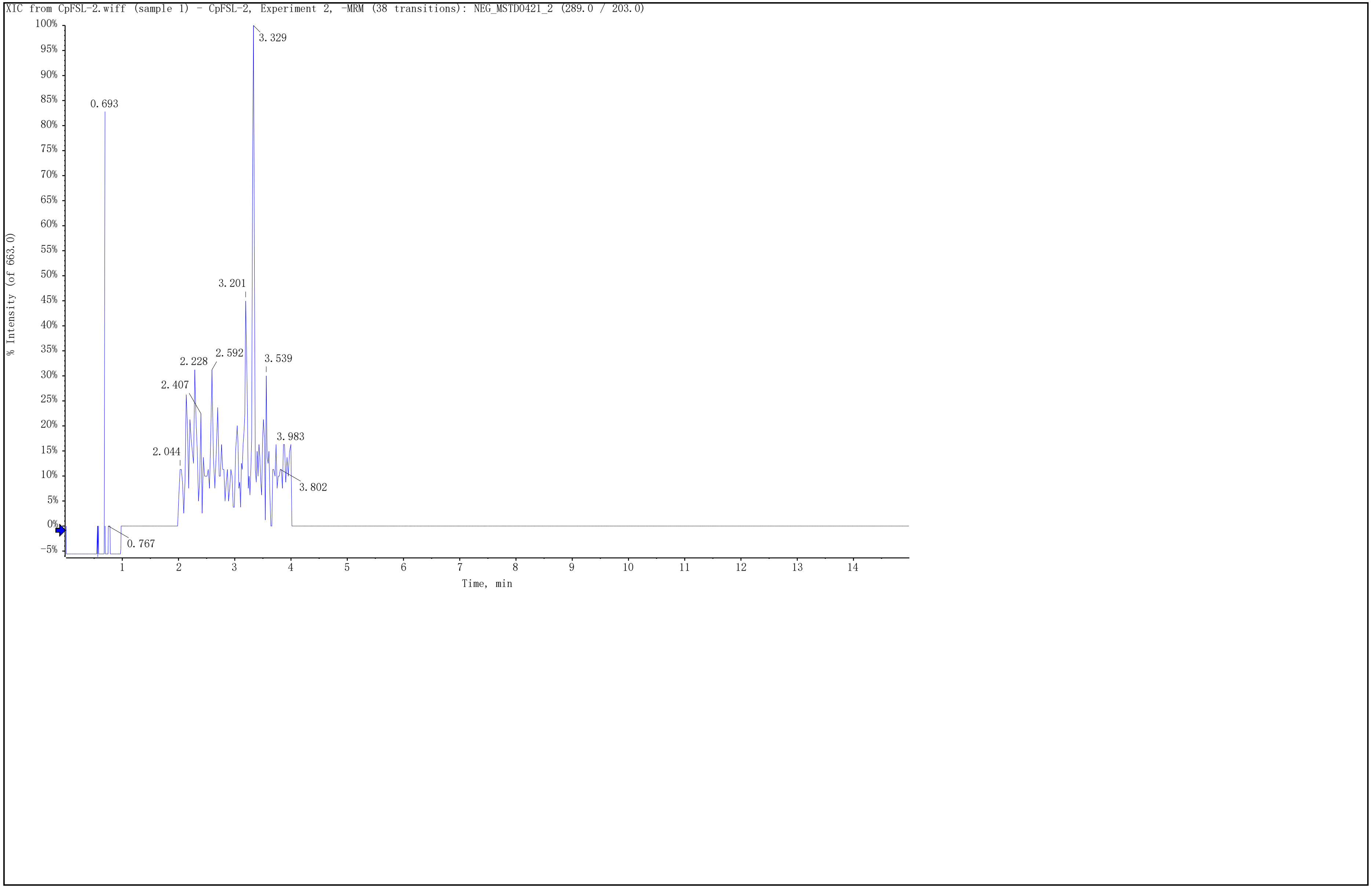

2023/5/10 16:29:18

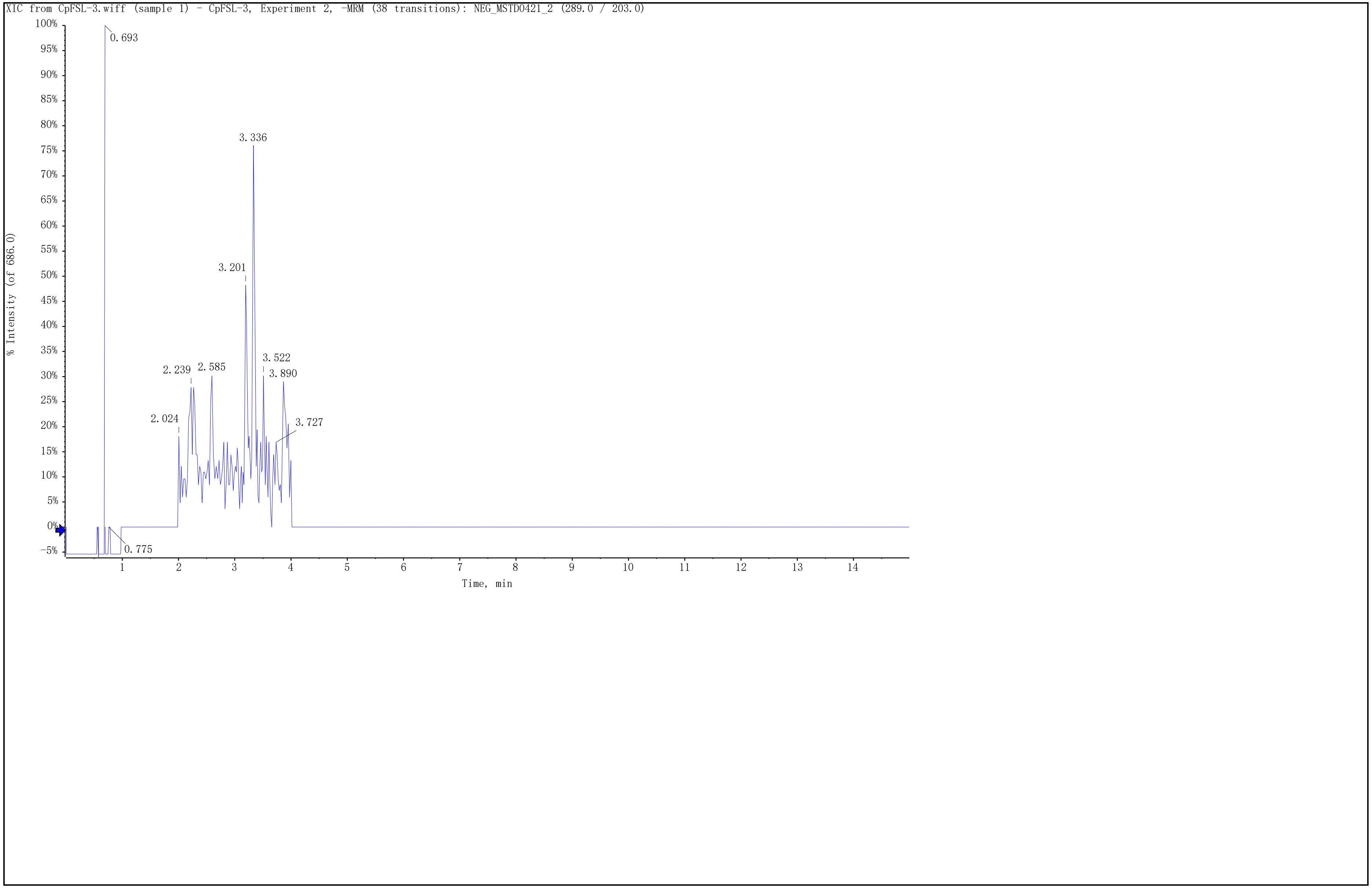

2023/5/10 22:28:45

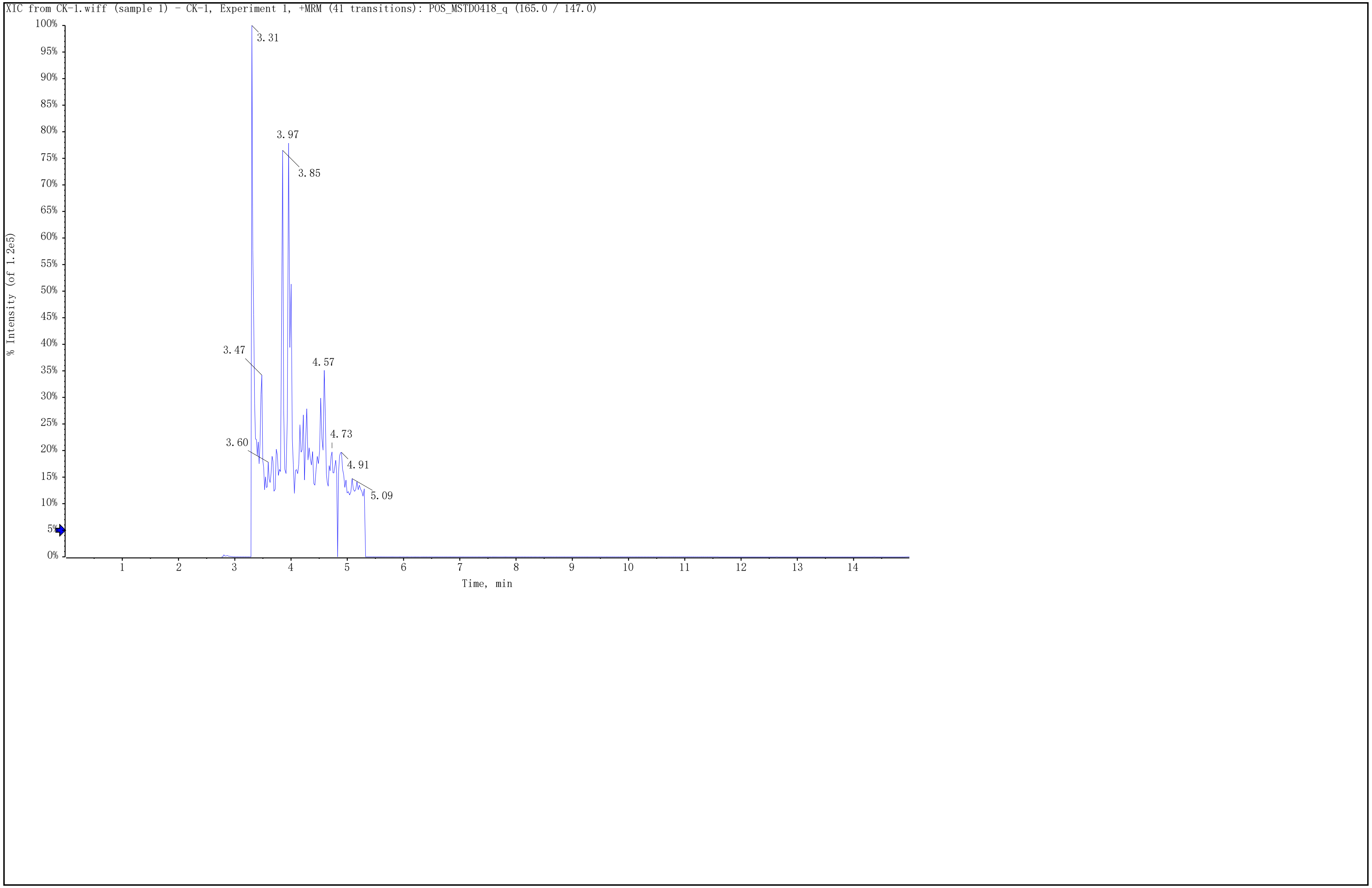

2023/5/9 22:41:38

XIC from CK-2.wiff (sample 1) - CK-2, Experiment 1, +MRM (41 transitions): POS_MSTD0418_q (165.0 / 147.0)

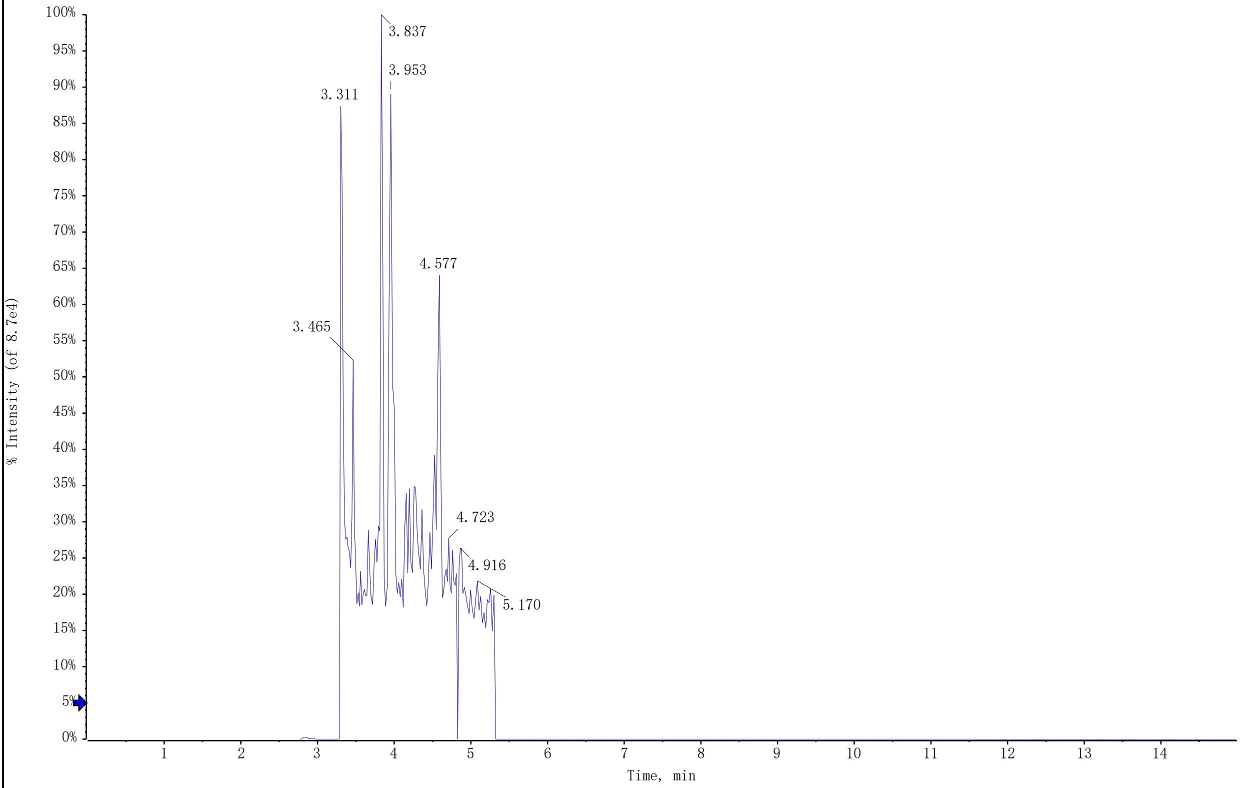

2023/5/10 15:08:31

XIC from CK-3.wiff (sample 1) - CK-3, Experiment 1, +MRM (41 transitions): POS_MSTD0418_q (165.0 / 147.0)

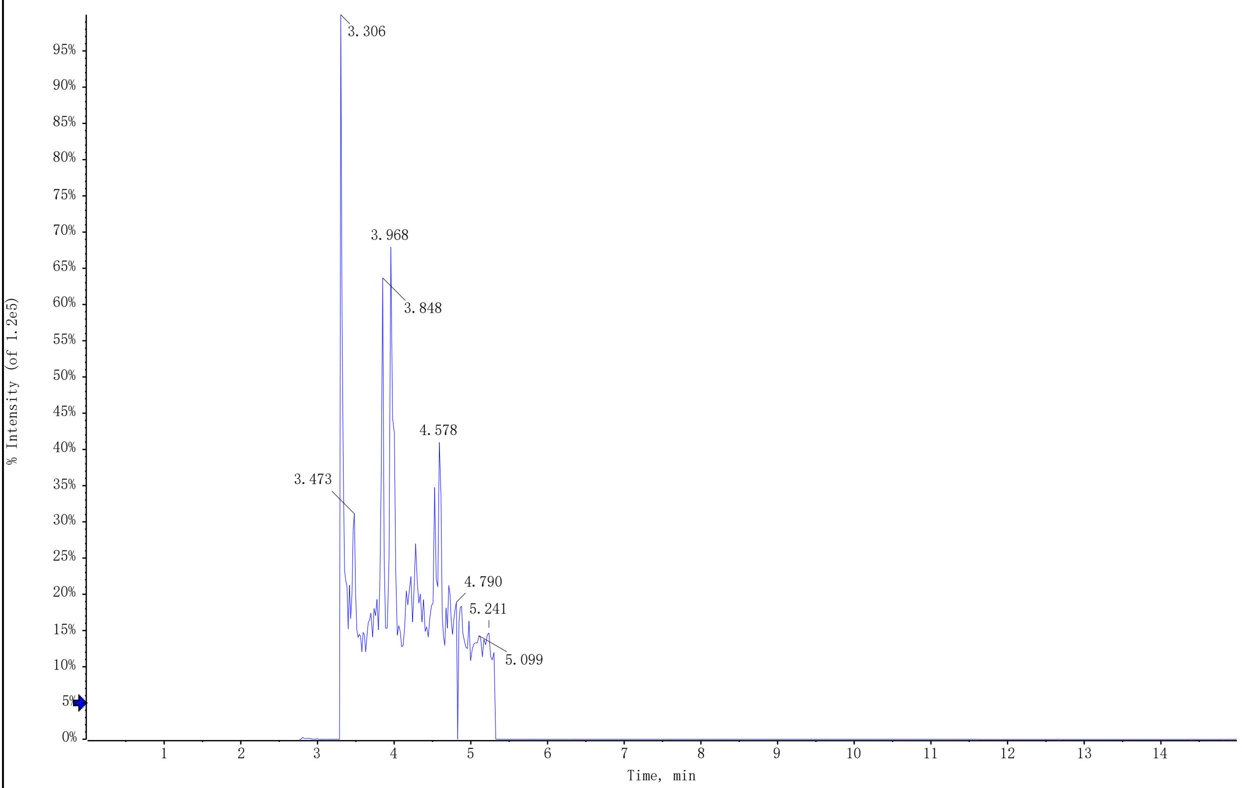

2023/5/10 15:38:49

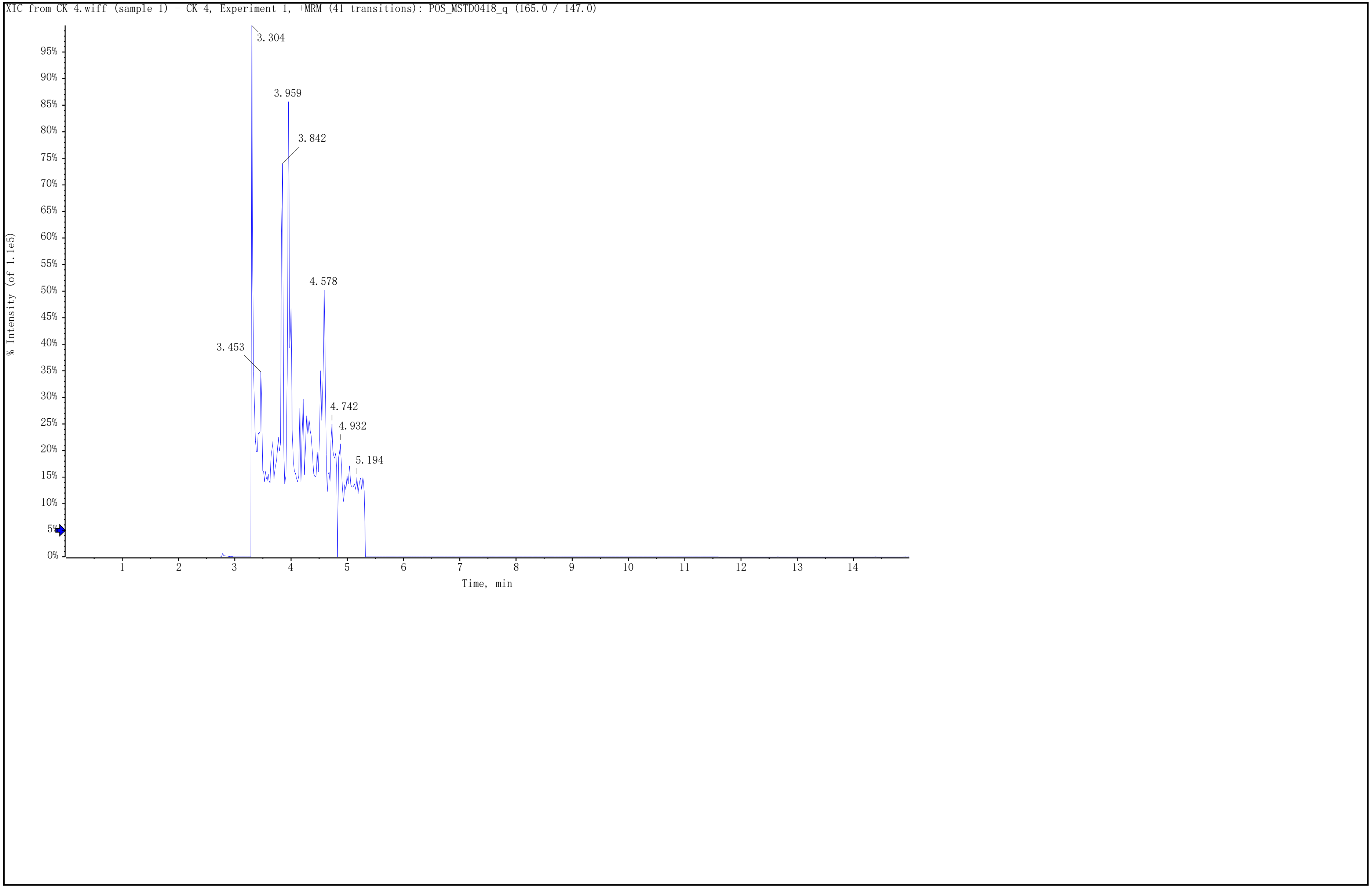

2023/5/10 15:53:50

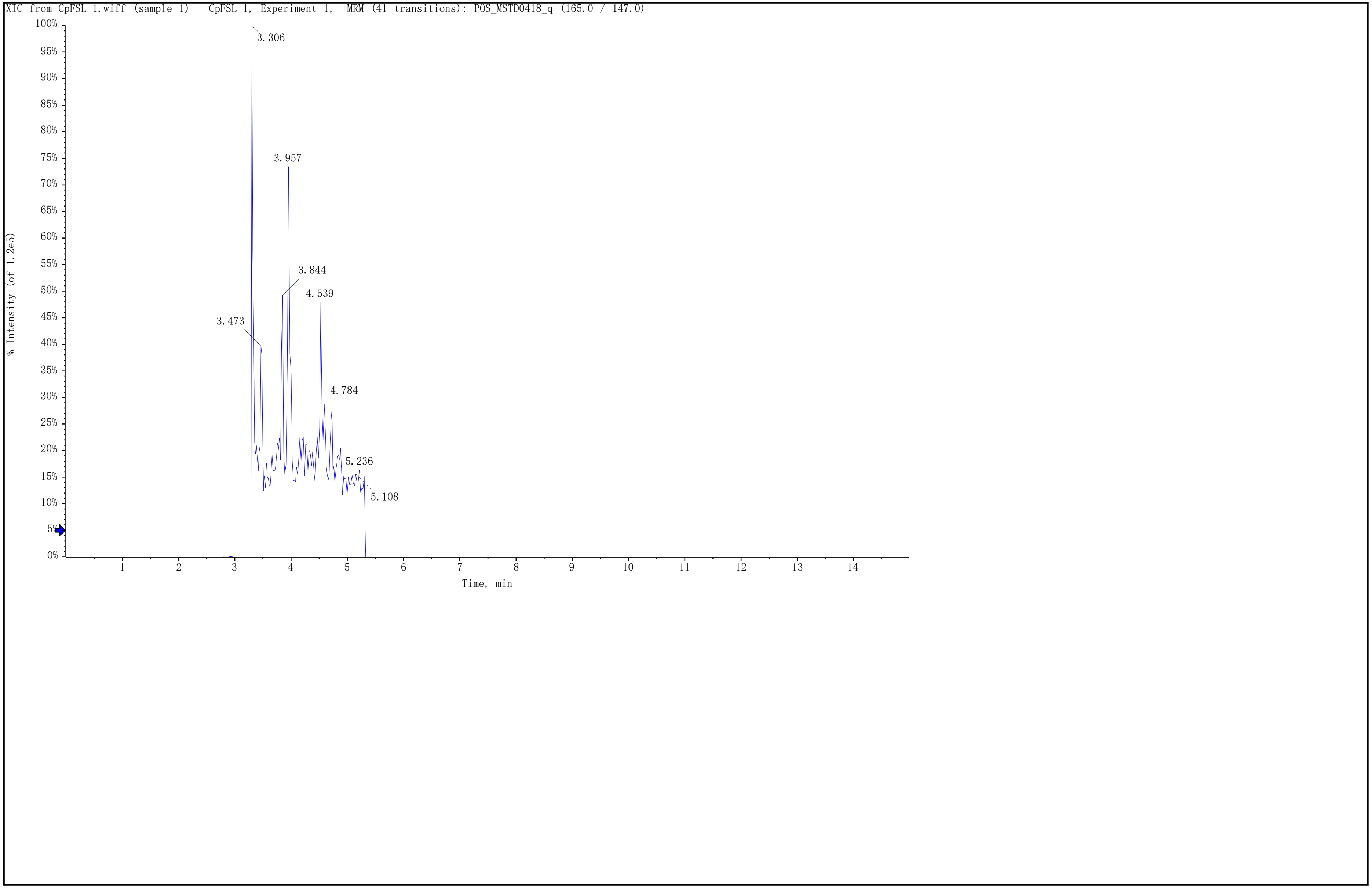

2023/5/10 16:22:46

XIC from CpFSL-4.wiff (sample 1) - CpFSL-4, Experiment 1, +MRM (41 transitions): POS_MSTD0418_q (165.0 / 147.0)

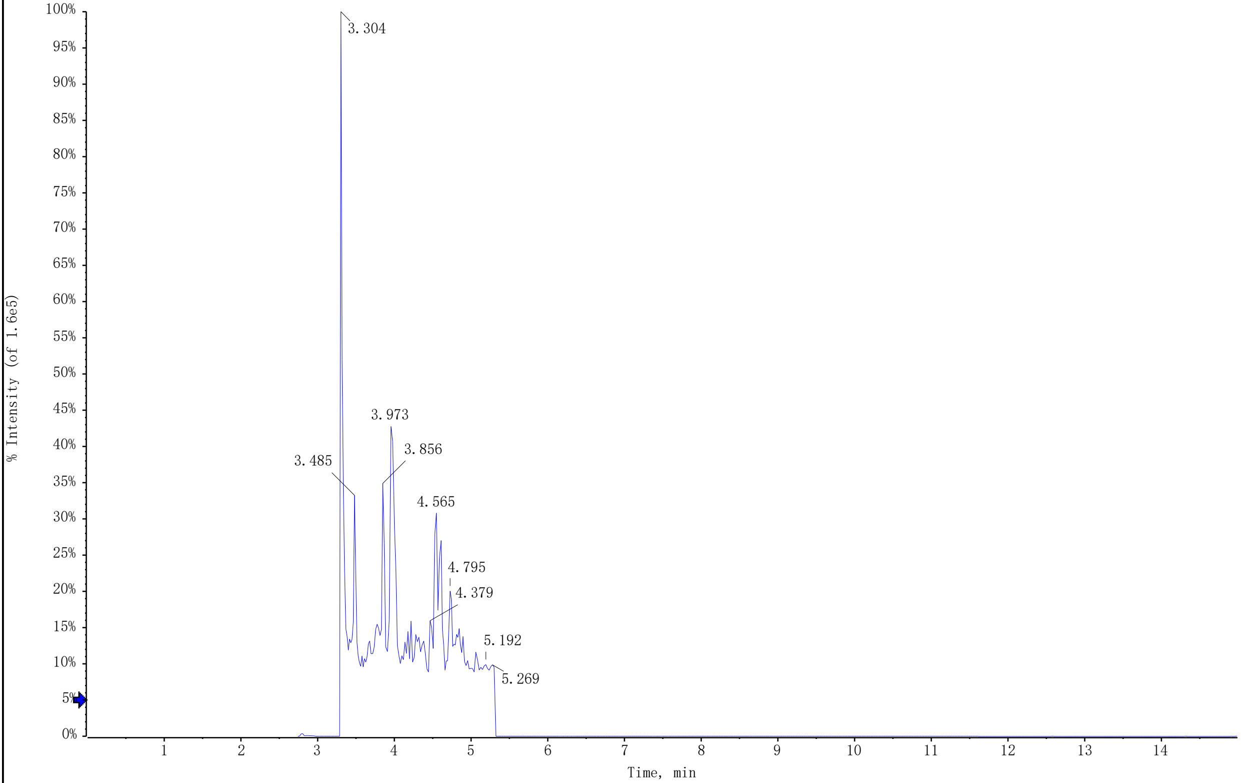

2023/5/11 16:24:20
